# Cancer-associated fibroblast subtypes modulate the tumor-immune microenvironment and are associated with skin cancer malignancy

**DOI:** 10.1038/s41467-024-53908-9

**Published:** 2024-11-08

**Authors:** Agnes Forsthuber, Bertram Aschenbrenner, Ana Korosec, Tina Jacob, Karl Annusver, Natalia Krajic, Daria Kholodniuk, Sophie Frech, Shaohua Zhu, Kim Purkhauser, Katharina Lipp, Franziska Werner, Vy Nguyen, Johannes Griss, Wolfgang Bauer, Ana Soler Cardona, Benedikt Weber, Wolfgang Weninger, Bernhard Gesslbauer, Clement Staud, Jakob Nedomansky, Christine Radtke, Stephan N. Wagner, Peter Petzelbauer, Maria Kasper, Beate M. Lichtenberger

**Affiliations:** 1 Skin and Endothelium Research Division, Department of Dermatology, Medical University of Vienna, Vienna, Austria; 2 Department of Cell and Molecular Biology, Karolinska Institutet, Stockholm, Sweden; 3 Department of Dermatology, Medical University of Vienna, Vienna, Austria; 4 Department of Plastic, Reconstructive and Aesthetic Surgery, Medical University of Vienna, Vienna, Austria

**Keywords:** Cancer microenvironment, Skin cancer, Tumour immunology

## Abstract

Cancer-associated fibroblasts (CAFs) play a key role in cancer progression and treatment outcome. This study dissects the intra-tumoral diversity of CAFs in basal cell carcinoma, squamous cell carcinoma, and melanoma using molecular and spatial single-cell analysis. We identify three distinct CAF subtypes: myofibroblast-like RGS5+ CAFs, matrix CAFs (mCAFs), and immunomodulatory CAFs (iCAFs). Large-cohort tissue analysis reveals significant shifts in CAF subtype patterns with increasing malignancy. Two CAF subtypes exhibit immunomodulatory properties via different mechanisms. mCAFs sythesize extracellular matrix and may restrict T cell invasion in low-grade tumors via ensheathing tumor nests, while iCAFs are enriched in late-stage tumors, and express high levels of cytokines and chemokines to aid immune cell recruitment and activation. This is supported by the induction of an iCAF-like phenotype with immunomodulatory functions in primary healthy fibroblasts exposed to skin cancer cell secretomes. Thus, targeting CAF variants holds promise to enhance immunotherapy efficacy in skin cancers.

## Abstract (web summary)

Fibroblast heterogeneity in the tumor microenvironment can explain their multifaceted role in cancer. Here by single-cell transcriptomic analysis of basal cell carcinoma, squamous cell carcinoma and melanoma samples, the authors explore fibroblast heterogeneity in skin cancer and their potential to modulate the tumor-immune microenvironment.

## Introduction

Fibroblasts have been considered as rather simple structural cells, which largely contribute to extracellular matrix deposition in connective tissues and tissue repair, for a long time. However, they present with an unprecedented plasticity and functional heterogeneity especially in pathological conditions, and exert a significant influence on different cells in their microenvironment, thus, modulating different processes. In cancer, fibroblasts have been established as a key component of the tumor microenvironment (TME) affecting both cancer progression and the response to therapies. Fibroblast heterogeneity has been acknowledged previously as playing both tumor suppressive as well as tumor supportive roles^1–7^. Recent studies even demonstrated that also mutations in fibroblasts can lead to cancer^8^, further highlighting their impact on tumorigenesis. It also has become clear that in a single tumor several fibroblast subtypes exist in parallel with different functions^9^. Single-cell RNA sequencing (scRNA-seq) has revealed manifold dermal fibroblast subpopulations in mouse and human healthy skin^10–12^. Likewise, fibroblast heterogeneity has been studied in many cancers such as breast cancer^13,14^, pancreatic ductal adeno carcinoma^15,16^, colorectal cancer^17,18^, head and neck squamous cell carcinoma^19^ and many more^20^. An in-depth analysis focused on fibroblast heterogeneity in skin cancer is missing. Previous scRNA-seq based studies of human melanoma, basal cell carcinoma (BCC) and cutaneous squamous cell carcinoma (SCC) mainly focused on tumor infiltrating lymphocytes and included only a few or no fibroblasts with the exception of two recent studies investigating BCC^21^ and SCC^22^ (Table S1)^21–29^. In the past years, scRNA-seq revealed a range of different cancer-associated fibroblast (CAF) subsets with newly described markers in various tissues. Besides context-dependent and/or uniquely described CAF subsets in certain types of cancer^30–32^, a common trait among many of these studies is the presence of a CAF subtype with immunomodulating characteristics, secreting IL6 and other proinflammatory cytokines, and a CAF subtype with a myofibroblast-like phenotype defined by its widely accepted signature molecule alpha smooth muscle actin (ACTA2)^13,14,33–38^.

In healthy skin, lineage tracing mouse models and functional studies in mouse and human skin identified two major fibroblast subsets, the papillary fibroblasts in the upper dermis and the reticular fibroblasts of the lower dermis. They exhibit distinct roles during skin development, wound healing, and fibrotic skin disorders^39–42^. Whether these two subpopulations evolve into CAFs, or impact skin tumor progression differently, is still an unresolved topic. So far, all studies on skin CAFs focused on CAFs as a unit, their overall marker gene expression, capability to stimulate migration of tumor cells, secretion of soluble mediators, and their response to anti-tumor therapies^43–45^, leaving a major gap in knowledge about the presence of CAF subtypes, their association with tumor malignancy and different functional roles.

In this work, we investigate the cellular ecosystem of BCC, SCC and melanoma—with emphasis on fibroblast heterogeneity—using the sensitive Smart-seq2 scRNA-seq technology (*n* = 10 tumors) and mRNA staining in situ (*n* = 68 tumors). SCCs vary from well to poorly differentiated tumors with increasing metastatic potential^46,47^, and BCCs are locally tissue destructive and metastasize very rarely^48,49^. Both cancer types arise from keratinocytes. In contrast, melanoma originates from melanocytes, and comes with a high potential for metastatic spread and poor survival rates, which have lately improved mainly due to immunotherapies^50,51^. The combined analysis of healthy skin and three distinct skin cancer types of different progression stages enabled us to dissect similarities and differences of fibroblast subsets in a premalignant and cancer context.  Collectively, our work deconstructs CAF heterogeneity in skin cancer. In particular, we show that the mCAF subtype, which forms a dense extracellular matrix network at the tumor-stroma border, likely plays a role in T cell marginalization, and propose that the iCAF subtype is an important regulator of immune cell recruitment and immune surveillance.

## Results

### A collective single-cell atlas of primary BCC, SCC, and melanoma to deconstruct skin cancer

**Fig. 1:**
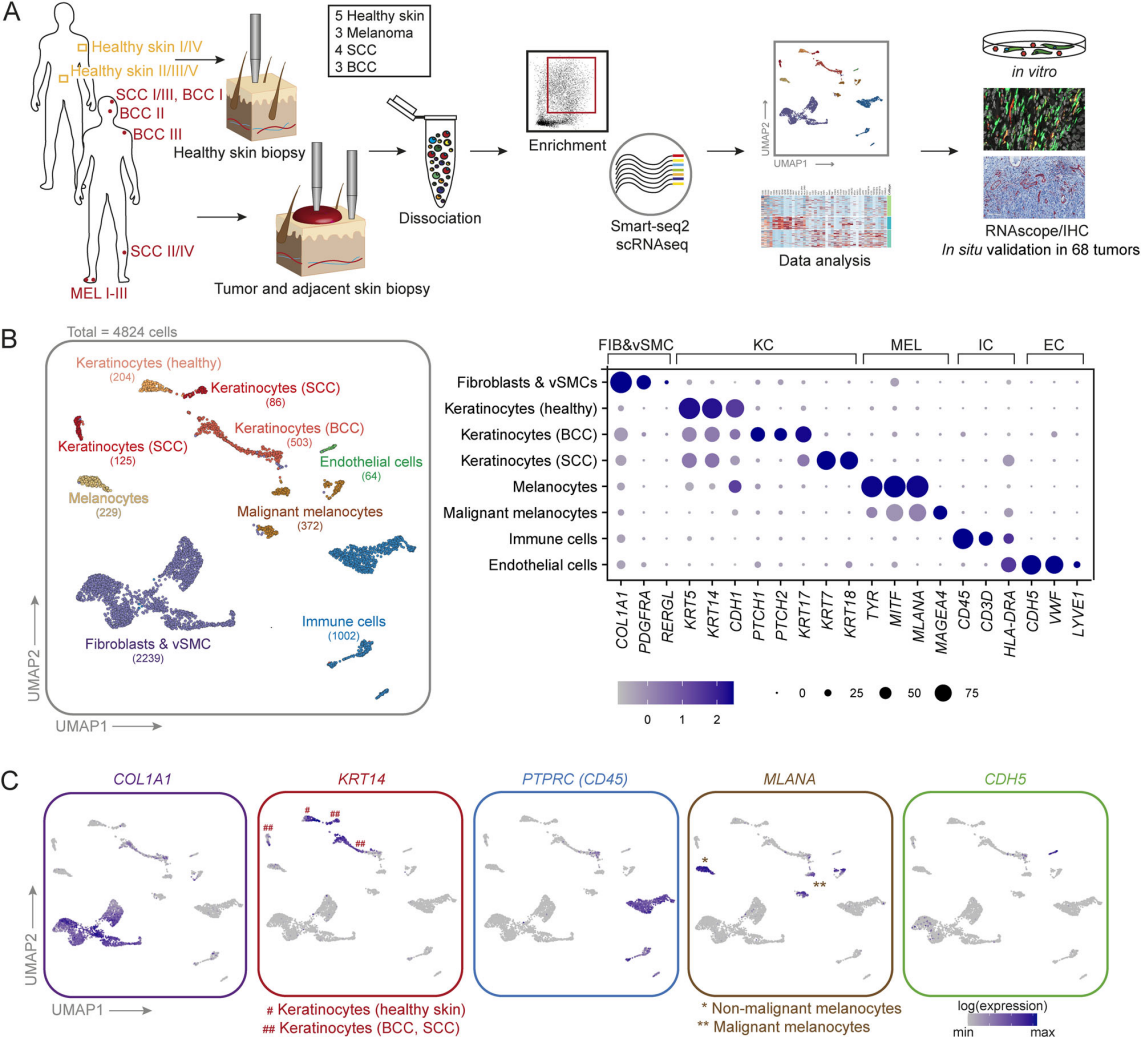
A single cell transcriptomic atlas of human BCC, SCC, melanoma, and healthy skin. **A** Workflow of donor sample processing for Smart-seq2 scRNA-seq, data analysis and verification. **B** UMAP projection of first-level clustering of 4824 cells from 15 donors (left). Clusters are labeled by cell types, which were identified by commonly accepted marker genes (right). **C** Expression of top marker genes for the main cell types.

**Fig. 2:**
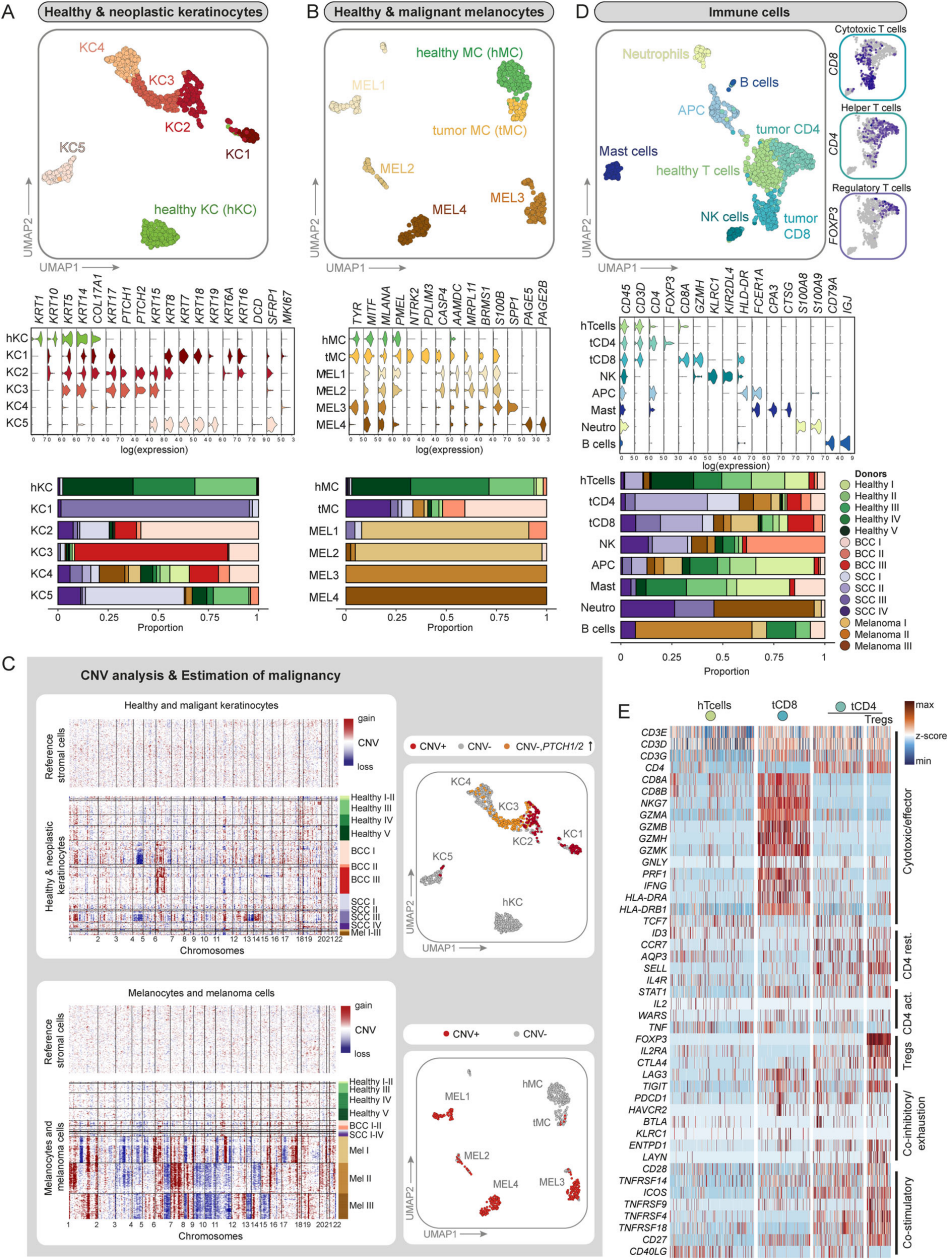
Second-level clustering of non-mesenchymal cells and CNV analysis. **A**, **B** UMAP projection of second-level clustering, violin plots of signature genes as well as bar plots showing donor sample (*n* = 15 donors) distribution per cluster are presented for healthy and neoplastic keratinocytes and melanocytes. **C** CNV analysis (based on *inferCNV* package) of tumor samples using stromal cells as reference controls. UMAPs for healthy and neoplastic keratinocytes and melanocytes: Malignant cells with a predicted CNV alteration are highlighted in red and *PTCH1*/*PTCH2* overexpressing cells without CNVs are highlighted in orange or yellow, respectively. **D** UMAP projection of second-level clustering, violin plots of signature genes as well as bar plots showing donor sample distribution per cluster are presented for immune cells. The distribution of cytotoxic, helper, and regulatory T cells are depicted in separate cut-outs. **E** Heatmap of genes that reflect the resting, activation, cytotoxic, co-stimulatory or co-inhibitors status of T cell subsets from healthy and tumor samples. KC-keratinocyte, MC-melanocyte, MEL-melanoma cells, hTcells-healthy T cells, tCD8-Cytotoxic T cells, tCD4-Helper T cells, Tregs-Regulatory T cells.

To explore fibroblast heterogeneity and their cross-talk to tumor and immune cells, we collected biopsies from four SCCs, three BCCs, three melanomas and biopsies of sun-protected skin from five healthy donors (Table S2). For the tumor samples, fresh 4 mm punch biopsies from the tumor center and from non-lesional adjacent skin (providing sex- and age-matched healthy skin controls) were collected directly after surgery. Since previous single-cell transcriptomic studies of skin cancer included no fibroblasts or only low CAF numbers (Table S1), instead of random droplet-based sampling of tumor-associated cells, we chose a FACS-sorting approach to enrich for fibroblasts and to gain highly sensitive scRNA-seq data. Upon dissociation of the tissues, the samples were enriched for keratinocytes, fibroblasts, and immune cells (Fig. S1A, B) via FACS-sorting cells directly into 384-well plates for sequencing with the Smart-seq2 technology (Fig. 1A). In two healthy control samples a random live-cell sorting approach was used, explaining why keratinocytes are underrepresented in these samples (Healthy I, Healthy II, Fig. 2A), which was compensated by a separate keratinocyte enrichment protocol in healthy samples Healthy III-V (Figs. 2A and S1A). In total, 5760 cells were sequenced, finally retaining 4824 cells at a median depth of 486146 RPKM/cell and a median of 3242 genes/cell after quality control and filtering (Fig. S1C). Cell numbers per sample after quality filtering are shown in Fig. S1D. Unsupervised clustering separated cells into fibroblasts, healthy and malignant keratinocytes as well as melanocytes, immune cells, and endothelial cells (Fig. 1B). Assignment of cell type identity was based on commonly accepted signature gene expression: *COL1A1* and *PDGFRA* for fibroblasts, *RERGL* for vascular smooth muscle cells (vSMC), *KRT5*, *KRT14* and *CDH1 (E-cadherin)* for keratinocytes, *PTPRC (CD45*) for immune cells, *TYR*, *MITF* and *MLANA* for melanocytic cells and *CDH5* for endothelial cells (Fig. 1B, C).

### Tumor cells express a range of additional keratins

Second-level clustering of healthy and neoplastic keratinocytes resulted in a clearly separated cluster of healthy keratinocytes (hKC) with basal (*KRT5*, *KRT14*, *COL17A1*) and differentiating/suprabasal (*KRT1*, *KRT10*) marker gene expression. The majority of cells in clusters KC1–KC5 contained tumor cells from BCC and SCC samples, with tumor cells expressing a range of additional keratins that are not expressed by healthy skin keratinocytes (Fig. 2A and Table S2). For example, BCC cells expressed *KRT5*, *KRT14*, and additionally *KRT17*^52^, which are foremost clustering in KC2 and KC3. As expected for the Hedgehog(Hh)-pathway-dependent BCC, we confirmed *PTCH1/2* as well as *GLI1/2* overexpression in clusters KC2 and KC3 (Figs. 2A, S2A and S2B)^53^. Cells from the SCC samples clustered in KC1, KC2, KC4 and KC5. KC1—which primarily comprised cells of donor sample SCC III (Figs. 2A and S2C)—expressed in addition to *KRT5*, *KRT14*, and *KRT17* the typical SCC keratins like *KRT6A* and *KRT16*, and showed reduced expression of *KRT1* and *KRT10*^52^ (Fig. 2A). Cells from donor sample SCC IV, a SCC arisen from Bowen’s disease (Table S2), mainly clustered in KC2, KC4, and KC5 that showed reduced expression of *KRT1*, *KRT10*. KC2 and KC5 also displayed expression of *KRT16* and *KRT19*, respectively, which has been previously described for this SCC subtype^52^ (Figs. 2A and S2C). Notably, *KRT7*, *KRT18*, and *KRT19* were only expressed by KC1 and KC5, which were primarily represented by cells originating from SCC samples (Figs. 2A and S2C). It has been shown that tumors co-opt developmental programs for their progression^1^. In our dataset we find previously described keratins of the simple skin epithelium of the first-trimester embryo such as *KRT18* and *KRT19* expressed in SCC cluster KC1 and KC5, or *KRT8* expressed in BCC cluster KC2^54^ (Fig. 2A). Interestingly, also healthy keratinocytes contributed with more than 25% to KC5, which likely represent luminal cells from sebaceous or sweat glands expressing the marker genes *KRT7*, *KRT19*, *SFRP1*, and *DCD*^21^. In KC4, keratinocytes from BCC, SCC, melanoma as well as healthy skin clustered together. These cells did not express unified classical keratinocyte markers (Fig. 2A) but presented with a scattered expression of different hair follicle-associated keratins (data not shown). This expression pattern suggests that KC4 contains keratinocytes of the different anatomical structures of the hair follicle in line with KC4 cluster’s position next to the BCC keratinocytes (KC3) and the presence of *PTCH1/2* and *GLI1/2* expressing cells (Figs. 2C and S2A-B).

### The tumor microenvironment strongly impacts non-malignant cell types

Second-level clustering of melanocytes and melanoma cells revealed one cluster of healthy melanocytes (hMC) derived from healthy skin samples, and one separate cluster of non-malignant melanocytes derived mainly from SCC and BCC samples (tumor melanocytes, tMC). This separate clustering can likely be explained by the influence of the TME on the expression pattern of melanocytes in comparison to melanocytes from healthy skin. The melanoma samples were separated into four different donor-specific clusters: MEL1 and MEL2 coming from Melanoma I, MEL3 from Melanoma II, and MEL4 from Melanoma III (Fig. 2B and Table S2). In order to explain this donor-specific clustering, as well as to segregate malignant from non-malignant cells, we inferred copy number variation (CNV) analysis on our scRNA-seq data using the R package *inferCNV*^19,27^. We performed CNV analysis for melanocytes and melanoma cells, and a separate CNV analysis for healthy and malignant keratinocytes, as previously described^19^, using healthy stromal cells (fibroblasts, vascular smooth muscle cells, and pericytes) as a reference (Figs. 2C and S3). Melanoma cells, which are known for their high mutational load^55^, displayed strong CNV patterns. Remarkably, these patterns were donor-specific despite of sharing the same histological subtype (acral lentiginous melanoma, ALM) and body location (heel/toe) (Table S1). Among BCC and SCC samples, SCC III (forming cluster KC1) presented with the strongest CNV pattern, which matched well with its histopathological characterization of a poorly differentiated, aggressive type of SCC (Table S2). In addition to the CNV analysis, we also utilized high *PTCH1* and *PTCH2* expression^56,57^ (*PTCH1/2 high)* in comparison to the healthy skin cluster hKC to pinpoint BCC tumor cells (Figs. 2A and S2A, B). As the non-malignant cells mixed across almost all donor samples, such as tMC or immune cells (Fig. 2B–D), we did not regress for donor differences; thus, donor-specific tumor cell clustering in KC1 and MEL1–MEL4 is likely the result of genomic aberrations and not an effect of batch variations.

Subclustering of immune cells resulted in eight immune cell clusters, including T and B cells, granulocytes, and antigen-presenting cells (Figs. 2D and S2C). Interestingly, CD4 and CD8 T cells from healthy samples (hTcells) formed a distinct cluster, adjacent to CD4 (tCD4) and CD8 T cells (tCD8) from tumor samples (Figs. 2D and S2C). This can be explained by a different activation status of T cells in healthy or tumor samples: Increased expression of granzymes, perforin and *IFNγ* indicated T cell activation in CD8 T cells from tumor samples versus healthy tissue (Fig. 2E). Regulatory T cells (Tregs), *CD4*^+^*IL2RA*^+^*FOXP3*^+^, were found mixed with CD4 T cells from tumor samples (tCD4) (Figs. 2D, E and S2C).

### Fibroblasts from healthy skin cluster separately from CAFs

**Fig. 3:**
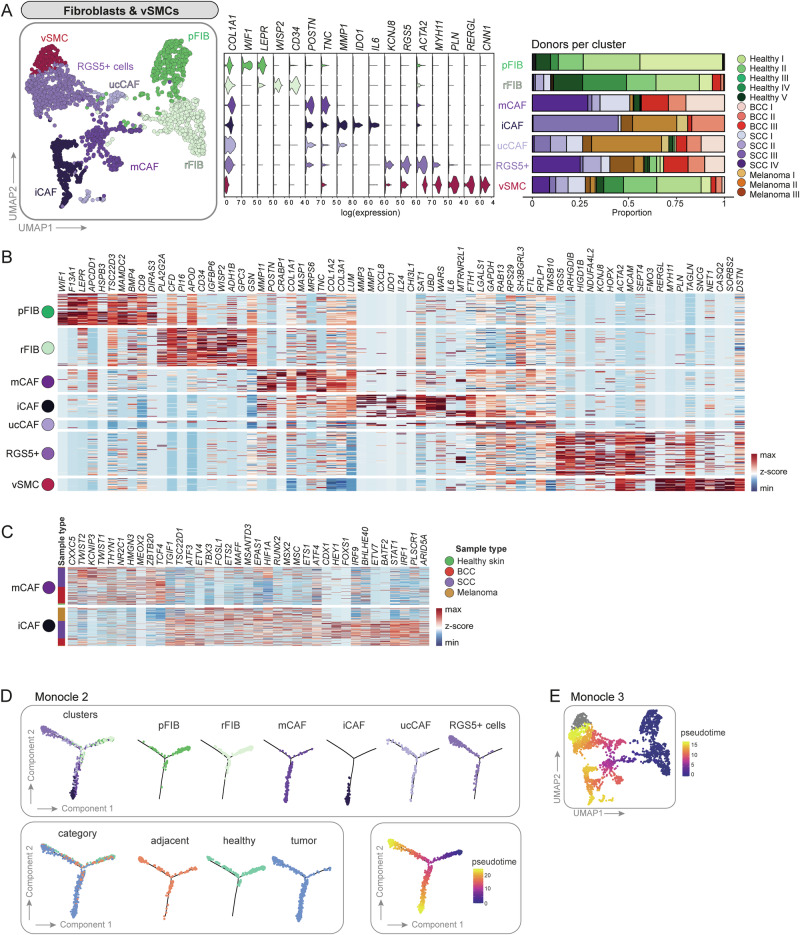
Second-level clustering of fibroblasts and vascular smooth muscle cells (vSMCs) results in two heathy fibroblasts populations, four CAF subsets and one vSMC cluster. **A** UMAP of second-level clustered fibroblasts and vSMCs. Violin plots of signature genes and bar plots showing donor sample (*n* = 15 donors) distribution per cluster. **B** Heatmap of top ten differentially expressed genes per cluster. **C** Differentially expressed transcription factors between mCAFs and iCAFs. **B**, **C** Source data of all significantly differentially expressed genes and transcription factors including exact p values are provided in the Source Data file. **D** Trajectoy analysis using Monocle2. Cells were highlighted according to clusters, category or pseudotime. **E** UMAP colored in pseudotime showing trajectory results from Monocle3.

Second-level clustering of fibroblasts and vSMCs (2239 cells) resulted in seven subclusters. We classified two main clusters from healthy skin samples called papillary fibroblasts (pFIB) and reticular fibroblasts (rFIB), four CAF subclusters called matrix CAFs (mCAF), immunomodulatory CAFs (iCAF), *RGS5*^*+*^ CAFs and pericytes (*RGS5*^*+*^ cells), unclassifiable CAFs (ucCAF), and one vSMC cluster (Figs. 3A, S4A, S4B). We used differentially expressed genes as well as commonly accepted markers to define these subclusters. Reassuringly, fibroblasts from the tumor-adjacent skin samples were found on the transition between healthy and tumor samples, and within the *RGS5*^*+*^ and vSMC clusters (Fig. S4A), indicating that CAFs may develop from skin-resident fibroblasts (pointing towards field cancerization)^58^.

Importantly, several established markers for papillary and reticular fibroblasts could be assigned to the two subclusters comprising cells from healthy donors. *COL6A5*, *COL23A1* and *HSPB3*^10^, the Wnt inhibitors *APCDD1* and *WIF1*^10,59^, *NTN1* and *PDPN*^60^ are commonly accepted markers for papillary fibroblasts and were found to be expressed in the pFIB cluster. *DPP4* (*CD26*), which was described as a marker for papillary fibroblasts by Tabib et al.^11^, was expressed in both subpopulations in our data (Fig. S4C), as shown by Korosec et al. and Vorstandlechner et al.^12,41^. The healthy fibroblast subcluster rFIB expressed genes that are characteristic for reticular fibroblasts: *THY1*^41^, *FMO1*, *MYOC*, *LSP1*^11^, *MGP*, and *ACTA2*^60^ as well as the preadipocyte marker genes *PPARG* and *CD36*^41^ (Fig. S4C). However, we could not confirm the expression of various other published reticular fibroblast markers. The discrepancy of marker expression in distinct datasets might result from tissue collection from different body sites, tissue preparation, or sequencing technology. Furthermore, several markers were identified in in vitro cultures, mostly on protein level. Thus, we conclude that the healthy fibroblast cluster pFIB represents papillary fibroblasts, and rFIB represents reticular fibroblasts (Figs. 3A, B and S4C).

### Different skin cancer types comprise both common and tumor-type-specific CAF subsets

Subclustering segregated four CAF populations: mCAFs, iCAFs, *RGS5*^*+*^ cells, and ucCAFs. Matrix CAFs (mCAFs) exhibited increased expression of extracellular matrix components such as collagens (*COL1A1*, *COL1A2*, *COL3A1)*, Lumican (*LUM*), Periostin (*POSTN*) or Tenascin-C (*TNC*) compared to all other fibroblast clusters (Fig. 3A, B). Immunomodulatory CAFs (iCAFs) presented with enhanced expression of the matrix remodelers *MMP1* and *MMP3*, the pro-inflammatory cytokines *IL6* and *CXCL8* and the immune-suppressive molecule *IDO1* among their top ten differentially expressed genes (DEGs), thus suggesting an immunoregulatory and cancer invasion-supportive phenotype (Fig. 3A, B). Intriguingly, the mCAF cluster harbored fibroblasts from all BCC and well-differentiated SCC samples (mainly SCC I and SCC IV), whereas the iCAF cluster contained fibroblasts from all melanoma samples, one poorly differentiated SCC (SCC III) and one BCC (BCC II). While the presence of these subsets seems to depend on the skin cancer type with iCAFs being associated with the most aggressive tumors, *RGS5*^*+*^ cells were found in all tumor samples independently of skin cancer type and malignancy (Figs. 3A, S4A and S4B). Notably, mCAFs and iCAFs expressed different transcription factors (TFs; Fig. 3C). In mCAFs, TFs associated with conserved developmental pathways, including genes of the WNT pathway (*CXXC5*, *TCF4*), transcriptional regulation of mesenchymal cell lineages (*TWIST1*, *TWIST2*), and anti-inflammatory signaling (*KCNIP3*), were upregulated. iCAFs expressed high levels of TFs that are related to immune responses such as *STAT1*, *IRF1*, *IRF9*, or *ARID5A*. Of note, there is also a difference in TF expression between SCC- and melanoma-derived iCAFs.

**Fig. 4:**
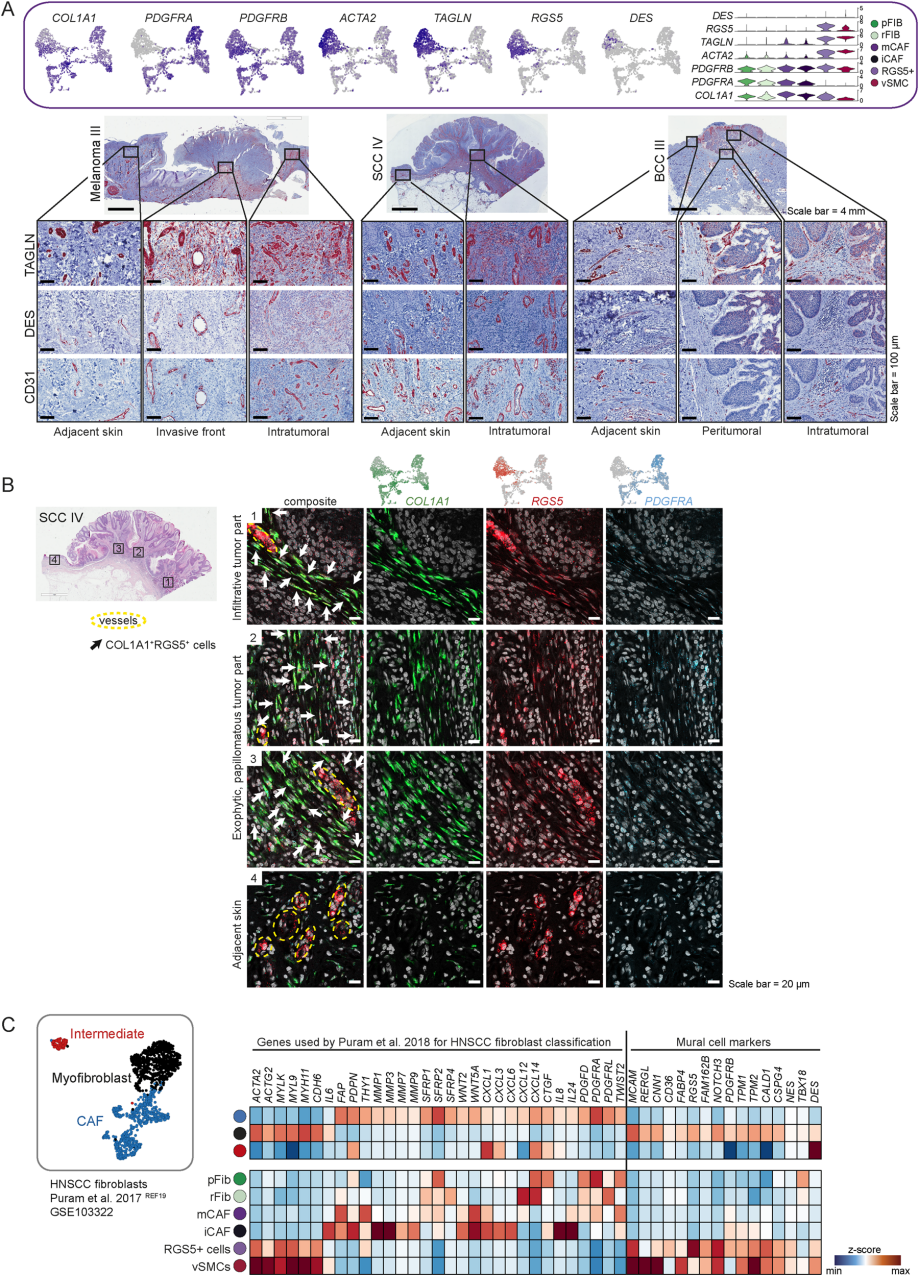
The RGS5^+^ cells are an inhomogeneous population of CAFs and pericytes. **A** Feature and violin plots showing the expression of fibroblast and pericyte marker genes in the RGS5^+^ cluster. Representative immunohistochemistry of TAGLN, DES and CD31 in different regions of the tumor (intratumoral, peritumoral) (*n* = 10 tumor samples from biologically independent donors). **B** Representative images of *COL1A1* (green), *RGS5* (red), and *PDGFRA* (blue) RNAScope fluorescence stainings in four different regions of FFPE tissue sections from donor sample SCC IV, representative for the *n* = 10 independent tumor samples. DAPI nuclear stain is shown in gray. Scale bar represents 20 μm. **C** Myofibroblasts in a HNSCC dataset from Puram et al.^19^ exhibit a very similar expression pattern in comparison to the RGS5^+^ cluster in our dataset.

Since *RGS5*^*+*^ cells expressed *ACTA2* (in combination with *COL1A1*, Fig. 4A), this subset likely represents activated fibroblasts that are usually termed myofibroblasts in wounded or fibrotic tissues^61^, or myoCAFs in different cancer types^37^. However, they also expressed genes among their top DEGs that have been used as pericyte markers, such as *RGS5*, *KCNJ8, ACTA2*, and *MCAM* (Fig. 3B)^62–64^. *RGS5*^*+*^ cells also shared some markers with the vSMC cluster, such as *ACTA2*, *TAGLN* and *MCAM*, which are markers for perivascular cells in various tissues^41,65^. The vSMC population formed its separate cluster and was clearly defined by *RERGL*, *MYH11*, *CNN1* in addition to *ACTA2*^63^ (Fig. 3A, B). Unclassifiable CAFs (ucCAFs) represented a minor population with an inconclusive gene expression pattern and mixed cell contribution from almost all tumor samples, which we therefore did not consider further for in-depth discussion (Fig. 3A, B).

### Trajectory inference shows two main differentiation routes for CAFs from healthy cells

Trajectory analysis using Monocle2 and Monocle3^66,67^ showed that healthy fibroblasts follow two differentiation routes: either towards mCAF/iCAF or towards *RGS5*^+^cells (Fig. 3D, E). Note that the vSMC cluster was excluded from trajectory inference as we do not expect them to differentiate from healthy fibroblasts and CAFs from a biological point of view. The trajectory analysis also shows that iCAFs are a differentiation endpoint, with mCAFs being an intermediate state, and thus it may be possible that iCAFs develop from mCAFs. This might also be supported by the fact that iCAFs express several mCAF genes albeit at a lower levels, while mCAFs do not express iCAF markers (Fig. S8A, B). Fibroblasts from tumor adjacent skin samples are preferentially found in the healthy fibroblast and the RGS5^+^ cells branches, and a smaller fraction bridging to the mCAF branch, indicating that they are in a transitory position between healthy fibroblasts and CAFs.

### *ACTA2* and *FAP* in combination identify all CAF subpopulations in skin tumor samples

Previous studies have used different single markers like ACTA2 or FAP to identify or isolate CAFs in different tissues^68^. However, a query on previously described CAF marker genes showed that, in our dataset, most of them are either restricted to a distinct CAF subset, or found in all fibroblast clusters including healthy fibroblasts, but do not solely identify all CAF subsets (Fig. S4D). A good strategy to detect all CAFs within skin tumor samples is combining the two most frequently used CAF markers *ACTA2* and *FAP*. Although this combination also includes vSMCs, it allows to enrich for all CAF subpopulations when used together (Fig. S4D).

### The *RGS5*^*+*^ cells are best described as a mixed population of myoCAFs and pericytes

When we investigated the detailed gene expression profiles of the distinct CAF subtypes we suspected that the *RGS5*^*+*^ cluster likely comprises both myoCAFs and pericytes, therefore we chose the neutral term “*RGS5*^*+*^ cells” for this cluster. In detail, *RGS5*^*+*^ cells expressed *ACTA2—*the signature gene for myofibroblasts and myoCAFs—in combination with *COL1A1*. The expression of *PDGFRB*, *TAGLN*, *RGS5*, *DES* and the absence of *PDGFRA* suggests that this cluster also comprises pericytes^62–64^ (Fig. 4A). Interestingly, the *RGS5*^*+*^ cluster also shows expression of *NOTCH3*, *EPAS1*, *COL18A1* and *NR2F2*, markers that were used to describe so-called vascular CAFs (vCAFs), a CAF subset defined by Bartoschek et al. in a mouse model for breast cancer^69^ (Fig. S5A). Of note, endothelial markers such as *CDH5*, *PECAM1*, *TIE1*, or *CD62* were not expressed in the *RGS5*^*+*^ cluster (Fig. S5B).

To characterize the nature of the *RGS5*^*+*^ cluster further, we stained tumor sections for Transgelin (TAGLN), which is a prominently expressed gene in this cluster. This tissue staining revealed TAGLN protein along blood vessels as expected, but also within the tumor stroma without direct contact with vessel-like structures (Fig. 4A). Protein expression of Desmin (DES), a marker for pericytes and vSMCs^61,62^ was restricted to vessels (Fig. 4A). *DES* was only expressed by few cells on RNA level, which was not sufficient to identify a separate pericyte cluster within the *RGS5*^*+*^ cells in our sequencing dataset^70,71^. Next, we performed mRNA co-staining for *RGS5, COL1A1*, and *PDGFRA* mRNA to validate our sequencing data and to verify the stromal and perivascular presence of *RGS5*^*+*^ cells (Figs. 4B and S5C). In tumor regions (Fig. 4B, region 1–3, and S4C, region 1-2), a positive staining for *RGS5* was detected both at vessel structures and within the stroma, in comparison to the peritumoral area, where *RGS5* staining was only found surrounding vessel-like structures (Fig. 4B, region 4).

To shed light on the discrepant classification of cells as being myofibroblast-like CAFs or pericytes in tumor samples, we reanalyzed a publicly available head and neck squamous cell carcinoma (HNSCC) dataset^19^ and put it in comparison with our dataset. Puram et al.^19^ classified the tumor fibroblasts into CAFs, myofibroblasts and intermediate (resting) fibroblasts. We extended their published marker gene set by commonly accepted pericyte markers and found those enriched in the myofibroblast cluster only, revealing a very similar expression profile to our *RGS5*^*+*^ cell cluster (Fig. 4C). The fact that this formerly defined myofibroblasts have been defined as pericytes upon reanalysis by another group^72^, suggests that myofibroblasts and pericytes share a very close gene expression pattern which indeed does not allow segregation by transcriptional profiling. Thus, the absence of histological stainings in previously published datasets impeded an accurate definition of those cells, and only the combination of histological localization and gene expression allows proper lineage designation. We conclude that the *RGS5*^*+*^ cell cluster within our as well as the HNSCC dataset comprises both pericytes and CAFs.

### In situ validation and spatial localization of mCAF and iCAF subsets

**Fig. 5:**
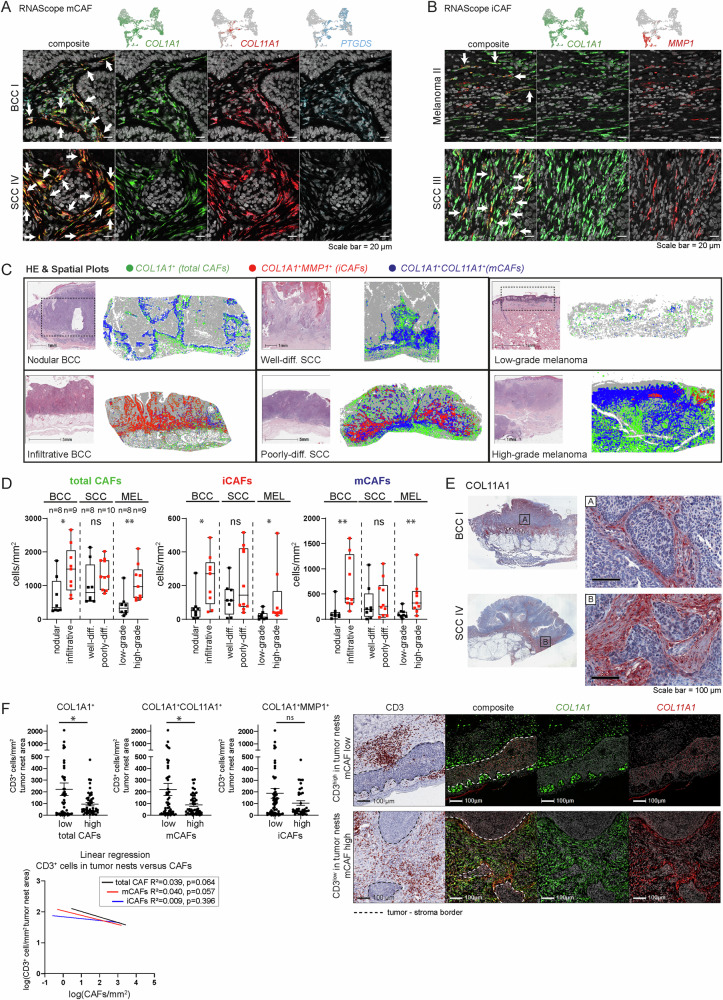
mCAFs and iCAFs are characterized by the expression of ECM and immunomodulatory genes, respectively. Representative images from (**A**) *COL1A1* (green), *COL11A1* (red), and *PTGDS* (blue) and (**B**) *COL1A1* (green) and *MMP1* (red) RNAScope fluorescence stainings to identifiy mCAFs and iCAFs respectively in FFPE tissue sections from *n* = 52 biologically independent tumor samples. DAPI nuclear stain is shown in gray. Scale bar represents 20 μm. **C** Spatial plots highlighting the spatial distribution of total CAFs (*COL1A1*), iCAFs (*COL1A1*^*+*^*MMP1*^*+*^*)* and mCAFs (*COL1A1*^*+*^*COL11A1*^*+*^) and respective H&E stainings on consecutive sections. Dashed-lined boxes show approximate area of spatial plot in H&E stainings. **D** Quantification of total CAFs (*COL1A1*^+^), iCAFs (*COL1A1*^+^*MMP1*^+^), and mCAFs (*COL1A1*^+^*COL11A1*^+^*MMP1*^−^) in cells per mm^2^ in 52 independent tumor samples of nodular (*n* = 8) and infiltrative BCC (*n* = 9), well (*n* = 8) and poorly (*n* = 10) differentiated SCC as well as low- (*n* = 8) and high-grade (*n* = 9) melanoma. Fibroblast numbers of at least five representative ROIs from each tumor were summed-up and normalized to the tissue area to capture the whole tumor tissue. Data are presented as box plots with median as center and whiskers ranging from minimum to maximum, bounds of boxes extend from the 25th to 75th percentiles. Statistical analysis by two-sided Mann-Whitney test. *p < 0.05, **p < 0.01. **E** Representative images from *COL11A1* immunohistochemistry stainings (*n* = 10 biologically independent tumor samples). Scale bar represents 100 μm. **F** Image analysis of CD3^+^cells/mm^2^ in tumor nests and total CAFs/mm^2^ (high-low cutoff 140 cells/mm^2^), mCAFs/mm^2^ (high-low cutoff 40 cells/mm^2^), or iCAFs/mm^2^ (high-low cutoff 40 cells/mm^2^) in 97 ROIs from nodular and infiltrative BCCs (*n* = 15 biologically independent tumor samples). Linear regression analysis of log(CD3^+^cells/mm^2^) in tumor nests and log(CAFs/mm^2^). Representative images of CD3 immunohistochemistry and *COL1A1* (green) *COL11A1* (red) RNAScope fluorescence stainings. Data are presented as mean values ± SEM. Statistical analysis by unpaired two-sided t-test; *p < 0.05. **D**, **F** Source data and exact *p* values are provided in the Source Data file.

**Fig. 6:**
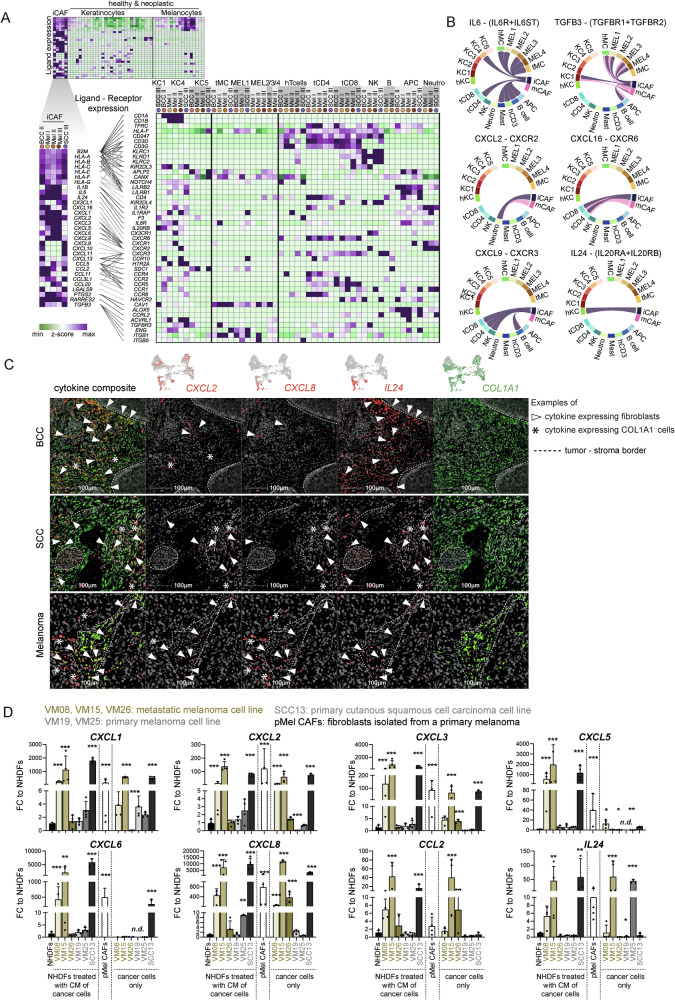
Fibroblasts are an important source of chemokines in the tumor. **A** Expression of immunomodulatory genes in iCAFs compared to healthy and neoplastic keratinocytes and melanocytes and interrogation for respective receptors in healthy and neoplastic keratinocytes and melanocytes as well as immune cells (*n* = 15). **B** Circular plots of selected receptor-ligand pairs from CellChat analysis, showing mCAF/iCAF as source cells (*n* = 15 donors). **C** Representative images of RNA ISH staining with probes against *CXCL2*, *CXCL8*, *IL24*, and *COL1A1* of BCC, SCC and melanoma samples (*n* = 43 biologically independent tumor samples). **D** In vitro cytokine expression of NHDF after exposure to conditioned medium from NHDFs, VM08, VM15, VM26, VM19, VM25, and SCC13 cells for 72 h in comparison to the cytokine expression of the cancer cell lines VM08, VM15, VM26, VM19, VM25, and SCC13, and to primary melanoma-derived CAFs (pMel CAFs). Data from four independent experiments are presented as bar graphs showing mean values ± SD, overlayed with individual data points of the independent experiments. Statistical analysis by One-way-ANOVA and Tukey’s post hoc test for multiple comparison on log-transformed data. Significant comparisons to NHDFs are shown; Source data and exact *p* values are provided in the Source Data file. *p < 0.05, **p < 0.01, ***p < 0.001.

We verified the presence of iCAFs and mCAFs by mRNA staining in situ in the same tumor samples that were sequenced as well as in additional independent tumor biopsies (*n* = 68 tumors in total). We used *COL11A1* and *PTGDS* as markers for mCAFs, and MMP1 (and several cytokines) as a marker for iCAFs, in co-stainings with the pan-fibroblast marker *COL1A1* (Figs. 5A, B, 6C, S6 and S7). The distribution of mCAFs and iCAFs in the tumor tissue follows different patterns: mCAFs were found abundantly in large patches ensheathing tumor nests, but also pervading the tumor in strands (Figs. 5A and S6A). Importantly, mCAFs were especially enriched at the tumor-stroma border of BCC and well-differentiated SCC (Fig. S6A). Contrary, iCAFs were found in smaller numbers intermingled between *MMP1*^-^*COL1A1*^+^ cells intratumorally in stromal nests and strands that pervade the tumors or in patches at the invasive front (Figs. 5B, S6B). To verify our scRNA-seq data suggesting that iCAFs are predominant in aggressive tumors, we stratified the tumor samples into different categories: nodular and infiltrative BCC, well-differentiated and poorly-differentiated SCC, and low-grade (Tis and ≤T1) and high-grade (≥T3) melanomas (n = 52). Large-field spatial visualization of the CAF subpopulations in tumor tissue samples showed a clear change in the CAF patterns from lower to higher malignancy, along with a higher overall CAF density in the aggressive variants of the respective skin cancer subtypes (Fig. 5C). To quantify this difference in CAF subsets, the regions of interest (ROIs) were set within the tumor as well as at the invasive front (Figs. 5C, D and S7A; see Methods). The total CAF density significantly increased in infiltrative BCC compared to nodular BCC, and in high-grade melanoma compared to low-grade melanoma (Fig. 5D). Also the iCAFs displayed an increase in number between nodular BCC and infiltrative BCC, and low grade (≤T1) and high grade (≥T3) melanomas, respectively (Fig. 5D). The SCC subtypes displayed a similar iCAF trend; however, it was not statistically significant (see Discussion). This extended data analysis, which also included infiltrative BCC and low-grade melanoma samples, confirmed the scRNA-seq data showing that iCAFs are more abundant in more malignant skin cancer subtypes, particularly in infiltrative BCC and high-grade melanoma. Interestingly, also the mCAFs increased in abundance in infiltrative BCC compared to nodular BCC, and high grade (≥T3) versus low grade (≤T1) melanomas (Fig. 5D, Discussion).

### mCAFs form a barrier around tumor nests

We investigated the expression of matrix-associated genes (collagens, laminins, lysyloxidases, and other ECM genes) and immune response or cancer invasion-associated genes (MMPs, chemokines, interleukins, and immunomodulatory genes) by module scores and found a significant enrichment of matrix-associated genes in mCAFs and immuno/invasiveness-associated genes in iCAFs (Fig. S8A and S8B). Additionally, we interrogated for possible ligand-receptor interactions: mCAFs exhibit a strong expression of collagens and other ECM genes, whose receptors are found on healthy and neoplastic keratinocytes and melanocytes as well as on immune cells (Figs. S9A and S9B). COL11A1 protein staining revealed a dense network of collagen fibers aligning the basement membrane of tumor nests (Fig. 5E), indicating that mCAFs may control T cell marginalization as has been shown for COL11A1 expressing CAFs in lung cancer^73^. Thus, we quantified the number of mCAFs and CD3^+^ cells in the total cancer tissue (including tumor stroma) and the number of CD3^+^ cells within tumor nests of several ROIs per sample of nodular and infiltrative BCC samples (*n* = 15, 97 ROIs). The number of mCAFs/mm² tissue negatively correlated with CD3^+^ cells/mm² within tumor nests (Fig. 5F), suggesting that mCAFs may form a physical barrier to inhibit T cell infiltration into tumor nests. Of note, while total CAF and mCAF numbers negatively correlated with CD3 cells/mm² in tumor nests, iCAF numbers did not (Linear regression: total CAFs: *R*² = 0.039; mCAFs: R² = 0.040; iCAFs: *R*² = 0.009). Indeed, representative images from overlaid CD3 and mCAF stainings (COL1A1^+^COL11A1^+^) showed that infiltration of CD3^+^ cells foremost occurs at areas where staining of mCAFs is low or absent (Fig. 5F).

### iCAFs are the major source of cytokines in the TME and are capable of activating T cells

iCAFs strongly expressed immunomodulatory genes, including *TGFB3* and *LGALS9*, proinflammatory cytokines such as *IL1B* and *IL6* (Figs. 6A and S8B). Additionally, iCAFs expressed high levels of a plethora of chemokines in comparison to healthy or neoplastic keratinocytes and melanocytes (Fig. 6A, upper heatmap), and thus likely regulate the immune cell composition and influence immune surveillance in the tumor as their receptors are found on many different immune cells (Fig. 6A, B). Notably, iCAFs from melanoma samples expressed high levels of *CXCL1-8* but not *CXCL9-13*, whereas the expression of *CXCL9-13* is high in iCAFs of the SCC III and BCC II samples. Only *CXCL2* was equally high expressed in iCAFs from all tumor samples (Fig. 6A). Similarly, *IL1B* was expressed at much higher levels in iCAFs derived from melanoma, while *TGFB3* and *LGALS9* were strongly expressed in iCAFs from BCC and SCC but not melanoma (Fig. 6A). We also made a receptor-ligand interrogation with CellChat^74^ and confirmed several predicted interaction partners of iCAFs and mCAFs as a signaling source (Figs. 6B and S9). We further confirmed the CAF-derived expression of cytokines by mRNA stainings in situ (Figs. 6C, S7B). *CXCL2*, *CXCL8* and *IL24* were selected for the analysis because these three cytokines showed a good coverage across the iCAF cluster, although cell- and sample-specific differences remain (Fig. S7B, C). As visualized in the spatial plots of representative samples, cytokine-expressing CAFs are more abundant in the most aggressive tumor variants (infiltrative BCC, poorly differentiated SCC, and high-grade melanoma) (Fig. S7B). While the majority of nodular BCC and low-grade melanoma harbored no or single dispersed cytokine-expressing CAFs, several infiltrative BCC and high-grade melanoma presented with multiple clusters of cytokine-expressing CAFs (Fig. 6C), which is in line with the in situ quantification of iCAFs (Fig. 5C, D) and transcriptomic data (Fig. S4A). The difference in cytokine-expressing CAF density and distribution was not as pronounced between well and poorly-differentiated SCCs, although they appeared to be more frequent in late-stage SCC (Figs. 5C, 6C and S7B). Furthermore, we confirmed that CAFs are a major source of cytokines in the TME in an entirely independent single-cell transcriptomics dataset of melanoma (*n* = 5) (Fig. S8C). These samples express exceptionally high levels of *CCL2*, *CXCL12*, and *CXCL14*. Along these lines, also CAFs from oral SCCs display stronger cytokine expression than their respective tumor cells (Fig. S8D).

These results led us to hypothesize that the cancer cells of invasive cancers (but not from non-invasive ones) may directly impact the phenotype of tumor-adjacent fibroblasts. To test this, we isolated primary dermal fibroblasts from healthy skin (NHDF) and treated them with conditioned medium collected from melanoma and SCC cell lines (Fig. S10A). Intriguingly, the conditioned medium (CM) of cultured cell lines derived from melanoma metastases (VM08 and VM15 from lymph node metastases) or from a highly aggressive SCC (SCC13)^75^ strongly induced the expression of different cytokines and chemokines in NHDFs (Fig. 6D). Likewise, these cytokines and chemokines were expressed at high levels by fibroblasts isolated from a primary melanoma without further treatment (pMel CAFs; Fig. 6D). On the contrary, VM19 and VM25 cell lines, which were derived from primary melanomas, did not induce cytokine expression (except CXCL8 by VM25). The melanoma cell line VM26 derived from a subcutaneous metastasis^76^ only induced higher levels of *CCL2* but not the other tested cytokines and chemokines. Although the cancer cells expressed several cytokines themselves (Fig. 6D), it was striking that the supernatant of these cancer cell lines induced even more cytokines and chemokines in healthy fibroblasts. Importantly, we confirmed the expression of several cytokines and chemokines by fibroblasts and induced iCAFs on protein level with LEGENDplex assays (Fig. S11). Of note, the increased expression of cytokines and chemokines in NHDFs treated with VM08 and VM15 conditioned medium, at the RNA level was not consistently reflected at the protein level. This is likely attributed to the fact that for the LEGENDplex assay, cells were without stimulus for two days prior to the analysis of their supernatant.

Intriguingly, while the conditioned medium from cancer cells alone induced the expression of iCAF-related genes (cytokines, chemokines, Fig. 6D), the expression of ECM-related genes was not induced (Fig. S10B). Thus, we conclude that the secretome of invasive tumor cell lines can transform normal fibroblasts into iCAF-like, but not mCAF-like cells in vitro.

**Fig. 7:**
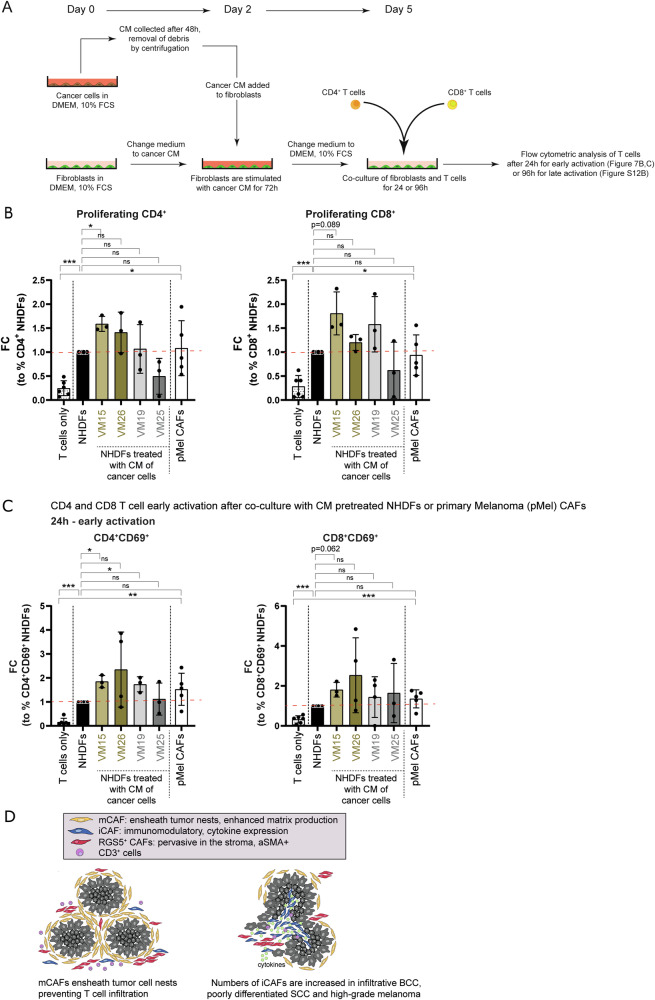
Fibroblasts activate CD4^+^ and CD8^+^ T cells. **A** Experimental setup of T cell assays shown in B and C. **B** Proliferation assessed by flow cytometry of CD4 or CD8 T cells upon co-culture with NHDFs pre-treated with conditioned medium from cancer cells, primary melanoma-derived CAFs (pMel CAFs) or cancer cells. **C** Upregulation of the early activation marker CD69 on CD4 or CD8 T cells after 24 h of co-culture with NHDFs pre-treated with conditioned medium from cancer cells, pMel CAFs or cancer cells. Data represented as fold change of percentages of cells positive for the indicated markers normalized to NHDFs. **B**, **C** *n* = 6 biologically independent samples for T cells only and untreated NHDFs, *n* = 5 biologically independent samples for pMel CAFs, and *n* = 3 biologically independent samples for T cells in co-culture with VM15, VM26, VM19, VM25. Data are presented as mean values ± SD. Statistical analysis in comparison to NHDFs or to T cells only by unpaired two-sided Student’s *t* test and Welch’s correction; **B**, **C** Source data and exact *p* values are provided in the Source Data file. *p < 0.05, **p < 0.01, ***p < 0.001. **D** Schematic summary of spatial distribution of distinct CAF subsets in human skin cancer.

Furthermore, naïve CD4 and CD8 T cells were co-cultured together with NHDFs that were pretreated with conditioned medium from VM15, VM26, VM19, VM25, or control medium, and with pMEL CAFs. In parallel, naïve CD4 and CD8 T cells were co-cultured with the cancer cells directly. We found that primary fibroblasts isolated from healthy skin are capable of activating T cells (Figs. 7A–C and S12A), as shown by increased percentages of proliferating CD4 and CD8 T cells (Fig. 7B) and activated CD69 + CD4 and CD8 T cells (Figs. 7C, S12A, B). This potential to activate T cells was enhanced when fibroblasts were exposed to the secretome of specific cancer cells. Comparing cancer CM-treated NHDFs to untreated NHDFs showed a further increase in CD4 T cell proliferation with CM derived from VM15 and an increase-trend with CM derived from VM26 (not statistically significant). Also, CD8 T cell proliferation with CM derived from VM15, VM26, and VM19 showed a comparable trend, albeit not statistically significant. Early T cell activation was promoted by VM15- and VM19-derived CM for CD4 T cells, and by VM15-derived CM for CD8 T cells (Fig. 7C). Late activation of CD4 + T cells measured as percentage of CD45RO+/CD62L- T cells at 96 h was significantly enhanced by CM derived from VM15, VM26 and VM19 (Fig. S12C, D). Importantly, also CAFs directly isolated from a primary melanoma without further treatment (pMel CAFs) were potent in activating CD4 and CD8 T cells (Fig. 7B, C).

Taken together, in situ stainings of the marker genes identified in our scRNA-seq screen showed that mCAFs and iCAFs are distinct CAF populations that follow different distribution patterns in situ (Fig. 7D*)*. mCAFs are present in all tumors but seem to play an important role at the tumor-stroma border as they form dense networks surrounding tumor nests of benign tumors, i.e., nodular BCC and well-differentiated SCC. In contrast, the number of iCAFs increases significantly in aggressive tumors (especially infiltrative BCC and late-stage melanoma) and, thus, high abundance of iCAFs correlates with malignant progression (Figs. 5D and 7D). Importantly, receptor-ligand analysis revealed that iCAFs, which are associated with late-stage tumors, express many immunomodulatory factors that bind to receptors expressed primarily on neutrophils, T cells and NK cells. Notably, the heat map in Fig. 6A shows that apart from immune cells, fibroblasts synthesize the majority of cytokines and chemokines but not tumor cells, indicating that not the cancer cells but the stromal cells (fibroblasts) are key players in immune cell recruitment and activation. Indeed, we confirmed that fibroblasts treated with the secretome of skin cancer cells are capable of activating T cells. Furthermore, since mCAFs ensheath tumor nests and synthesize large amounts of ECM proteins, it is likely that they are involved in T cell exclusion (Fig. 5E, F).

## Discussion

Fibroblasts are important contributors to the TME. They can exert pro- as well as anti-tumorigenic functions by stimulating tumor cell survival and proliferation, modifying ECM stiffness, supporting metastasis, influencing therapy response, regulating immune cell recruitment via chemokine secretion and inflammatory responses^38,77^. Inter- and intratumoral CAF heterogeneity has been appreciated ever since scRNA-seq methods have become available. However, their different functions in the TME remain largely inexplicit. The scRNA-seq-based works that have been published for skin cancers, four melanoma studies^23,25–27^, one SCC study^24^ and one study containing BCCs and SCCs^28^, either only contained a very small number of fibroblasts or fibroblast heterogeneity was not the focus of the analysis. Considering the large knowledge about fibroblast diversity in healthy human dermis^10–12,41^, a screen on the CAF heterogeneity in human skin tumors was required to fill the missing gap, which we achieved with the present work, especially since our dataset comprises the three major skin cancer types, and because we validated our findings not only in other published datasets but also by comprehensive spatial analysis of the different CAF subsets in situ.

We have previously shown that healthy human skin comprises two functionally distinct fibroblast subsets (papillary and reticular fibroblasts) which can be distinguished by the expression of *CD90* (*THY1*)^41^. The present scRNA-seq screen confirmed that *CD90*, which is still frequently used as the sole fibroblast marker to isolate or visualize skin fibroblasts, is only expressed by the reticular subpopulation (Fig. S4C). Although the majority of CAFs express *THY1* (CD90) (Fig. S4D), our data do not allow to conclude whether CAFs develop only from skin-resident reticular fibroblasts or whether they acquire expression of *CD90* upon activation.

Of note, the RNA expression level of *FAP* in the scRNA-seq data does not reflect the FAP protein expression on the fibroblasts entirely, as we used FAP and CD90 surface expression to enrich for fibroblasts by FACS (Figs. S1B and S4C).

In the skin cancer samples, we identified three distinct fibroblast populations (excluding the small population of ucCAFs). Immunomodulatory CAFs (iCAFs) show a characteristic expression of proinflammatory cytokines (*IL1B*, *IL6*), chemokines (CXCR2 ligands) and immunomodulating molecules (*IDO1*) and, thus, seem to be analog to the previously described iCAFs in other cancer types (Figs. 3B, 6A, B)^37^. Matrix CAFs (mCAFs), which we identified as a separate CAF population, are not to be confused with myofibroblasts described in other publications^37^. mCAFs exhibit increased matrix production but without the expression of *ACTA2* and myosin light chain proteins (Figs. 3B, S8A, B).

Most interestingly, our dataset suggested that iCAFs and mCAFs can likely be attributed to skin cancer types with higher (iCAFs) and lower (mCAFs) metastatic potential. The only exception were fibroblasts from BCC II, which were found in the iCAF cluster even though BCC II is histologically not different to the other two BCC samples (Fig. S4B). Quantification of the distinct CAF subsets was particularly challenging because of the varying nature of skin cancer. Skin tumors display heterogeneous morphology both within a single tumor (i.e., superficial tumor areas versus invasive front) and among distinct cancer subtypes. Thus, the biological difference may not be well captured in numbers. Large-field spatial visualization of the CAF subsets in tumor tissue samples showed an obvious difference in the CAF patterns from lower to higher malignancy, along with a higher overall CAF density in the more aggressive variants of the respective skin cancer subtypes (Fig. 5C,D). In situ localization revealed that mCAFs are present in all tumors but are detected in high density at the tumor-stroma border especially in nodular BCC and well-differentiated SCC. That mCAFs also increase in number in late-stage tumors (Figs. 5, S6A and S7) is interesting in connection with the results of our multiplex mRNA staining in situ. Future spatial transcriptomic analysis could reveal if the mCAFs located at the tumor-stroma border have a distinct expression profile compared to the mCAFs located within the stromal strands and without direct contact to tumor cells. This may indicate, for example, dual functions of mCAFs or a potential conversion of tumor ensheathing-CAFs towards a “more aggressive” iCAF-like phenotype both of which are an exciting future route to explore.

iCAFs are more abundant in malignant tumors, especially in infiltrative BCC and in aggressive melanomas (Fig. 5B,C). Although there is a trend that poorly differentiated SCCs harbor higher numbers of iCAFs compared to well-differentiated SCCs, the difference is not so clear between these two cancer subtypes. Analysis of larger patient cohort with additional stratification into further subtypes may be necessary to provide a clearer picture.

The *RGS5*^*+*^ cluster contains cells from all samples, including healthy controls. According to differential gene expression, the *RGS5*^*+*^ cluster would commonly be termed as myofibroblasts/myoCAFs due to the characteristic expression of *ACTA2*, *MYH11*, and *COL1A1*. However, the *RGS5*^*+*^ cluster also showed a marker profile that can be attributed to pericytes (*RGS5*, *PDGFRB*, *KCNJ8*, *TAGLN, MCAM*) (Figs. 3B and 4A). We demonstrate by in situ immunohistochemistry that *RGS5*^*+*^ cells are not only found in perivascular localization but are also distributed throughout the stroma in tumor regions. In contrast, in unaffected skin, *RGS5*^*+*^ cells are restricted to a perivascular localization (Fig. 4A, B). Thus, an exclusive definition as myofibroblasts/myoCAFs or pericytes for this cluster seems to be inappropriate. The impossibility to discern myofibroblasts/myoCAFs from pericytes on RNA level has been a general issue, as we found very similar expression patterns of the myofibroblasts/pericytes in a HNSCC dataset, once defined as myofibroblasts^19^, and once defined as pericytes^72^ (Fig. 4C). It has been suggested that pericytes are able to leave the vessel wall and contribute to the tumor stroma in a process called pericyte-fibroblast transition (PFT)^78^. To our knowledge, PFT has not been shown in skin cancer. A recent pan-cancer scRNA-seq study suggested that a small CAF subset arose from endothelial cells. However, this was concluded from transcriptional data but not confirmed in situ^79^. Whether *RGS5*^+^ CAFs originate from pericytes or skin-resident fibroblasts cannot be concluded from this study. It is of higher importance that *RGS5*^*+*^*ACTA2*^*+*^ CAFs are present in all tumor samples, which might represent a general phenotype that is similar to activated fibroblasts expressing *ACTA2* in non-cancerous conditions, such as wound healing^80,81^. Notably, Grout et al.^73^ recently described an *MYH11*^+^*aSMA*^+^(*ACTA2*^+^) and *COL4A1* expressing CAF subset in non-small cell lung cancer that might be involved in T cell exclusion. The *RGS5*^*+*^ CAFs in our study (which we appointed a T cell-exclusion role from tumor nests) also expressed *MYH11*, *ACTA2*, and *COL4A1*.

Reanalysis of published datasets from HNSSC^18^ and cutaneous SCC^21^ confirms the presence of RGS5^+^ CAFs in both SCC types (Fig. S13A, B), and revealed CAF subsets with expression patterns similar to mCAFs and iCAFs in cutaneous SCC (Fig. S13B, CAF1 and CAF2). In HNSCC, the CAF1 subset displayed expression of both mCAF and iCAF genes (Fig. S13A). However, in the cutaneous SCC dataset, CAFs from one patient are overrepresented (>50% of total fibroblasts; 92% of CAF1 and 70% of CAF2 subsets), which impedes to deconstruct if CAF1 or CAF2 are more or less abundant in moderate and well-differentiated SCCs. We also confirmed the presence of all three CAF subsets in an independent melanoma dataset (Fig. S8C). Moreover, reanalysis of single-cell transcriptomic data of 5 infiltrative BCCs^29^ confirmed the presence of mCAFs and a small cluster of iCAFs (Fig. S13C). Furthermore, the samples included a fibroblast cluster expressing signature genes of reticular fibroblasts (Fig. S13C). Of note, also in our dataset few cells from BCC and SCC contributed to the rFIB cluster, most of them however from unaffected skin adjacent to the tumors (Fig. S4A). However, RGS5^+^ cells were not included in their CAF population but might be part of the pericyte population, which clustered separately in their first level clustering^29^. Ganier et al. identified two clusters of *RGS5*+ and *TAGLN*+ pericytes in healthy skin and BCC^21^. They detected a selective expansion of *RGS5*+ pericytes and a reduction in *TAGLN*+ pericytes in BCC compared to healthy skin, and described that the colocalization with vessel-like structures is lost in BCC, indicating that these cells are similar to the *RGS5* + *TAGLN* + CAFs described in our study. Furthermore, they detected four fibroblasts subsets which were designated as *APOD+*, *SFRP2*+, *PTGDS*+, and *POSTN*+. Intriguingly, both *POSTN*+ and *PTGDS* + CAFs were detected around the tumor islands, suggesting that these CAFs correspond to the mCAFs in our study as we used *COL11A1* and *PTGDS* to localize them in the tissue (Fig. 5A).

CAFs share common features with fibroblastic reticular cells within lymph nodes, which generate ECM conduits to guide the traffic of immune cells and the transit of potential antigens^82^. It is established that CAFs participate in T cell exclusion from tumor nests^83^. Several studies have reported reduced T cell infiltration in CAF-rich tumors compared to their CAF-low counterparts^84^. mCAFs are detected at the tumor-stroma interface and ensheath tumor nests, especially in nodular BCC and well-differentiated SCC. As they synthesize a range of ECM proteins including COL11A1, which we detected as dense fibers surrounding tumor nests, we propose that mCAFs play a crucial role in T cell marginalization. Indeed, we detected a negative correlation between T cell numbers present in tumor nests and the number of mCAFs surrounding the tumor nests (Fig. 5F). A similar function was described for a subset of FAP^+^ αSMA^+^ lung CAFs expressing COL11A1/COL12A1 or COL4A1 in human lung cancer^73^. Thus, targeting mCAFs may improve the efficacy of immunotherapy in patients bearing T cell-excluded tumors. Indeed, a whole tumor cell vaccine genetically modified to express FAP significantly reduced cancer growth in a murine model of lung cancer and melanoma by directly inhibiting CAFs and simultaneously enhancing T cell infiltration^85^. Whether the ECM barrier formed by mCAFs modulates marginalization of other immune cells or inhibits or promotes tumor cell invasion, remains to be explored.

The importance of immunomodulatory chemokines in cancer progression is undisputable. The expression of CXCR2 ligands, CXCL1-3 and CXCL5-8 by melanoma cells has been shown to control the immune cell composition of the TME, to contribute to the ability to escape tumor immune surveillance, to induce angiogenesis or to define the preferred sites of melanoma metastases^86–89^. In the present study, receptor-ligand analysis revealed that fibroblasts are the major source for cytokines and chemokines (Fig. 6A, B) and not the cancer cells themselves, thus highlighting the importance of fibroblasts in immune cell recruitment and cancer immune surveillance.

Intriguingly, while *CXCL2* was expressed by fibroblasts from all skin cancer types, melanoma-derived CAFs expressed high levels of *CXCL1-3, 5, 6* and *8* and *IL1B* as well as *IL6*, whereas the expression of *CXCL9-11* and *13* was high in non-melanoma CAFs (Fig. 6A). *LGALS9*, which has been shown to interact with *CD40* on T cells thereby attenuating their expansion and effector function, was strongly expressed in CAFs from BCC and SCC but not melanoma. Furthermore, HLA genes were highly expressed in CAFs but not normal fibroblasts (Fig. S8B), suggesting a role for CAFs as antigen-presenting cells. CAF-mediated cross-presentation of neo-antigens may directly suppress T cell function^90^. These findings indicate that although iCAFs are present in melanoma and non-melanoma skin cancers, the expression of chemokines and possibly other immunomodulating genes is tumor type-dependent. This is also reflected by the differentially regulated expression of TFs in iCAFs derived from melanoma and cutaneous SCCs (Fig. 3C). We substantiated the presence of differential crosstalk among tumor types and stages by testing the effect of conditioned media from various cancer cell lines on healthy skin-derived fibroblasts. Indeed, conditioned medium from metastasis-derived melanoma cell lines induced an iCAF-like phenotype and cytokine/chemokine expression, while conditioned medium of a primary melanoma cell line did not change the cytokine/chemokine expression (Fig. 6D). Furthermore, VM15 conditioned media, which consistently enhanced cytokine transcript levels, also stimulated T cell proliferation and activation (Figs. 6D, 7B,C). Surprisingly, the secretome of the subcutaneous metastasis-derived cell line VM26^76^ did not induce the expression of the majority of the tested cytokines and chemokines except for *CCL2*, which may be linked to different mutations. However, VM26-derived conditioned medium was still capable of activating T cells, which is not surprising as the cytokines not induced by VM26 shown in Fig. 6D are CXCR1/2 ligands that are known to recruit innate immune cells but not T cells. Interestingly, while the secretome of melanoma and SCC cell lines was capable of inducing an iCAF phenotype, induction of a mCAF phenotype could not be achieved in vitro. Thus, further investigations are necessary to define which signals or culture conditions prime fibroblasts towards mCAF differentiation in vitro and in situ. Likewise it remains elusive whether soluble CAF-derived factors or direct cell contact are essential for T cell activation. At first glance, the observation that iCAFs are more abundant in late-stage skin cancers may seem paradoxical, given their demonstrated ability to activate T cells in vitro. However, it is important to emphasize that the T cell activation assays offer only an initial indication of the interaction between iCAFs and T cells. The tumor microenvironment in advanced cancer stages is typically characterized by immune evasion and dysfunction, including T cell exhaustion — a state where T cells lose their effectiveness in responding to tumors. It remains to be determined whether iCAFs, while capable of stimulating initial T cell activation, may also contribute to the induction of T cell exhaustion or influence the function of other immune cells, such as macrophages or regulatory T cells, which could potentially support tumor progression. Future investigations are needed to clarify the dual role iCAFs may play in shaping the immune landscape within tumors, particularly in balancing immune activation and suppression in advanced disease stages.

In addition to the fibroblast heterogeneity in skin tumors, our data highlight the tremendous effect of the TME on all cells within a tumor. For example, melanocytes from BCC and SCC samples (tMC), which are part of the non-neoplastic cells in these tumor types, cluster separately from melanocytes that were derived from hMC (Figs. 2B and S2C). This indicates that the altered gene expression profile is likely induced by the TME. Further, our CNV analysis clearly shows that samples from the same tumor subtype (Melanoma I–III: Acral lentiginous melanoma, ALM) and body location can greatly differ at molecular level, which explains donor-specific clustering.

In summary, our work provides a cellular and molecular atlas of the three most frequent skin cancer types comprising neoplastic epithelial, mesenchymal, and immune cells. We further reveal and characterize three distinct CAF subsets and show that their abundance and associated signaling molecules and structural proteins critically impact the TME. Therefore, determining the predominant CAF subset within tumor samples may improve future diagnostic strategies and thereby open new avenues for better personalized therapies. Moreover, pharmacologically targeting CAFs to reduce ECM density could enhance T cell trafficking into tumor nests and, thus, the efficacy of checkpoint-inhibition therapy as well as the penetration of agents that directly target cancer cells.

## Methods

### Ethical approval

The present study complies with all relevant ethical regulations at the Medical University of Vienna and was approved by the Institutional Review Board under the ethical permits EK#1695/2021, EK#1783/2020 and EK#1555/2016. Written informed patient consent was obtained before tissue and blood collection in accordance with the Declaration of Helsinki. Consent to publish clinical information potentially identifying individuals was obtained and approved by the data-clearing committee of the Medical University of Vienna.

### Human healthy skin and tumor samples

Fresh 4 mm punch biopsies from central tumor and unaffected skin adjacent to tumors as well as 10 × 10 cm healthy skin samples from abdominal plastic surgeries were subjected to cell isolation procedure directly after surgery.

Healthy skin samples III-IV were cut into thin strips after removal of the fat layer. Epidermis was separated from dermis by Dispase 2 (1:100, Roche #04942078001, 20 mg/mL), digested in PBS at 37 °C for one hour before being peeled off. The epidermal sheet was minced and then subjected to enzymatic digestion in Trypsin-EDTA (GIBCO #25300-054) for 20 min at 37 °C in a shaking water bath (Epidermal sheet protocol for enrichment of keratinocytes). Healthy skin dermis and tumor samples were cut into tiny pieces and digested with Collagenase 1 (1:100, GIBCO #17100-017, 50 mg/mL), Collagenase 2 (1:100, GIBCO #17101-015, 50 mg/mL), Collagenase 4 (1:100, Sigma-Aldrich #C5138, 50 mg/mL), Hyaluronidase (1:100, Sigma-Aldrich #H3884, 10 mg/mL) and DNAseI (1:250, Sigma-Aldrich #DN25, 5 mg/mL) in DMEM/10%FCS for one hour in a 37 °C water bath (Protocol for enrichment of fibroblasts, keratinocytes and immune cells). After enzymatic digestion, the cell suspension was filtered and washed in PBS/10% FCS twice before subjecting it to FACS staining.

After Fc blocking (1:500, CD16/CD32 BD #553142, RRID:AB_394656), cell suspensions were stained for 30 min at 4 °C in the dark with CD45-BV605 (1:50, BioLegend #304042, RRID:AB_2562106), ITGA6-PeCy7 (1:100, BioLegend #313622, RRID:AB_2561705), CDH1-PeCy7 (1:200, BioLegend #147310, RRID:AB_2564188), FAP-APC (1:20, R&D Systems #FAB3715A, RRID:AB_2884010), CD90-AF700 (1:30, BioLegend #328120, RRID:AB_2203302) and CD31-FITC (1:30, BD Biosciences #563807), CD106-Pacific Blue (1:100, BD Biosciences #744309, RRID:AB_2742138), CD235ab-Pacific Blue (1:1000, BioLegend #306611, RRID:AB_2248153) and DAPI. ITGA6^+^/CDH1^+^ keratinocytes, FAP^+^/CD90^+^ fibroblasts, CD45^+^ immune cells and FAP^-^CD90^-^ double negative cells were single cell sorted directly into Smart-seq2 lysis buffer in 384-well plates (BD Aria Fusion). After sorting, plates were stored at −80 °C until they were sent for sequencing to the Eukaryotic Single Cell Genomics Facility (ESCG) at SciLifeLab at the Karolinska Institutet, Sweden.

### Immunohistochemistry

Immunohistochemistry was performed on 4 μm human FFPE sections according to standard protocols. Antigen retrieval was conducted in citrate buffer, pH 6.0, and 3%BSA/PBST was used for blocking. Primary antibodies against CD90 (THY1) (1:200, rabbit monoclonal [EPR3133], Abcam #ab133350, RRID:AB_11155503), FAP (1:200, rabbit monoclonal [D3V8A], Cell signaling #13801, RRID:AB_2798316), TAGLN (1:12.000, rabbit polyclonal, Thermo Scientific #PA5-27463, RRID:AB_2544939), DES (1:2000, rabbit monoclonal [Y266], Abcam #ab32362, RRID:AB_731901), CD31 (1:500, rabbit, Neomarkers, #RB10333-P1)), CD3 (1:200, rabbit, Abcam #ab16669, RRID:AB_443425) and COL11A1 (1:200, rabbit polyclonal, Abcam #ab64883, RRID:AB_1140613) were diluted in 1%BSA/PBST and incubated over night. A biotinylated goat anti-rabbit antibody (1:200, Vector BA-1000) was used as second step and incubated for 30 min at room temperature. Novocastra Streptavidin-HRP (Leica Biosystems Newcastle #RE7104) and Dako AEC+ High sensitivity substrate (Dako #K3469) were used for signal enhancement and development. For counterstaining, hematoxylin was used. Slides were scanned with Aperio (Leica Biosystems).

### RNAScope

RNAScope was conducted by using Multiplex Fluorescent Reagent Kit v2 from Advanced Cell Diagnostics, ACD Bio-Techne (#323135), according to the manufacturer’s protocol, with probes for *COL1A1* (#401891-C2), *COL11A1* (#400741-C3), *PTGDS* (#431471-C1), *MMP1* (#412641-C1), *RGS5* (#533421-C3) and *PDGFRA* (#604481-C1). For fluorescence staining, the TSA dyes Fluorescein, Cy3, and Cy5 (Akoyabio) and DAPI as nuclear stain were utilized. The Multiplex Fluorescent Reagent Kit v2 and the RNAscope® 4-Plex Ancillary kit were combined for 4-plex stainings against CXCL2 (#425251), CXCL8 (310381-C2), IL24 (404301-C3) and Col1A1 (401891-C4), or against MMP1 (#412641-C1), Col11A1 (#400741-C2), RGS5 (#533421-C3) and COL1A1 (#401891-C4). Opal™ fluorophores (Opal 520 or Opal780, Opal 570, Opal 620, and Opal 690) were utilized in the 4-plex staining’s. Images were captured by Vectra Polaris™ and image analysis was conducted with HALO® image analysis platform.

### Quantification of total CAFs, iCAFs, and mCAFs in tumor sections

To analyze and quantify the various CAF populations in tumor sections, we utilized the HALO® image analysis platform. The cell types included total CAFs (COL1A1^+^), iCAFs (COL1A1^+^MMP1^+^), and mCAFs (COL1A1^+^ COL11A1^+^ MMP1^−^), which were identified by specific marker combinations. Tumor sections from nodular (*n* = 8) and infiltrative BCC (*n* = 9), well (*n* = 8) and poorly (*n* = 10) differentiated SCC, as well as low (*n* = 8) and high (*n* = 9) grade melanoma were analyzed. A minimum of five representative regions of interest (ROIs) per sample were selected, excluding scarred or ulcerated areas to ensure accurate quantification. The HALO v3.6.4134 software with the HighPlex FL v4.2.14 plugin was employed for image analysis, with signal intensity thresholds set for each channel to differentiate positive from negative cells. Representative spatial plots were generated from analyzed sections in HALO®.

### T cell exclusion from tumor nests

BCC samples (*n* = 15) were stained for immunohistochemistry (IHC) with antibodies against CD3, followed by staining of consecutive sections using the RNAscope 4-Plex Multiplex Fluorescent Reagent Kit to *COL1A1*, *MMP1*, *Col11A1*, and *RGS5*. HALO® image analysis platform was used to perform image analysis and quantification. Regions of interest (ROI) were selected, where CD3^+^ cells were present in the stroma surrounding the tumor nests. A machine learning classifier, which was then trained to differentiate between tumor and stromal tissue, was applied to the ROIs. We quantified the number of CD3^+^ cells in the total tissue area or only in the tumor nests within the ROI. The sections were then co-registered with the RNAscope staining, and the numbers of CAFs (*COL1A1*^+^ cells) and matrix CAFs (*COL1A1*^+^*COL11A1*^+^*MMP1*^-^*RGS5*^-^) were quantified. The cell counts were then normalized to cells/mm².

### Fibroblast activation by conditioned medium of cancer cells: transcriptomic and proteomic analysis

Normal healthy dermal fibroblasts (NHDF) and fibroblasts from a primary melanoma were isolated as described above and cultured in DMEM containing 10% FBS and 50 µg/ml Gentamycin in a humidified incubator at 37 °C and 5% CO_2_.

#### Generation of conditioned medium (CM) from cancer cell lines

Melanoma cell lines (VM08, VM15, VM19, VM25, VM26)^76^ were cultured in RPMI1640 (GIBCO #11875093) containing 10% FBS (GIBCO #26140079), 2 mM L-glutamine (GIBCO #25030081) and 50 U/ml streptomycin/penicillin (GIBCO #15070063). SCC13^75^ cell line (RRID:CVCL_4029) was cultured in DMEM Glutamax containing 10% FBS, 2 mM L-glutamine (GIBCO #25030081), 50 U/ml streptomycin/penicillin (GIBCO #15070063), 5 µg/ml insulin and 10 µg/ml transferrin. When cells reached 70-80% confluency, they were washed with PBS, and DMEM/10% FBS was added. Conditioned medium (CM) was collected 48 hours later, centrifuged with 300 *g* for 10 minutes and stored at −20 °C.

#### Fibroblast activation assay

NHDF and cancer cells were seeded into 6 well plates for 24 hours. Then, medium of the NHDF was exchanged with CM derived from cancer cells or NHDF as a control. Cells were harvested 72 hours later. RNA was isolated with the Qiagen RNeasy Mini Kit (Qiagen #74106). RevertAid H Minus First Strand cDNA Synthesis Kit (Thermo Scientific #K1631) was used to prepare cDNA after a DNaseI digestion step (Thermo Scientific #EN0521). Taqman 2xUniversal PCR Master Mix (Applied Biosystems #4324018) and Taqman probes for GAPDH (Hs99999905), CXCL1 (Hs00236937_m1), CXCL2 (Hs00601975_m1), CXCL3 (Hs00171061_m1), CXCL5 (Hs01099660_g1), CXCL6 (Hs00605742_g1), CXCL8 (Hs00174103_m1), CCL2 (Hs00234140_m1) and IL24 (Hs01114274_m1), Lumican (Hs00929860_m1), Col11A1 (Hs01097664_m1), Col4A1 (Hs00266237_m1), LOXL2 (Hs00158757_m1), Fibromodulin (Hs05632658_s1) and Col12A1 (Hs00189184_m1) were used in the qPCR.

LEGENDplex Assay: For proteomic analysis cancer CM or control medium was removed from fibroblasts after 72 h incubation. Cells were washed in PBS and fresh DMEM/10% FCS was added for 48 h. Supernatants were collected, centrifuged to remove cell debris and subjected to protein analysis using LEGENDplex kits (Human Essential Immune Response #740930 and Human Proinflammatory Chemokine Panel #740985, BioLegend). Data analysis was done in GraphPad Prism version 8.0.0 for Windows, GraphPad Software, San Diego, California USA, www.graphpad.com.

### T-cell activation assay

#### Naïve T cell isolation

Human peripheral blood obtained from healthy individuals with informed consent at the Department of Dermatology, Medical University of Vienna, was collected in heparinized tubes and immediately processed by mixing 1:1 with PBS, then layered over Ficoll-Paque™ PLUS (Cytiva). After density gradient centrifugation at 500 × *g*, 20 °C, for 20 min, the PBMC layer was transferred, and washed with PBS, and CD4+ and CD8 + T cells were isolated using the human CD4+ and CD8 + T cell isolation kits (Miltenyi Biotec), according to the manufacturer’s protocol. The isolated CD4+ and CD8 + T cells were stained with a mix of antibodies for 20 min at 4 °C in the dark: CD4-FITC (1:400, [RPA-T4], BioLegend #300501, RRID:AB_314070), CD8-PE-Cy7 (1:100, [HIT8a] BD Biosciences Cat# 555635, RRID:AB_395997), CD25-APC (1:100, [BC96], BioLegend Cat# 302609 (also 302610), RRID:AB_314279), CD14-APC-Cy7 (1:100, [M5E2], BioLegend Cat# 301820 (also 301819), RRID:AB_493695), CD45RO-PacificBlue (1:100, [UCHL1], BioLegend Cat# 304215 (also 304216), RRID:AB_493658), CD127-PE (1:100, [A019D5], BioLegend Cat# 351340 (also 351303, 351304), RRID:AB_2564136), CD16-PerCP-Cy5.5 (1:500, [3G8], (BioLegend Cat# 302028 (also 302027), RRID:AB_893262)). Cells were washed with PBS and resuspended in RPMI1640 without phenol red (Gibco) containing a 1:1000 dilution of 7-AAD viability dye (BioLegend). Naïve CD4 or CD8 T cells were sorted with the BD FACSAria™ II Cell Sorter based on CD4 + CD127 + CD14-CD16-CD45RO-CD25- or CD8 + CD127 + CD14-CD16-CD45RO-CD25-. The collected T cells were stained with a proliferation dye (1:500, eBioscience, V450) for 8 min at 37 °C, followed by washing with RPMI1640 media (Gibco) containing 10% FBS, 1% penicillin/streptomycin (Gibco), and 2 mM L-glutamine (Gibco).

#### Experimental setup and FACS analysis

2000 fibroblasts/CAFs or melanoma cells were seeded into 96-well plates using DMEM or RPMI1640 media (Gibco), containing 10% FBS, 1% penicillin/streptomycin (Gibco), and 2 mM L-glutamine (Gibco), respectively. After 24 h, the medium of NHDFs was replaced with a conditioned medium derived from either NHDFs or cancer cells. Following 72 h incubation at 37 °C, cells were washed with PBS, and 50 µL of RPMI containing 10% FBS, 1% penicillin/streptomycin (Gibco), 2 mM L-glutamine and 12.5 µL/mL Immunocult CD3/CD28 T cell activator (Stemcell) was added. Then 50 µL of RPMI containing 40,000 T cells at a ratio of 1 (CD8) to 1.5 (CD4) were added. The cells were harvested by scraping after 24 h or 96 h and subjected to FACS analysis for the assessment of T cell proliferation (24 h) and expression of CD69 (24 h), CD45RO (96 h) and CD62L (96 h) on CD4 or CD8 T cells (negative of fibroblast markers CD90 and FAP). Cells were stained in PBS/10%FCS and incubated for 20 min at 4 °C in the dark: CD4-FITC (1:400, [RPA-T4], BioLegend #300501, RRID:AB_314070), CD8-PE-Cy7 (1:100, [HIT8a] BD Biosciences Cat# 555635, RRID:AB_395997), CD69-PE (1:100, [FN50], BioLegend Cat# 985202, RRID:AB_2924641), CD90-AF647 (1:100, [5E10], BioLegend Cat# 328115 (also 328116), RRID:AB_893439), FAP-APC (1:100, [427819], R&D Systems Cat# FAB3715A-025), CD45RO-PacificBlue (1:100, [UCHL1], BioLegend Cat# 304244 (also 304205, 304206), RRID:AB_2564160), CD62L- PerCP-Cy5.5 (1:100, [DREG56], Elabscience Cat# E-AB-F1051A). Following incubation, cells were washed and resuspended in PBS with Fixable Viability Dye eFluor™ 780 APC-Cy7 (eBioscience) for subsequent analysis using CytoFlex LX Flow Cytometer (Beckman Coulter).

### Single-cell RNA sequencing

scRNA-seq was conducted by the Eukaryotic Single Cell Genomics Facility (ESCG) at SciLifeLab, Sweden according to the Smart-seq2 protocol^91^. Demultiplexed reads were aligned to the human genome (hg19 assembly) and the ERCC spike-in reference using STAR v2.4.2a in two-pass alignment mode^92^. Uniquely aligned reads were transformed into reads per million kilobase (RPKM) using rpkmforgenes()^93^. RPKM values were summed up when several isoforms of a gene were detected.

### Single-cell RNA sequencing data processing

Low-quality cells were removed by using thresholds for RPKM values and number of genes expressed per cell. The lower threshold was referred to empty-well controls, the upper threshold was set based on unusually high RPKM-values of clearly visible outliers. We considered good quality when a minimum of 400 genes and RPKM-values between 150.000 and 8.000.000 per cell were reached. Finally, 4824 cells were retained after quality control.

Subsequent data analysis was carried out by using R3.6.2 and the Seurat package v3 (Stuart*, Butler*, et al., Cell 2019). RPKM values for each gene per cell were normalized and natural-log transformed (NormalizeData: normalization.method = ”Log-Normalize”). The 2000 most variable genes were identified (FindVariableGenes: selection.method = ‘vst’), the data scaled and principal component analysis (PCA) was performed. The first 20 principal components 1:20, resolution 0.2 and Seurat default parameters were used for UMAP generation of first-level clustering. Subsequently, clusters for second-level clustering were selected based on commonly known signature gene expression: Healthy keratinocytes, SCC and BCC (*KRT5*, *KRT14*), melanocytes and melanoma cells (*MLANA*), immune cells (*CD45*) as well as fibroblasts and vSMCs (*COL1A1*, *RERGL*).

Differentially expressed genes were identified by the Seurat function FindAllMarkers. For generation of UMAPs, violin and bar plots, ggplot2 v3.3.2 were used.

### Copy number variations for estimation of malignancy

*InferCNV* of the Trinity CTAT Project (https://github.com/broadinstitute/inferCNV) was used to calculate CNVs for healthy and malignant keratinocytes and separately for melanocytes and melanoma cells in comparison to stromal cells. For CreateInfercnvObject, healthy stromal cells (Healthy donor cells from Fibroblast&vSMC second level clustering, which includes fibroblasts, vSMCs, and pericytes) were used as reference for CNV estimation. *InferCNV* operations were performed by infercnv::run using min_cells_per_gene = 3, cutoff = 1, cluster_by_groups = T, denoise = T, HMM = T, analysis_mode = subclusters, hclust_method = ward.D2, tumor_subcluster_partition_method = random_trees. Estimation of malignancy was performed as previously described^27^, using a Pearson correlation cutoff of 0.45 or 0.40 and a sum of squares (SoS) cutoff of 0.017 or 0.026 for healthy and malignant keratinocytes or melanocytes and melanoma cells, respectively. In the CNV estimation plots for melanocytes and melanoma cells (Fig. S3B) we highlighted melanocytes derived from cluster hMC (melanocytes derived from healthy skin) and tMC (melanocytes derived from SCC, BCC and melanoma adjacent from unaffected skin samplesadjacent to melanoma) where most of the cells are nicely found in the lower quadrants as expected (CNV- and undefined).

CNV estimation based on RNA expression only detects genomic aberrations that affects larger chromosomal sections. Thus, we also analyzed the expression of certain genes in keratinocytes from BCC and SCC samples in comparison to healthy keratinocytes. For determining neoplastic keratinocytes in BCC samples we used PTCH1 and PTCH2^56,57^ (Fig. S2A, B).

### Trajectory analysis

We used Monocle2^66^ (v2.28, R4.0.0) and Monocle3^67^ (v0.2.1, R3.6.2) to perform trajectory analysis. For both methods, we extracted RPKM data, phenotype data, and feature data from the Seurat object (second-level clustering of fibroblasts without vSMC) from which we created a newCellDataSet(lowerDetectionLimit = 0.1, expressionFamily = tobit()) or a new_cell_data_set() object using default parameters.

For Monocle2, we converted our RPKM data into mRNA counts using relative2abs() and generated the NewCellDataSet(lowerDetectionLimit = 0.5, expressionFamily = negbinomial.size()) object again. As quality filtering and clustering were already performed in Seurat, we directly constructed single cell trajectories using all significantly (adjusted p-value < 0.01) regulated DEGs (FindMarkers()) as input parameters for ordering cells. For calculating pseudotime, we used healthy skin cells from controls as our starting point. Cells were plotted using plot_cell_trajectory() colored by “clusters”, “category” and “pseudotime”.

For Monocle 3, we manually added clusters, UMAP and PCA parameters to the new_cell_data_set() object and calculated the trajectory graph with learn_graph(object, use_partition = F). For calculating pseudotime we used healthy skin clusters (pFIB and rFIB) as root_cells and used plot_cells(color_cells_by = “pseudotime”) to present the data.

### Heatmaps

*ComplexHeatmap* v2.2.0 function was used to represent gene expression of single cells or mean gene expression per cluster in heatmaps as z-scores.

### Receptor–ligand analysis

For receptor-ligand pairing the previously published method developed by Simon Joost was used, but with additional adjustments for run-time and parallel computing^94^. Receptor-ligand interactions were analyzed between fibroblast clusters, immune cell clusters, neoplastic and healthy keratinocyte and melanocyte clusters.

A signature gene list, containing potential ligands and receptors of each cluster, was generated by the Seurat function FindMarkers() at the level of second-level clustering. Potential ligand-receptor interactions were identified by querying the combined receptor-ligand database from Ramilowski et al.^95^ and Cabello-Aguilar et al.^96^.

For each cluster pair, the number of identified receptor-ligand pairs was compared to the number of pairs obtained from an equally sized randomly sampled pool of receptors and ligands. This was repeated 10.000 times to test for significantly enriched interactions (*p* ≤ 0.05 for Benjamini–Hochberg-corrected *p*-values). An additional prerequisite for a valid receptor-ligand pairing was the presence of at least 2.5% cells of the same donor in both of the potentially interacting clusters (eg. iCAFs interacting with tCD4 requires at least 2.5% cells from the same donor in each of the clusters).

Used packages: *python* 3.7.6, *pandas* 1.0.1, *numpy* 1.18.1, *matplotlib* 3.2.2. Receptor–Ligand heatmaps were generated with *seaborn* 0.11.0 using mean z-scores per donor per cluster.

Additionally, we verified receptor-ligand interactions with CellChat^74^. The communication probability was calculated according to default parameters. We present selected receptor-ligand pairs as circular plots using the function netVisual individual(source.use = c(“mCAF”, “iCAF”), layout = chord).

### Module score

Module Scores were calculated by AddModuleScore() function from Seurat, and genes sets were represented as violin plots. Individual genes of a gene set are shown in heatmaps. Genes that showed absolutely no expression in any cluster were excluded from heatmaps and module score calculation (chemokines: CCL4, CCL14, CXCL7; cytokines: IL9, IL31; MMPs: MMP26). Statistical analysis was done by non-parametric Wilcox rank-sum test using ggplot2 function stat_compare_means().

### Melanoma scRNAseq validation dataset

For the melanoma validation dataset, pre-treatment samples (*n* = 5) were collected from stage IV melanoma patients as part of a trial investigating anti-CD20 treatment in a therapeutic setting (10.1038/s41467-017-00452-4). After biopsy of a lesion, single-cell suspensions were immediately frozen. Thawed suspensions were subjected to scRNA-seq using the Chromium Single Cell Controller and Single Cell 5’ Library & Gel Bead Kit v1.1 (10X Genomics, Pleasanton, CA) according to the manufacturer’s protocol. Sequencing was performed using the Illumina NovaSeq platform and the 150 bp paired-end configuration.

Preprocessing of the scRNA-seq data was performed using Cell Ranger version 6.1.2 (10x Genomics). Expression data was processed using R (version 4.2.1) and Seurat (version 4.0.5). Cells with less than 1,000 genes or more than 10% of relative mitochondrial gene counts were removed. The data was processed following Seurat’s scTransform workflow. Sample-specific batch effects were corrected using Harmony. Clusters were identified using Seurat’s “FindNeighbors” and “FindClusters” functions, using the first 32 dimensions of the Harmony-corrected embedding and a resolution of 1.5. Cell types were subsequently identified based on canonical markers. Heatmaps and module scores were generated as described above.

### Publicly available dataset

We reanalyzed the publicly available human HNSCC dataset (GSE103322) using R3.6.2 and the Seurat package v3^19^. As described by the authors, we regressed for the variable *processedbyMaximaenzyme*. The cell annotation, which was provided in the metadata file, was used to select the cells for clustering of the fibroblasts. Based on markers that were described by the authors, the clusters for CAFs, myofibroblasts and intermediate fibroblasts were assigned. A heatmap (ComplexHeatmap v2.2.0) was generated presenting gene expression as means of z-scores per cluster using the same genes as shown in the heatmap in Fig. S2C of the original publication, but extended it by commonly accepted pericyte and vSMC markers.

Additionally, we identified our marker genes for mCAFs, iCAFs, RGS5^+^ cells and healthy fibroblasts in the fibroblast population of the HNSCC dataset (GSE103322)^19^, cutaneous human SCC (GSE144240)^24^ and human invasive BCC (GSE181907)^29^ and represented it in heatmaps showing gene expression as means of z-scores.

For the human cutaneous SCC (GSE144240) dataset, fibroblast cell annotation was provided by the authors.

For the human invasive BCC dataset, cell annotations for the fibroblast subclusters (FC1-FC4), as described in the original paper^29^ were provided by the authors upon request. As some marker genes were expressed in very few cells, we used a cutoff of at least 2% of cells expressing the gene in order to include it into the heatmap.

For reanalysis of this datasets, we used R3.6.2, Seurat package v3 and *ComplexHeatmap* v2.2.0.

### Reporting summary

Further information on research design is available in  Nature Portfolio Reporting Summary linked to this article.

## Supplementary information

Supplementary Information

Reporting Summary

## Source data

Source Data

Transparent Peer Review file

## Data Availability

The scRNAseq data generated in this study have been deposited in the European Genome-Phenome Archive under accession code EGAS50000000365 and the GEO data under the accession code GSE254918. The raw data are available under restricted access for patient protection, access can be obtained by a request to EGA. Expression matrices are available at GEO. Additionally, we provide an online tool on our webpage (www.lichtenbergerlab.org) to explore our dataset. The human HNSCC, cSCC, and BCC publicly available datasets used in this study are available in the GEO database under accession code GSE103322, GSE144240, and GSE181907. The remaining data are available in the article, Supplementary Information and Source Data file. Source data are provided as a Source Data file. Source data are provided with this paper.

## References

[1] Gieniec, K. A., Butler, L. M., Worthley, D. L. & Woods, S. L. Cancer-associated fibroblasts—heroes or villains? *Br. J. Cancer* **121**, 293–302 (2019). 31289350 10.1038/s41416-019-0509-3 PMC6738083

[2] Kalluri, R. The biology and function of fibroblasts in cancer. *Nat. Rev. Cancer* **16**, 582–598 (2016). 27550820 10.1038/nrc.2016.73

[3] Li, T. et al. Hepatocellular carcinoma-associated fibroblasts trigger NK cell dysfunction via PGE2 and IDO. *Cancer Lett.* **318**, 154–161 (2012). 22182446 10.1016/j.canlet.2011.12.020

[4] Mariathasan, S. et al. TGFβ attenuates tumour response to PD-L1 blockade by contributing to exclusion of T cells. *Nature* **554**, 544–548 (2018). 29443960 10.1038/nature25501 PMC6028240

[5] Özdemir, B. C. et al. Depletion of carcinoma-associated fibroblasts and fibrosis induces immunosuppression and accelerates pancreas cancer with reduced survival. *Cancer Cell* **25**, 719–734 (2014). 24856586 10.1016/j.ccr.2014.04.005 PMC4180632

[6] Paulsson, J. & Micke, P. Prognostic relevance of cancer-associated fibroblasts in human cancer. *Semin. Cancer Biol.* **25**, 61–68 (2014). 24560651 10.1016/j.semcancer.2014.02.006

[7] Rhim, A. D. et al. Stromal elements act to restrain, rather than support, pancreatic ductal adenocarcinoma. *Cancer Cell* **25**, 735–747 (2014). 24856585 10.1016/j.ccr.2014.04.021 PMC4096698

[8] Lichtenberger, B. M. & Kasper, M. Cellular heterogeneity and microenvironmental control of skin cancer. *J. Intern. Med.* **289**, 614–628 (2021). 32976658 10.1111/joim.13177

[9] Mezawa, Y., & Orimo, A. Phenotypic heterogeneity, stability and plasticity in tumor‐promoting carcinoma‐associated fibroblasts. *FEBS J*. **289**, 2429–2447 (2021). 10.1111/febs.15851 33786982

[10] Philippeos, C. et al. Spatial and single-cell transcriptional profiling identifies functionally distinct human dermal fibroblast subpopulations. *J. Investig. Dermatol.* **138**, 811–825 (2018). 29391249 10.1016/j.jid.2018.01.016 PMC5869055

[11] Tabib, T., Morse, C., Wang, T., Chen, W. & Lafyatis, R. SFRP2/DPP4 and FMO1/LSP1 define major fibroblast populations in human skin. *J. Investig. Dermatol.* **138**, 802–810 (2018). 29080679 10.1016/j.jid.2017.09.045 PMC7444611

[12] Vorstandlechner, V. et al. Deciphering the functional heterogeneity of skin fibroblasts using single‐cell RNA sequencing. *FASEB j.* **34**, 3677–3692 (2020). 31930613 10.1096/fj.201902001RR

[13] Kieffer, Y. et al. Single-cell analysis reveals fibroblast clusters linked to immunotherapy resistance in cancer. *Cancer Discov.* **10**, 1330–1351 (2020). 32434947 10.1158/2159-8290.CD-19-1384

[14] Cords, L. et al. Cancer-associated fibroblast classification in single-cell and spatial proteomics data. *Nat. Commun.* **14**, 4294 (2023). 37463917 10.1038/s41467-023-39762-1 PMC10354071

[15] Norton, J., Foster, D., Chinta, M., Titan, A. & Longaker, M. Pancreatic cancer-associated fibroblasts (CAF): under-explored target for pancreatic cancer treatment. *Cancers* **12**, 1347 (2020). 32466266 10.3390/cancers12051347 PMC7281461

[16] Wang, Y. et al. Single-cell analysis of pancreatic ductal adenocarcinoma identifies a novel fibroblast subtype associated with poor prognosis but better immunotherapy response. *Cell Discov.* **7**, 36 (2021). 34035226 10.1038/s41421-021-00271-4 PMC8149399

[17] Khaliq, A. M. et al. Refining colorectal cancer classification and clinical stratification through a single-cell atlas. *Genome. Biol.* **23**, 113 (2022). 10.1186/s13059-022-02677-z PMC9092724 35538548

[18] Pelka, K. et al. Spatially organized multicellular immune hubs in human colorectal cancer. *Cell* **184**, 4734–4752.e20 (2021). 34450029 10.1016/j.cell.2021.08.003 PMC8772395

[19] Puram, S. V. et al. Single-cell transcriptomic analysis of primary and metastatic tumor ecosystems in head and neck cancer. *Cell* **171**, 1611–1624.e24 (2017). 29198524 10.1016/j.cell.2017.10.044 PMC5878932

[20] Guo, T., Li, W. & Cai, X. Applications of single-cell omics to dissect tumor microenvironment. *Front. Genet.* **11**, 548719 (2020). 33329692 10.3389/fgene.2020.548719 PMC7729000

[21] Ganier, C. et al. Multiscale spatial mapping of cell populations across anatomical sites in healthy human skin and basal cell carcinoma. *Proc. Natl Acad. Sci. USA* **121**, e2313326120 (2024). 38165934 10.1073/pnas.2313326120 PMC10786309

[22] Schütz, S. et al. Functionally distinct cancer-associated fibroblast subpopulations establish a tumor-promoting environment in squamous cell carcinoma. *Nat. Commun.* **14**, 5413 (2023). 37669956 10.1038/s41467-023-41141-9 PMC10480447

[23] Jerby-Arnon, L. et al. A cancer cell program promotes T cell exclusion and resistance to checkpoint blockade. *Cell* **175**, 984–997.e24 (2018). 30388455 10.1016/j.cell.2018.09.006 PMC6410377

[24] Ji, A. L. et al. Multimodal analysis of composition and spatial architecture in human squamous cell carcinoma. *Cell* **182**, 497–514.e22 (2020). 32579974 10.1016/j.cell.2020.05.039 PMC7391009

[25] Li, H. et al. Dysfunctional CD8 T cells form a proliferative, dynamically regulated compartment within human melanoma. *Cell* **176**, 775–789.e18 (2019). 30595452 10.1016/j.cell.2018.11.043 PMC7253294

[26] Sade-Feldman, M. et al. Defining T cell states associated with response to checkpoint immunotherapy in melanoma. *Cell* **175**, 998–1013.e20 (2018). 30388456 10.1016/j.cell.2018.10.038 PMC6641984

[27] Tirosh, I. et al. Dissecting the multicellular ecosystem of metastatic melanoma by single-cell RNA-seq. *Science* **352**, 189–196 (2016). 27124452 10.1126/science.aad0501 PMC4944528

[28] Yost, K. E. et al. Clonal replacement of tumor-specific T cells following PD-1 blockade. *Nat. Med.* **25**, 1251–1259 (2019). 31359002 10.1038/s41591-019-0522-3 PMC6689255

[29] Yerly, L. et al. Integrated multi-omics reveals cellular and molecular interactions governing the invasive niche of basal cell carcinoma. *Nat. Commun.* **13**, 4897 (2022). 35986012 10.1038/s41467-022-32670-w PMC9391376

[30] Gao, Y. et al. CD63 ^+^ cancer‐associated fibroblasts confer tamoxifen resistance to breast cancer cells through exosomal miR‐22. *Adv. Sci.* **7**, 2002518 (2020). 10.1002/advs.202002518 PMC7610308 33173749

[31] Ishibashi, M. et al. CD200-positive cancer-associated fibroblasts augment the sensitivity of epidermal growth factor receptor mutation-positive lung adenocarcinomas to EGFR Tyrosine kinase inhibitors. *Sci. Rep.* **7**, 46662 (2017). 28429795 10.1038/srep46662 PMC5399371

[32] Su, S. et al. CD10+GPR77+ cancer-associated fibroblasts promote cancer formation and chemoresistance by sustaining cancer stemness. *Cell* **172**, 841–856.e16 (2018). 29395328 10.1016/j.cell.2018.01.009

[33] Chen, Z. et al. Single-cell RNA sequencing highlights the role of inflammatory cancer-associated fibroblasts in bladder urothelial carcinoma. *Nat. Commun.* **11**, 5077 (2020). 33033240 10.1038/s41467-020-18916-5 PMC7545162

[34] Elyada, E. et al. Cross-species single-cell analysis of pancreatic ductal adenocarcinoma reveals antigen-presenting cancer-associated fibroblasts. *Cancer Discov.* **9**, 1102–1123 (2019). 31197017 10.1158/2159-8290.CD-19-0094 PMC6727976

[35] Geng, X. et al. Cancer-associated fibroblast (CAF) heterogeneity and targeting therapy of CAFs in pancreatic cancer. *Front. Cell Dev. Biol.* **9**, 655152 (2021). 34336821 10.3389/fcell.2021.655152 PMC8319605

[36] Öhlund, D. et al. Distinct populations of inflammatory fibroblasts and myofibroblasts in pancreatic cancer. *J. Exp. Med.* **214**, 579–596 (2017). 28232471 10.1084/jem.20162024 PMC5339682

[37] Sahai, E. et al. A framework for advancing our understanding of cancer-associated fibroblasts. *Nat. Rev. Cancer* **20**, 174–186 (2020). 31980749 10.1038/s41568-019-0238-1 PMC7046529

[38] Van Hove, L., & Hoste, E. Activation of fibroblasts in skin cancer. *J Investig. Dermatol.* **142**, 1026–1031 (2021). 10.1016/j.jid.2021.09.010 34600919

[39] Driskell, R. R. et al. Distinct fibroblast lineages determine dermal architecture in skin development and repair. *Nature* **504**, 277–281 (2013). 24336287 10.1038/nature12783 PMC3868929

[40] Jiang, D. et al. Two succeeding fibroblastic lineages drive dermal development and the transition from regeneration to scarring. *Nat. Cell Biol.* **20**, 422–431 (2018). 29593327 10.1038/s41556-018-0073-8

[41] Korosec, A. et al. Lineage identity and location within the dermis determine the function of papillary and reticular fibroblasts in human skin. *J. Investig. Dermatol.* **139**, 342–351 (2019). 30179601 10.1016/j.jid.2018.07.033

[42] Rinkevich, Y. et al. Identification and isolation of a dermal lineage with intrinsic fibrogenic potential. *Science* **348**, aaa2151 (2015). 25883361 10.1126/science.aaa2151 PMC5088503

[43] Omland, S. H. et al. Cancer associated fibroblasts (CAFs) are activated in cutaneous basal cell carcinoma and in the peritumoural skin. *BMC Cancer* **17**, 675 (2017). 28987144 10.1186/s12885-017-3663-0 PMC5806272

[44] Papaccio, F. et al. Profiling cancer-associated fibroblasts in melanoma. *IJMS* **22**, 7255 (2021). 34298873 10.3390/ijms22147255 PMC8306538

[45] Sasaki, K. et al. Analysis of cancer-associated fibroblasts and the epithelial-mesenchymal transition in cutaneous basal cell carcinoma, squamous cell carcinoma, and malignant melanoma. *Hum. Pathol.* **79**, 1–8 (2018). 29555579 10.1016/j.humpath.2018.03.006

[46] Stratigos, A. et al. Diagnosis and treatment of invasive squamous cell carcinoma of the skin: European consensus-based interdisciplinary guideline. *Eur. J. Cancer* **51**, 1989–2007 (2015). 26219687 10.1016/j.ejca.2015.06.110

[47] Waldman, A. & Schmults, C. Cutaneous squamous cell carcinoma. *Hematol./Oncol. Clin. North Am.* **33**, 1–12 (2019). 30497667 10.1016/j.hoc.2018.08.001

[48] Fernández-Figueras, M. T. et al. Position paper on a simplified histopathological classification of basal cell carcinoma: results of the European Consensus Project. *J. Eur. Acad. Dermatol. Venereol.* **36**, 351–359 (2022). 34931722 10.1111/jdv.17849

[49] Kim, D. P., Kus, K. J. B. & Ruiz, E. Basal cell carcinoma review. *Hematol./Oncol. Clin. North Am.* **33**, 13–24 (2019). 30497670 10.1016/j.hoc.2018.09.004

[50] Curti, B. D. & Faries, M. B. Recent advances in the treatment of melanoma. *N. Engl. J. Med.* **384**, 2229–2240 (2021). 34107182 10.1056/NEJMra2034861

[51] Davis, L. E., Shalin, S. C. & Tackett, A. J. Current state of melanoma diagnosis and treatment. *Cancer Biol. Ther.* **20**, 1366–1379 (2019). 31366280 10.1080/15384047.2019.1640032 PMC6804807

[52] Kurokawa, I., Takahashi, K., Moll, I. & Moll, R. Expression of keratins in cutaneous epithelial tumors and related disorders - distribution and clinical significance: Keratin expression in cutaneous epithelial tumors. *Exp. Dermatol.* **20**, 217–228 (2011). 21323743 10.1111/j.1600-0625.2009.01006.x

[53] Hutchin, M. E. et al. Sustained Hedgehog signaling is required for basal cell carcinoma proliferation and survival: conditional skin tumorigenesis recapitulates the hair growth cycle. *Genes Dev.* **19**, 214–223 (2005). 15625189 10.1101/gad.1258705 PMC545881

[54] Reynolds, G. et al. Developmental cell programs are co-opted in inflammatory skin disease. *Science* **371**, eaba6500 (2021). 33479125 10.1126/science.aba6500 PMC7611557

[55] Alexandrov, L. B. et al. Signatures of mutational processes in human cancer. *Nature* **500**, 415–421 (2013). 23945592 10.1038/nature12477 PMC3776390

[56] Zhang, H. et al. Role of PTCH and p53 genes in early-onset basal cell carcinoma. *Am. J. Pathol.* **158**, 381–385 (2001). 11159175 10.1016/S0002-9440(10)63980-6 PMC1850308

[57] Bale, A. E. The hedgehog pathway and basal cell carcinomas. *Hum. Mol. Genet.* **10**, 757–762 (2001). 11257109 10.1093/hmg/10.7.757

[58] Dotto, G. P. Multifocal epithelial tumors and field cancerization: stroma as a primary determinant. *J. Clin. Investig.* **124**, 1446–1453 (2014). 24691479 10.1172/JCI72589 PMC3973113

[59] Shimomura, Y. et al. APCDD1 is a novel Wnt inhibitor mutated in hereditary hypotrichosis simplex. *Nature* **464**, 1043–1047 (2010). 20393562 10.1038/nature08875 PMC3046868

[60] Janson, D. G., Saintigny, G., van Adrichem, A., Mahé, C. & El Ghalbzouri, A. Different gene expression patterns in human papillary and reticular fibroblasts. *J. Investig. Dermatol.* **132**, 2565–2572 (2012). 22696053 10.1038/jid.2012.192

[61] Hinz, B. The role of myofibroblasts in wound healing. *Curr. Res. Transl. Med.* **64**, 171–177 (2016). 27939455 10.1016/j.retram.2016.09.003

[62] Armulik, A., Genové, G. & Betsholtz, C. Pericytes: developmental, physiological, and pathological perspectives, problems, and promises. *Dev. Cell* **21**, 193–215 (2011). 21839917 10.1016/j.devcel.2011.07.001

[63] He, H. et al. Single-cell transcriptome analysis of human skin identifies novel fibroblast subpopulation and enrichment of immune subsets in atopic dermatitis. *J. Allergy Clin. Immunol.* **145**, 1615–1628 (2020). 32035984 10.1016/j.jaci.2020.01.042

[64] Paquet-Fifield, S. et al. A role for pericytes as microenvironmental regulators of human skin tissue regeneration. *J. Clin. Investig.* **119**, 2795–2806 (2009). 10.1172/JCI38535 PMC2735900 19652362

[65] Crisan, M. et al. A perivascular origin for mesenchymal stem cells in multiple human organs. *Cell Stem Cell* **3**, 301–313 (2008). 18786417 10.1016/j.stem.2008.07.003

[66] Qiu, X. et al. Reversed graph embedding resolves complex single-cell trajectories. *Nat. Methods* **14**, 979–982 (2017). 28825705 10.1038/nmeth.4402 PMC5764547

[67] Cao, J. et al. The single-cell transcriptional landscape of mammalian organogenesis. *Nature* **566**, 496–502 (2019). 30787437 10.1038/s41586-019-0969-x PMC6434952

[68] Nurmik, M., Ullmann, P., Rodriguez, F., Haan, S. & Letellier, E. In search of definitions: cancer‐associated fibroblasts and their markers. *Int. J. Cancer* **146**, 895–905 (2020). 30734283 10.1002/ijc.32193 PMC6972582

[69] Bartoschek, M. et al. Spatially and functionally distinct subclasses of breast cancer-associated fibroblasts revealed by single-cell RNA sequencing. *Nat. Commun.* **9**, 5150 (2018). 30514914 10.1038/s41467-018-07582-3 PMC6279758

[70] Hughes, S. & Chan-Ling, T. Characterization of smooth muscle cell and pericyte differentiation in the rat retina in vivo. *Invest. Ophthalmol. Vis. Sci.* **45**, 2795 (2004). 15277506 10.1167/iovs.03-1312

[71] Stapor, P. C., Sweat, R. S., Dashti, D. C., Betancourt, A. M. & Murfee, W. L. Pericyte dynamics during angiogenesis: new insights from new identities. *J. Vasc. Res.* **51**, 163–174 (2014). 24853910 10.1159/000362276 PMC4149862

[72] Dominguez, C. X. et al. Single-Cell rna sequencing reveals stromal evolution into LRRC15 ^+^ myofibroblasts as a determinant of patient response to cancer immunotherapy. *Cancer Discov.* **10**, 232–253 (2020). 31699795 10.1158/2159-8290.CD-19-0644

[73] Grout, J. A. et al. Spatial positioning and matrix programs of cancer-associated fibroblasts promote T-cell exclusion in human lung tumors. *Cancer Discov.* **12**, 2606–2625 (2022). 36027053 10.1158/2159-8290.CD-21-1714 PMC9633420

[74] Jin, S. et al. Inference and analysis of cell-cell communication using CellChat. *Nat. Commun.* **12**, 1088 (2021). 33597522 10.1038/s41467-021-21246-9 PMC7889871

[75] Lefort, K. et al. Notch1 is a p53 target gene involved in human keratinocyte tumor suppression through negative regulation of ROCK1/2 and MRCKα kinases. *Genes Dev.* **21**, 562–577 (2007). 17344417 10.1101/gad.1484707 PMC1820898

[76] Berger, W., Hauptmann, E., Elbling, L., Vetterlein, M., Kokoschka, E. M. & Micksche, M. Possible role of the multidrug resistance-associated protein (MRP) in chemoresistance of human melanoma cells. *Int. J. Cancer* **71**, 108–115 (1997). 9096673 10.1002/(sici)1097-0215(19970328)71:1<108::aid-ijc18>3.0.co;2-e

[77] Ping, Q. et al. Cancer-associated fibroblasts: overview, progress, challenges, and directions. *Cancer Gene Ther.* **28**, 984–999 (2021). 33712707 10.1038/s41417-021-00318-4

[78] Hosaka, K. et al. Pericyte–fibroblast transition promotes tumor growth and metastasis. *Proc. Natl Acad. Sci. USA* **113**, E5618–E5627 (2016). 27608497 10.1073/pnas.1608384113 PMC5035870

[79] Luo, H. et al. Pan-cancer single-cell analysis reveals the heterogeneity and plasticity of cancer-associated fibroblasts in the tumor microenvironment. *Nat. Commun.* **13**, 6619 (2022). 36333338 10.1038/s41467-022-34395-2 PMC9636408

[80] Foster, D. S., Jones, R. E., Ransom, R. C., Longaker, M. T. & Norton, J. A. The evolving relationship of wound healing and tumor stroma. *JCI Insight* **3**, e99911 (2018). 30232274 10.1172/jci.insight.99911 PMC6237224

[81] Otranto, M., Sarrazy, V., Bonté, F., Hinz, B., Gabbiani, G. & Desmouliere, A. The role of the myofibroblast in tumor stroma remodeling. *Cell Adhes. Migr.* **6**, 203–219 (2012). 10.4161/cam.20377 PMC3427235 22568985

[82] Brown, F. D. & Turley, S. J. Fibroblastic reticular cells: organization and regulation of the T lymphocyte life cycle. *J. Immunol.* **194**, 1389–1394 (2015). 25663676 10.4049/jimmunol.1402520 PMC4324549

[83] Koppensteiner, L., Mathieson, L., O’Connor, R. A. & Akram, A. R. Cancer-associated fibroblasts—an impediment to effective anti-cancer T cell immunity. *Front. Immunol.* **13**, 887380 (2022). 35479076 10.3389/fimmu.2022.887380 PMC9035846

[84] Ford, K. et al. NOX4 inhibition potentiates immunotherapy by overcoming cancer-associated fibroblast-mediated CD8 T-cell exclusion from tumors. *Cancer Res.* **80**, 1846–1860 (2020). 32122909 10.1158/0008-5472.CAN-19-3158 PMC7611230

[85] Ziani, L., Chouaib, S. & Thiery, J. Alteration of the antitumor immune response by cancer-associated fibroblasts. *Front. Immunol.* **9**, 414 (2018). 29545811 10.3389/fimmu.2018.00414 PMC5837994

[86] Dhawan, P., & Richmond, A. Role of CXCL1 in tumorigenesis of melanoma. *J. Leukoc Biol.* **72**, 9–18 (2002). PMC2668262 12101257

[87] Forsthuber, A. et al. CXCL5 as regulator of neutrophil function in cutaneous melanoma. *J. Investig. Dermatol.* **139**, 186–194 (2019). 30009831 10.1016/j.jid.2018.07.006

[88] Payne, A. S. & Cornelius, L. A. The role of chemokines in melanoma tumor growth and metastasis. *J. Investig. Dermatol.* **118**, 915–922 (2002). 12060384 10.1046/j.1523-1747.2002.01725.x

[89] Soler-Cardona, A. et al. CXCL5 facilitates melanoma cell–neutrophil interaction and lymph node metastasis. *J. Investig. Dermatol.* **138**, 1627–1635 (2018). 29474942 10.1016/j.jid.2018.01.035

[90] Harryvan, T. J. et al. Enhanced antigen cross-presentation in human colorectal cancer-associated fibroblasts through upregulation of the lysosomal protease cathepsin S. *J. Immunother. Cancer* **10**, e003591 (2022). 35264435 10.1136/jitc-2021-003591 PMC8915372

[91] Picelli, S., Björklund, Å. K., Faridani, O. R., Sagasser, S., Winberg, G. & Sandberg, R. Smart-seq2 for sensitive full-length transcriptome profiling in single cells. *Nat. Methods* **10**, 1096–1098 (2013). 24056875 10.1038/nmeth.2639

[92] Dobin, A. & Gingeras, T. R. Mapping RNA-seq reads with STAR. *Curr. Protoc. Bioinform.* **51**, 11.14.1–11.14.19 (2015). 10.1002/0471250953.bi1114s51 PMC4631051 26334920

[93] Ramsköld, D. et al. Full-length mRNA-Seq from single-cell levels of RNA and individual circulating tumor cells. *Nat. Biotechnol.* **30**, 777–782 (2012). 22820318 10.1038/nbt.2282 PMC3467340

[94] Joost, S. et al. Single-cell transcriptomics of traced epidermal and hair follicle stem cells reveals rapid adaptations during wound healing. *Cell Rep.* **25**, 585–597.e7 (2018). 30332640 10.1016/j.celrep.2018.09.059

[95] Ramilowski, J. A. et al. A draft network of ligand–receptor-mediated multicellular signalling in human. *Nat. Commun.* **6**, 7866 (2015). 26198319 10.1038/ncomms8866 PMC4525178

[96] Cabello-Aguilar, S. et al. SingleCellSignalR: inference of intercellular networks from single-cell transcriptomics. *Nucleic Acids Res.* **48**, e55 (2020). 32196115 10.1093/nar/gkaa183 PMC7261168

