# Supplementary material for: Cancer-associated fibroblast subtypes modulate the tumor-immune microenvironment and are associated with skin cancer malignancy
Source: Nat Commun. 2024 Nov 8;15:9678. doi: 10.1038/s41467-024-53908-9 (PMC11549091; doi:10.1038/s41467-024-53908-9)
Supplement: Supplementary file 1 — Supplementary Information [file 41467_2024_53908_MOESM1_ESM.pdf]

SUPPLEMENTARY INFORMATION

Cancer associated fibroblast subtypes modulate the tumor-immune microenvironment and are associated with skin cancer malignancy

Agnes Forsthuber[1,*], Bertram Aschenbrenner[1,*], Ana Korosec[1,*], Tina Jacob[2] , Karl Annusver[2], Natalia Krajic[1], Daria Kholodniuk[1], Sophie Frech[1], Shaohua Zhu[1], Kim Purkhauser[1], Katharina Lipp[1], Franziska Werner[3], Vy Nguyen[3], Johannes Griss[3], Wolfgang Bauer[3], Ana Soler Cardona[3], Benedikt Weber[1], Wolfgang Weninger[3], Bernhard Gesslbauer[4], Clement Staud[4], Jakob Nedomansky[4], Christine Radtke[4], Stephan N. Wagner[3], Peter Petzelbauer[1], Maria Kasper[2,#] and Beate M. Lichtenberger[1,#]

Affiliations:

[1]Skin and Endothelium Research Division, Department of Dermatology, Medical University of Vienna, Austria

[2]Department of Cell and Molecular Biology, Karolinska Institutet, Sweden

[3]Department of Dermatology, Medical University of Vienna, Austria

[4]Department of Plastic, Reconstructive and Aesthetic Surgery, Medical University of Vienna, Austria

* These authors contributed equally

# These authors jointly supervised this work

Corresponding authors:
beate.lichtenberger@meduniwien.ac.at
maria.kasper@ki.se

SUPPLEMENTAL TABLES AND FIGURES:

Table S1. Published skin cancer scRNA-seq datasets including fibroblasts/CAFs

Table S2. Details of donor samples

Figure S1. Gating scheme and quality control of sequencing data. Related to Figure 1

Figure S2. Estimation of malignant cells and additional second-level clustering information. Related to Figure 2

Figure S3. Estimation of malignant cells by Pearson correlation and sum of squares (SoS) of CNVs according to Tirosh et al., 2016. Related to Figure 2.

Figure S4. Characterization of the fibroblasts and vSMC cluster. Related to Figure 3.

Figure S5. Endothelial and vCAF markers in the *RGS5*$^{+}$ cluster. Related to Figure 4.

Figure S6. RNAScope staining for mCAFs and iCAFs. Related to Figure 5.

Figure S7. Spatial analysis of CAF subtypes *in situ*. Related to Figure 4, 5 and 6.

Figure S8. Matrix-associated, MMP and immunomodulatory gene panels characterize mCAFs and iCAFs. Related to Figures 5 and 6.

Figure S9. Receptor-Ligand analysis of mCAFs. Related to Figure 5.

Figure S10. Expression of ECM genes is not upregulated in NHDFs upon treatment with tumor CM. Related to Figure 6.

Figure S11. Protein expression in supernatants of fibroblasts upon stimulation with cancer cell-derived CM. Related to Figure 6.

Figure S12. Fibroblast-mediated late activation of CD4 and CD8 T cells. Related to Figure 7.

Figure S13. Reanalysis of published datasets reveals CAF subsets with gene signatures similar to mCAFs, iCAFs and RGS5^{+} cells in human oral and cutaneous SCC as well as invasive BCC.

SUPPLEMENTAL TABLES:

Table S1. Published skin cancer scRNA-seq datasets comprising fibroblasts

Manuscript	cancer type	tumor specimen	healthy specimen	total cells	CAFs	additional information
Tirosh et al., Science 2016 [1]	melanoma	19		4,645	61	
Jerby-Arnon et al., Cell 2018 [2]	melanoma	33		7,186	106	
Ji et al., Cell 2020 [3]	SCC	10	10 (same patients)	48,000	882	>80% of CAFs from 1 donor
Guerrero-Juarez et al., Science Advances 2022 [4]	BCC	4		36,392	5,775	CAFs only from 2 donors
Yerly et al., Nature Communications 2022 [5]	BCC	5		28,819	809	
Ganier et al., PNAS 2023 [6]	BCC	8	14	134,839	11,732	
Schütz et al., Nature Communications 2023 [7]	SCC	8+3(AK*)	3	115,053	16,648	

***Actinic Keratosis**

Table S2. Details of donor samples

Donors	Sample type	Body Part	Sex	Age at sampling (yrs)	Histology
BCC I	BCC	capillitium	male	47	Nodular basal cell carcinoma
BCC II	BCC	nose	male	80	Nodular basal cell carcinoma
BCC III	BCC	shoulder	male	77	Nodular basal cell carcinoma
Mel I	Melanoma	heel	male	78	Acral lentiginous melanoma, T4b, thickness 12 mm
Mel II	Melanoma	toe	male	86	Acral lentiginous melanoma, T4b, thickness 8 mm
Mel III	Melanoma	toe	female	87	Acral lentiginous melanoma, T4b, thickness 5 mm
SCC I	SCC	capillitium	male	56	Reoccurrence of poorly differentiated SCC in scar, thickness 3 mm
SCC II	SCC	lower leg	male	78	Well differentiated SCC, thickness 2 mm
SCC III	SCC	capillitium	male	93	Poorly differentiated SCC, thickness 4 mm
SCC IV	SCC	lower leg	male	92	SCC arisen from Bowens disease thickness 8 mm
Healthy I	Healthy skin	upper arm	female	63	
Healthy II	Healthy skin	abdomen	female	43	
Healthy III	Healthy skin	abdomen	female	59	
Healthy IV	Healthy skin	upper arm	female	44	
Healthy V	Healthy skin	abdomen	male	48	

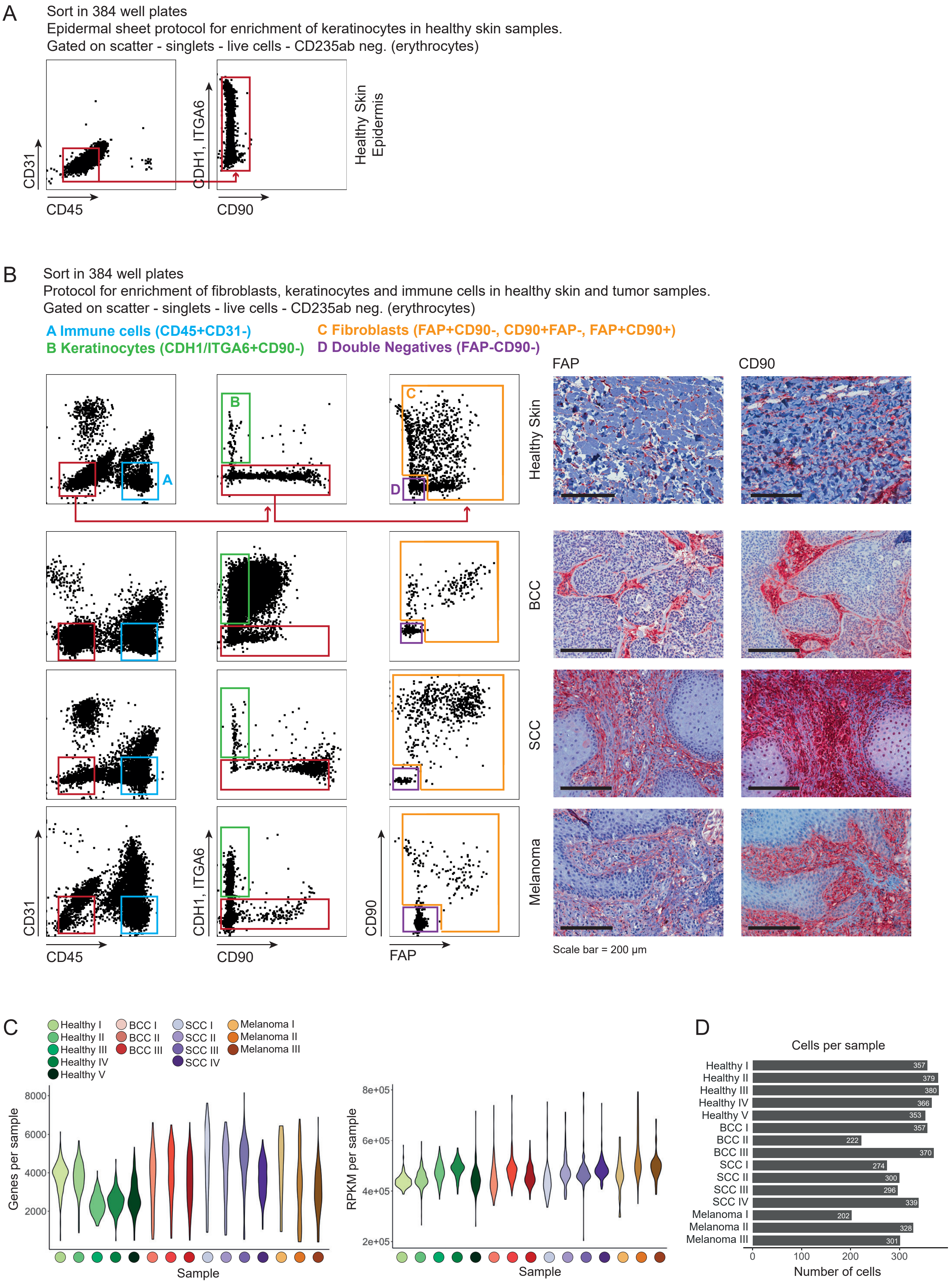
A
Sort in 384 well plates
Epidermal sheet protocol for enrichment of keratinocytes in healthy skin samples.
Gated on scatter - singlets - live cells - CD235ab neg. (erythrocytes)
CD31
CD45
CDH1, ITGA6
CD90
Healthy Skin
Epidermis
B
Sort in 384 well plates
Protocol for enrichment of fibroblasts, keratinocytes and immune cells in healthy skin and tumor samples.
Gated on scatter - singlets - live cells - CD235ab neg. (erythrocytes)
A Immune cells (CD45+CD31-)
B Keratinocytes (CDH1/ITGA6+CD90-)
C Fibroblasts (FAP+CD90-, CD90+FAP-, FAP+CD90+)
D Double Negatives (FAP-CD90-)
FAP
CD90
Healthy Skin
BCC
SCC
Melanoma
CD31
CD45
CDH1, ITGA6
CD90
CD90
FAP
Scale bar = 200 µm
C
Healthy I
Healthy II
Healthy III
Healthy IV
Healthy V
BCC I
BCC II
BCC III
SCC I
SCC II
SCC III
SCC IV
Melanoma I
Melanoma II
Melanoma III
Genes per sample
8000
6000
4000
2000
Sample
RPKM per sample
8e+05
6e+05
4e+05
2e+05
Sample
D
Cells per sample
Healthy I 357
Healthy II 379
Healthy III 380
Healthy IV 366
Healthy V 353
BCC I 357
BCC II 222
BCC III 370
SCC I 274
SCC II 300
SCC III 296
SCC IV 339
Melanoma I 202
Melanoma II 328
Melanoma III 301
0
100
200
300
Number of cells

Figure S1. Gating scheme and quality control of sequencing data. Related to Figure 1.
(A) Gating strategy for keratinocytes from healthy skin epidermal sheets (Healthy III - V).
(B) Gating strategy for fibroblasts, immune cells, double negatives and keratinocytes of BCC, SCC, melanoma and healthy skin dermis. Immunohistochemistry for FAP and CD90 in healthy skin dermis and tumor samples.
(C) Quality control of scRNA-seq data: Number of genes (left) and RPKM values (right) per sample after quality filtering (n=15).
(D) Number of cells per donor sample after quality filtering.

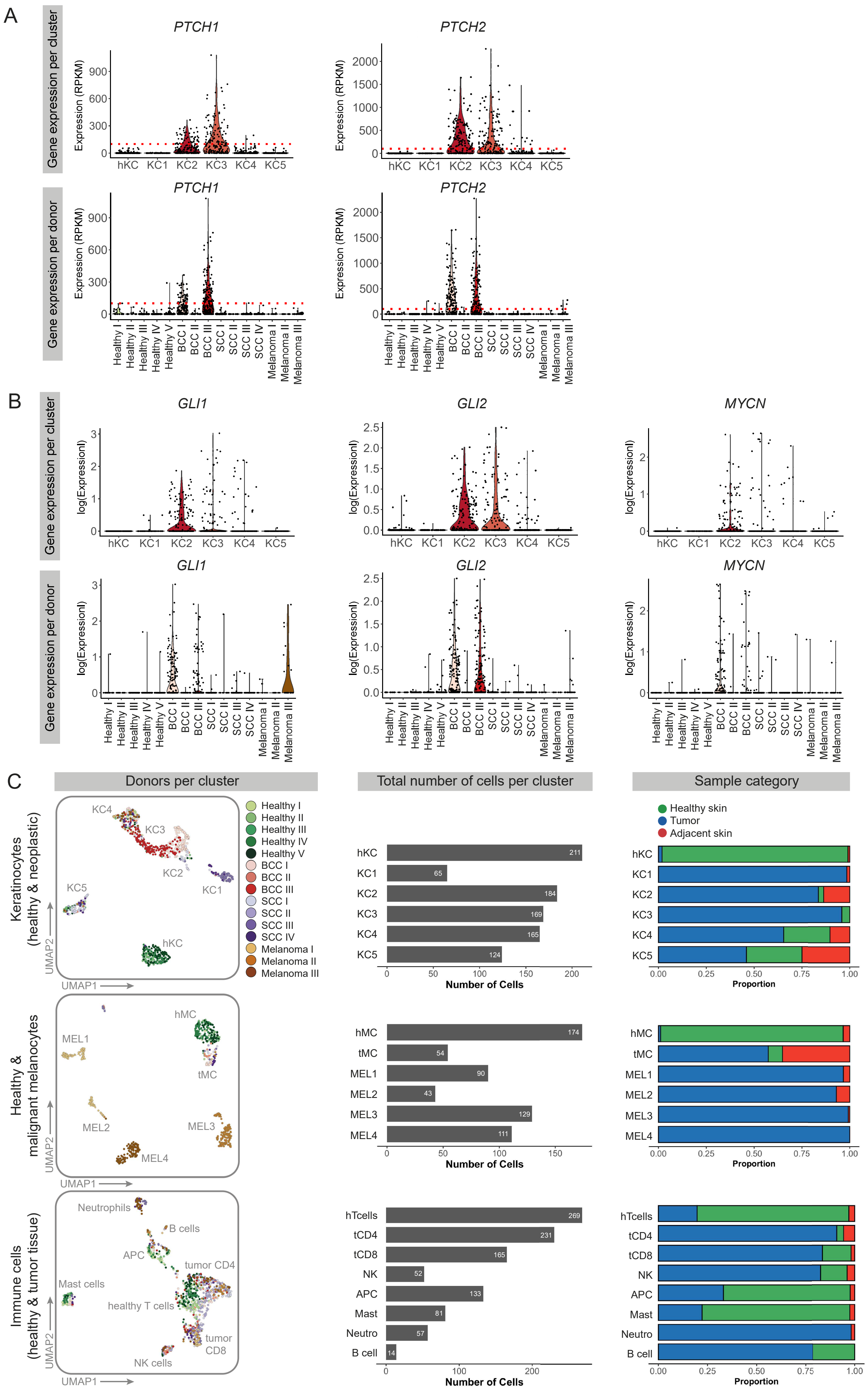
A
Gene expression per cluster
Gene expression per donor
PTCH1
PTCH2
Expression (RPKM)
hKC KC1 KC2 KC3 KC4 KC5
Healthy I Healthy II Healthy III Healthy IV Healthy V BCC I BCC II BCC III SCC I SCC II SCC III SCC IV Melanoma I Melanoma II Melanoma III
B
GLI1
GLI2
MYCN
log(Expression)
C
Donors per cluster
Total number of cells per cluster
Sample category
Keratinocytes (healthy & neoplastic)
Healthy & malignant melanocytes
Immune cells (healthy & tumor tissue)
UMAP1
UMAP2
Healthy I
Healthy II
Healthy III
Healthy IV
Healthy V
BCC I
BCC II
BCC III
SCC I
SCC II
SCC III
SCC IV
Melanoma I
Melanoma II
Melanoma III
Healthy skin
Tumor
Adjacent skin
hKC 211
KC1 65
KC2 184
KC3 169
KC4 165
KC5 124
hMC 174
tMC 54
MEL1 90
MEL2 43
MEL3 129
MEL4 111
hTcells 269
tCD4 231
tCD8 165
NK 52
APC 133
Mast 81
Neutro 57
B cell 14
Number of Cells
Proportion
Neutrophils
B cells
APC
tumor CD4
Mast cells
healthy T cells
tumor CD8
NK cells

Figure S2. Estimation of malignant cells and additional second-level clustering information. Related to Figure 2.

(A) *PTCH1* and *PTCH2* expression levels (RPKM) in healthy and malignant keratinocytes shown per cluster and per donor (n=15 donors). Threshold for overexpressing cells was adjusted to exclude healthy keratinocytes (*red dashed line: 100 RPKM*).

(B) *GLI1*, *GLI2* and *MYCN* expression levels in healthy and malignant keratinocytes shown per cluster and per donor (n=15 donors).

(C) UMAPs showing the distribution of donor samples (n=15 donors) per cluster, bar plot showing the total cell number and distribution of sample category per cluster.

A

Estimation of copy number variations (CNVs)
Healthy and malignant keratinocytes

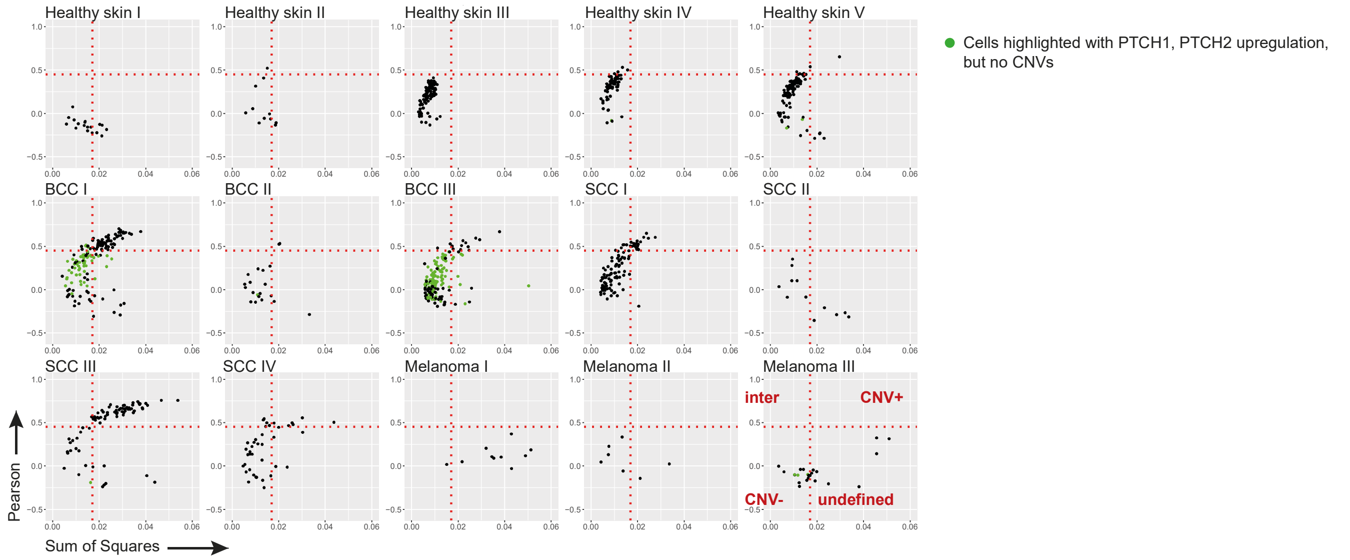

B

Estimation of copy number variations (CNVs)
Melanocytes and melanoma cells

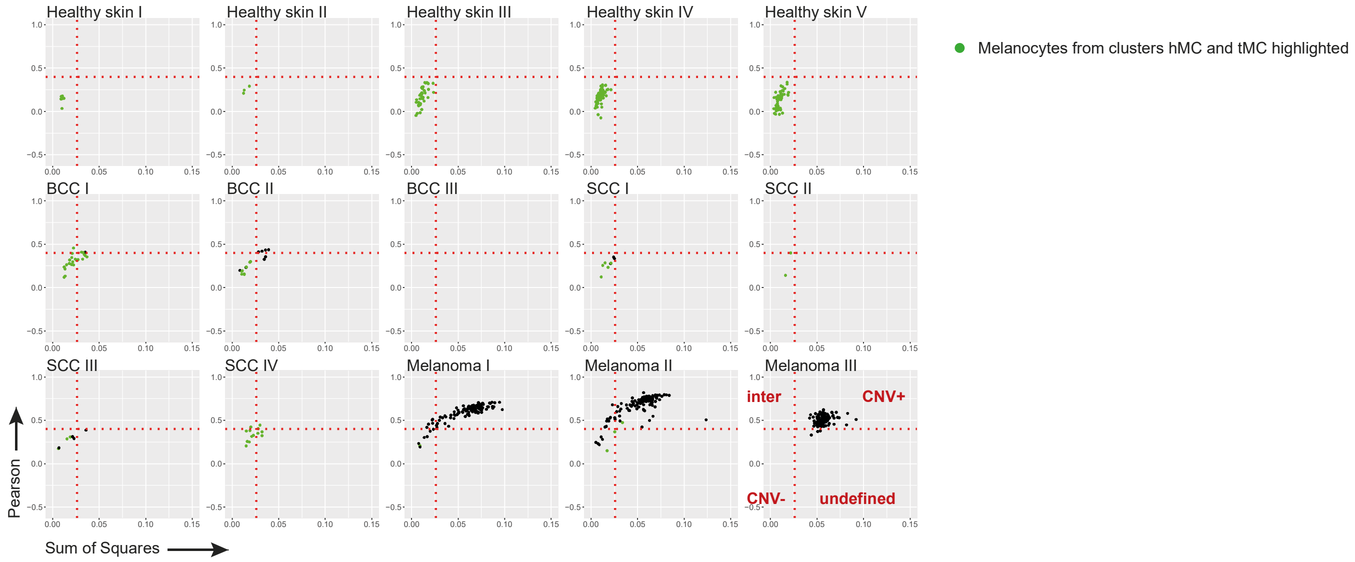

Figure S3. Estimation of malignant cells by Pearson correlation and sum of squares (SoS) of CNVs according to Tirosh et al., 2016[1]. Related to Figure 2.

(A) Malignancy estimation for healthy and malignant keratinocytes. Cells, which overexpress *PTCH1* and *PTCH2*, but do not show CNVs are highlighted in green.

(B) Malignancy estimation for melanocytes and melanoma cells. Cells from the healthy melanocyte cluster (hMC) and the tumor melanocyte cluster (tMC) are highlighted in green. Upper right quadrant shows CNV+ cells, upper left quadrant shows intermediate cells, lower left quadrant shows CNV- cells and lower right quadrant shows unclassifiable cells.

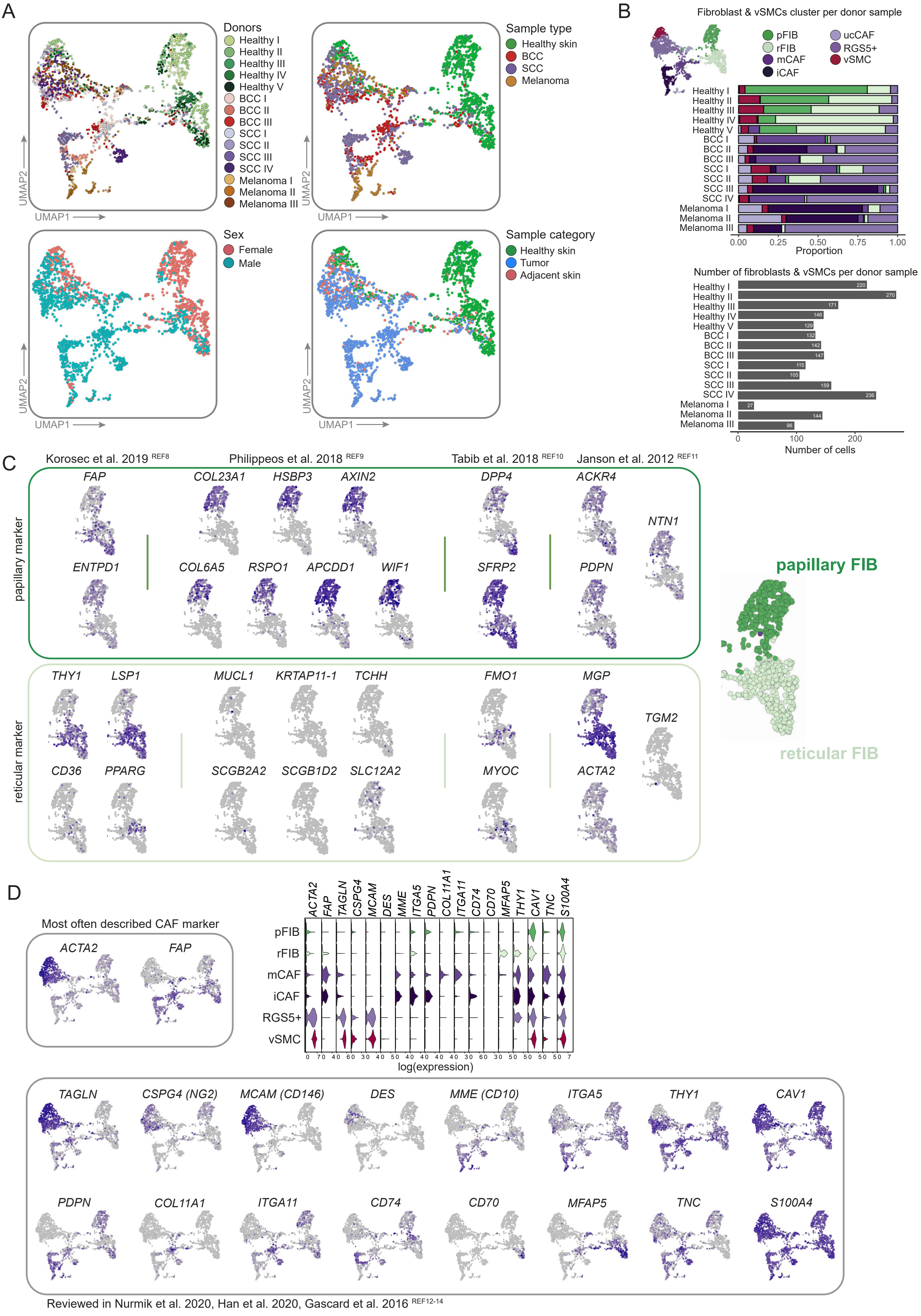
A
Donors
Healthy I
Healthy II
Healthy III
Healthy IV
Healthy V
BCC I
BCC II
BCC III
SCC I
SCC II
SCC III
SCC IV
Melanoma I
Melanoma II
Melanoma III
Sample type
Healthy skin
BCC
SCC
Melanoma
Sex
Female
Male
Sample category
Healthy skin
Tumor
Adjacent skin
UMAP1
UMAP2
B
Fibroblast & vSMCs cluster per donor sample
pFIB
rFIB
mCAF
iCAF
ucCAF
RGS5+
vSMC
Proportion
0.00
0.25
0.50
0.75
1.00
Number of fibroblasts & vSMCs per donor sample
Healthy I 220
Healthy II 270
Healthy III 171
Healthy IV 146
Healthy V 129
BCC I 132
BCC II 142
BCC III 147
SCC I 115
SCC II 105
SCC III 159
SCC IV 236
Melanoma I 27
Melanoma II 144
Melanoma III 96
0
100
200
Number of cells
C
Korosec et al. 2019 REF8
Philippeos et al. 2018 REF9
Tabib et al. 2018 REF10
Janson et al. 2012 REF11
papillary marker
FAP
COL23A1
HSBP3
AXIN2
DPP4
ACKR4
NTN1
ENTPD1
COL6A5
RSPO1
APCDD1
WIF1
SFRP2
PDPN
reticular marker
THY1
LSP1
MUCL1
KRTAP11-1
TCHH
FMO1
MGP
TGM2
CD36
PPARG
SCGB2A2
SCGB1D2
SLC12A2
MYOC
ACTA2
papillary FIB
reticular FIB
D
Most often described CAF marker
ACTA2
FAP
ACTA2
FAP
TAGLN
CSPG4
MCAM
DES
MME
ITGA5
PDPN
COL11A1
ITGA11
CD74
CD70
MFAP5
THY1
CAV1
TNC
S100A4
pFIB
rFIB
mCAF
iCAF
RGS5+
vSMC
log(expression)
TAGLN
CSPG4 (NG2)
MCAM (CD146)
DES
MME (CD10)
ITGA5
THY1
CAV1
PDPN
COL11A1
ITGA11
CD74
CD70
MFAP5
TNC
S100A4
Reviewed in Nurmik et al. 2020, Han et al. 2020, Gascard et al. 2016 REF12-14

Figure S4. Characterization of the fibroblasts and vSMC cluster. Related to Figure 3.
(A) UMAP showing the distribution of donors (n=15), sample type, sample category and sex on fibroblasts and vSMC second-level clustering.
(B) Bar plots showing the distribution of fibroblasts and vSMC clusters among donor samples (top) or number of fibroblasts and vSMC per donor samples (bottom).
(C) Commonly accepted papillary and reticular markers in the healthy fibroblast clusters pFib and rFib.
(D) Expression of previously described CAF markers on second-level clustering of fibroblasts and vSMCs.

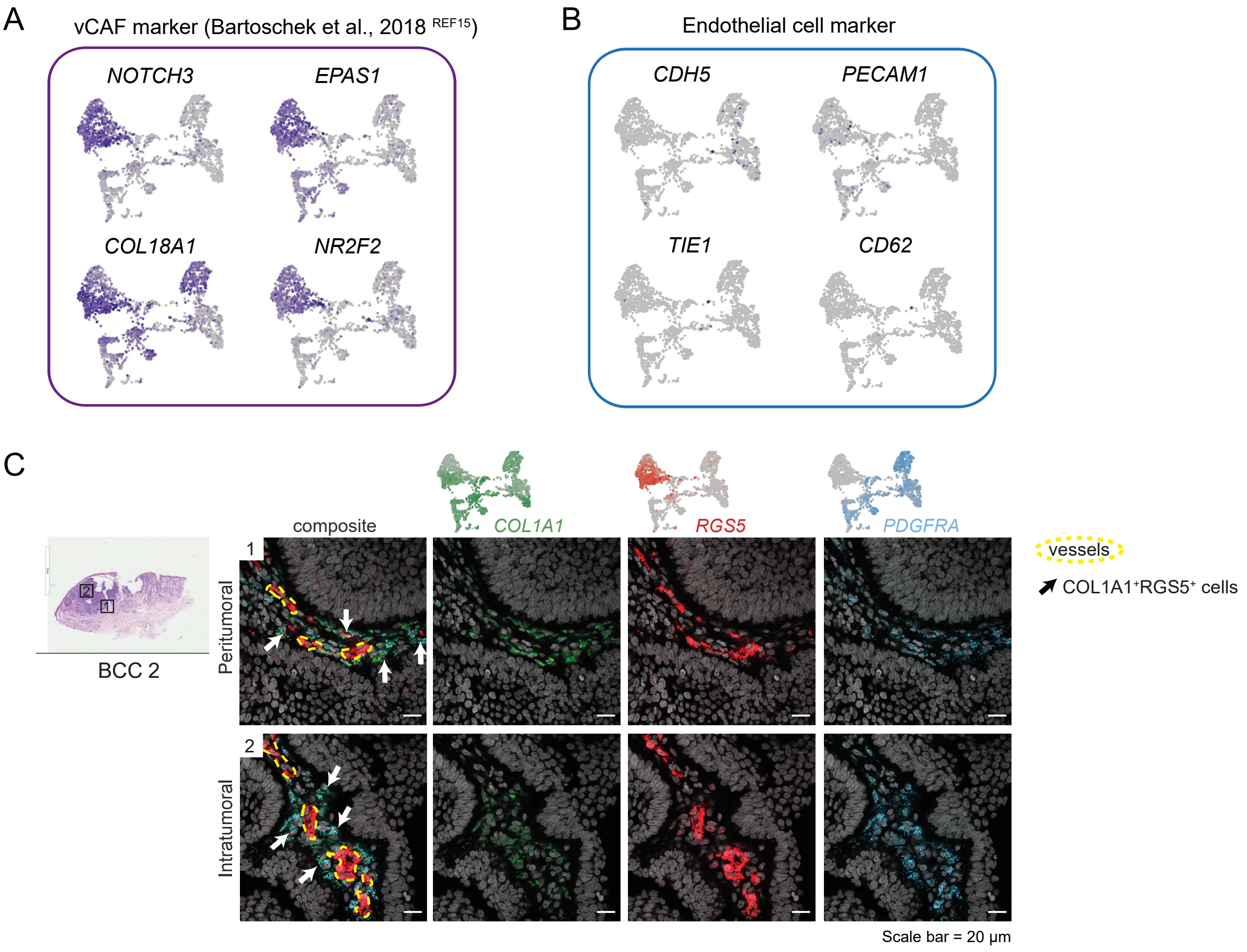
A
vCAF marker (Bartoschek et al., 2018 REF15)
NOTCH3
EPAS1
COL18A1
NR2F2
B
Endothelial cell marker
CDH5
PECAM1
TIE1
CD62
C
BCC 2
composite
COL1A1
RGS5
PDGFRA
vessels
COL1A1+RGS5+ cells
1
2
Peritumoral
Intratumoral
Scale bar = 20 μm

Figure S5. Endothelial and vCAF markers in the *RGS5*$^{+}$ cluster. Related to Figure 4.

(A) Previously described vCAF marker genes in a murine model for breast cancer are expressed in the *RGS5*$^{+}$ cluster.

(B) Absence of expression of endothelial cell marker genes in the *RGS5*$^{+}$ cluster.

(C) *COL1A1* (green), *RGS5* (red) and *PDGFRA* (blue) RNAScope fluorescence stainings in BCC II (n=10 biologically independent tumor samples). Scale bar represents 20 μm.

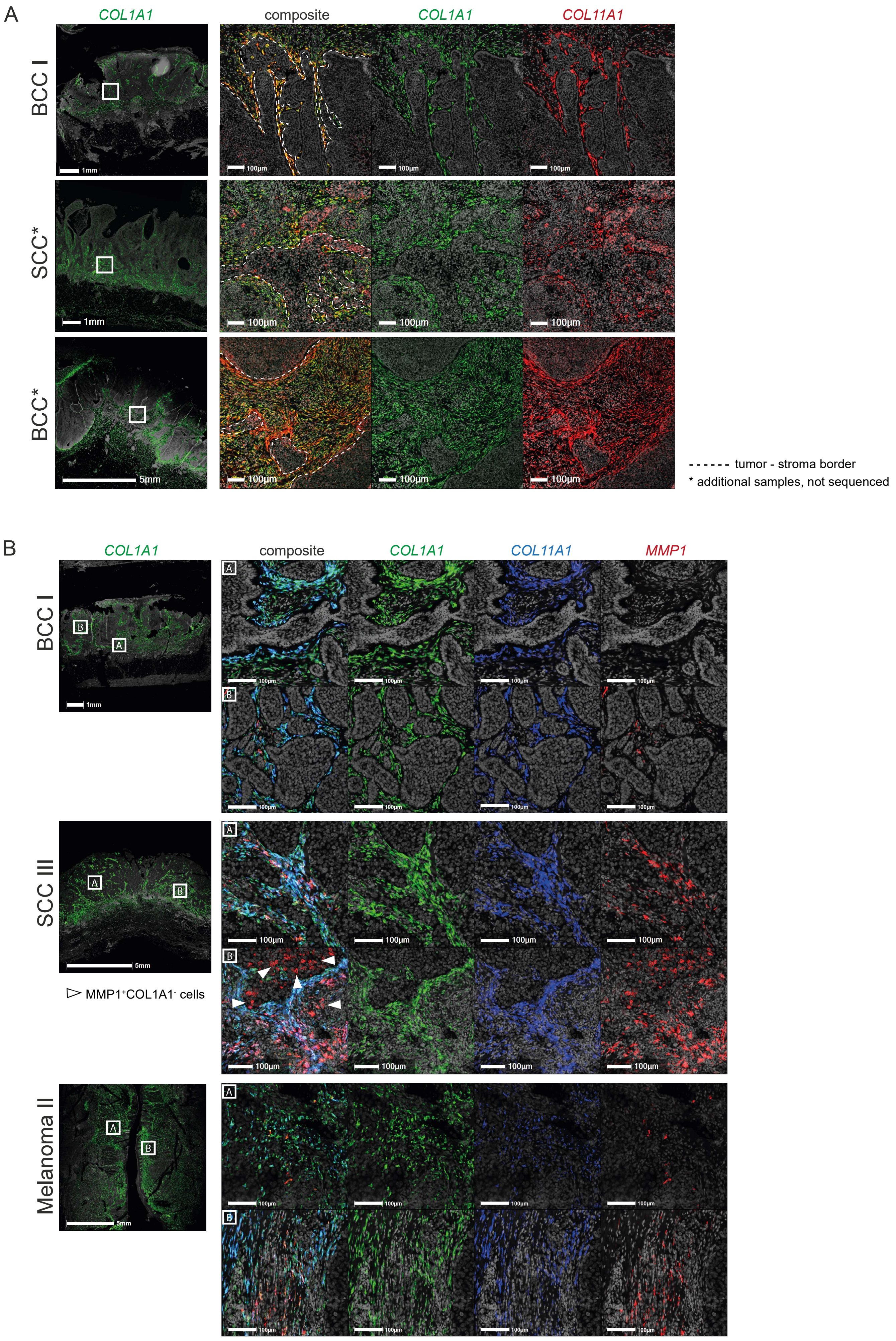

A
COL1A1
composite
COL1A1
COL11A1
BCC I
SCC*
BCC*
1mm
100µm
5mm
tumor - stroma border
* additional samples, not sequenced
B
COL1A1
composite
COL1A1
COL11A1
MMP1
BCC I
SCC III
Melanoma II
MMP1+COL1A1- cells

Figure S6. RNAScope staining for mCAFs and iCAFs. Related to Figure 5.
(A) Representative images (n=52 independent tumor samples) of RNAScope fluorescence stainings from BCC and SCC samples: *COL1A1* (green), *COL11A1* (red), DAPI (grey). Dashed line showing the tumor-stroma border. (B) Representative images of RNAScope fluorescence stainings from BCC, SCC and Melanoma: COL1A1 (green), *COL11A1* (blue), *MMP1* (red) and DAPI (grey).

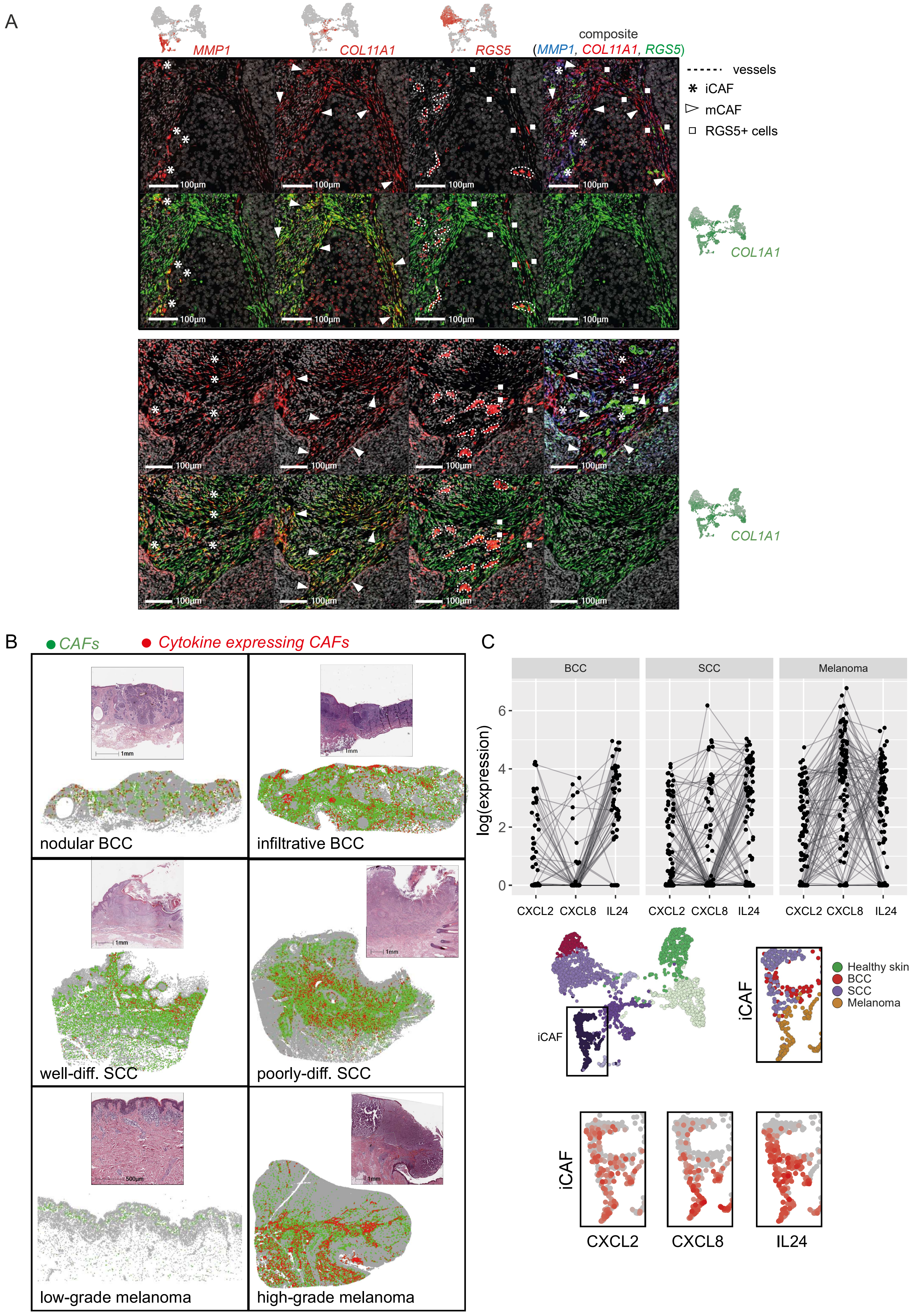

A
MMP1
COL11A1
RGS5
composite
(MMP1, COL11A1, RGS5)
vessels
iCAF
mCAF
RGS5+ cells
100µm
COL1A1
B
CAFs
Cytokine expressing CAFs
nodular BCC
infiltrative BCC
well-diff. SCC
poorly-diff. SCC
low-grade melanoma
high-grade melanoma
C
BCC
SCC
Melanoma
log(expression)
CXCL2 CXCL8 IL24
iCAF
Healthy skin
BCC
SCC
Melanoma
CXCL2
CXCL8
IL24

Figure S7. Spatial analysis of CAF subtypes *in situ.* Related to Figures 4, 5 and 6.

(A) Representative images (n=52 independent tumor samples) of RNAScope fluorescence stainings for iCAFs, mCAFs and $RGS5^{+}$cells: *MMP1, COL11A1* and *RGS5* shown as single stainings (*red*) or in combination with *COL1A1 (green), MMP1 (blue)*, *COL11A1 (red) or RGS5 (green)* in composite images.

(B) Spatial plots highlighting the spatial distribution of total CAFs (*COL1A1*) and cytokine expressing CAFs (co-expression of *COL1A1* and *CXCL2, CXCL8* or *IL24),* representative for 52 independent tumor samples.

(C) *CXCL2, CXCL8* or *IL24* expression in fibroblasts of BCC, SCC or melanoma samples that contribute to the iCAF cluster (cells from n=8 donors).

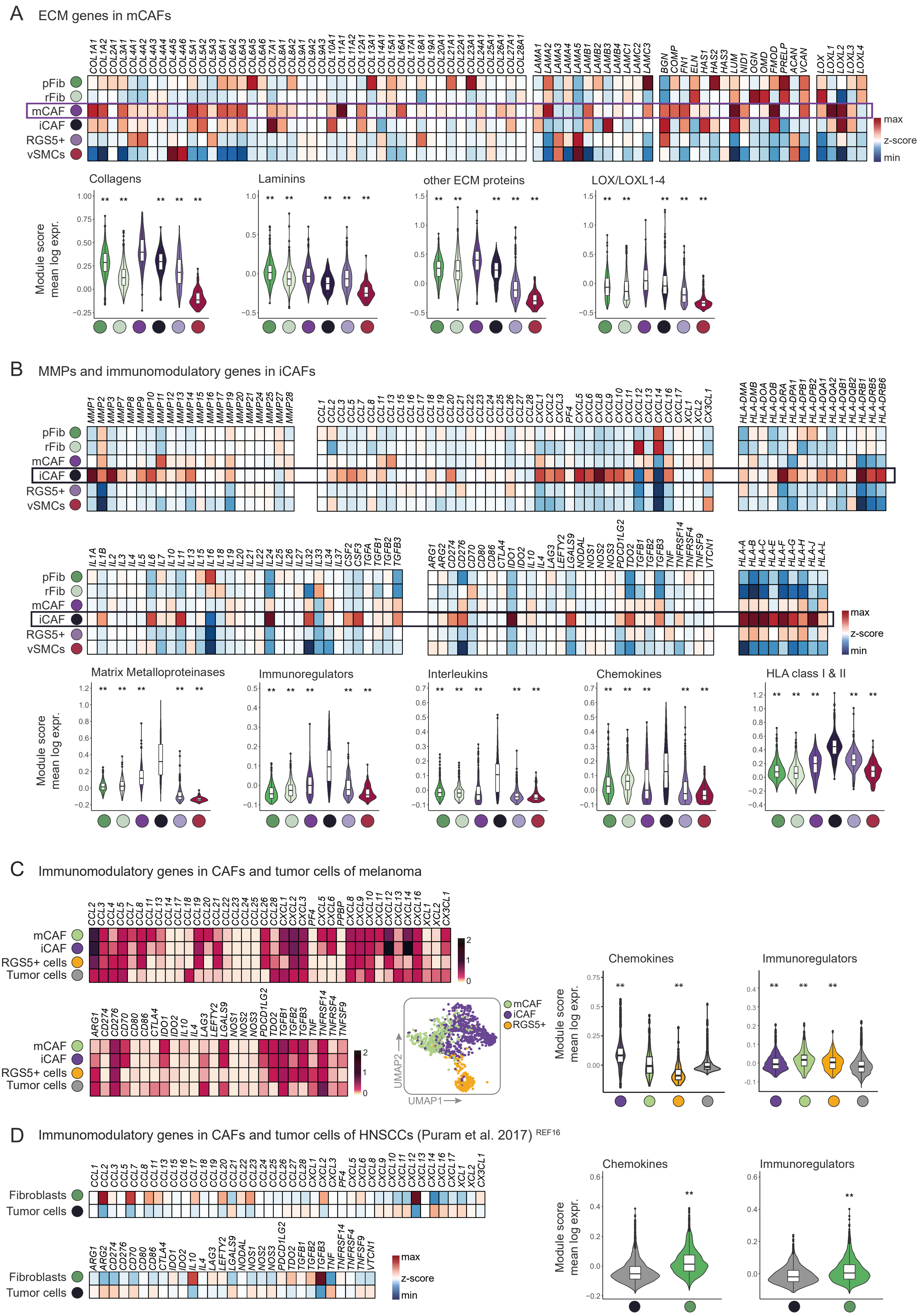
A ECM genes in mCAFs
pFib
rFib
mCAF
iCAF
RGS5+
vSMCs
max
z-score
min
Collagens
Laminins
other ECM proteins
LOX/LOXL1-4
Module score
mean log expr.
B MMPs and immunomodulatory genes in iCAFs
Matrix Metalloproteinases
Immunoregulators
Interleukins
Chemokines
HLA class I & II
C Immunomodulatory genes in CAFs and tumor cells of melanoma
mCAF
iCAF
RGS5+ cells
Tumor cells
RGS5+
UMAP1
UMAP2
Chemokines
Immunoregulators
D Immunomodulatory genes in CAFs and tumor cells of HNSCCs (Puram et al. 2017) REF16
Fibroblasts
Tumor cells
Chemokines
Immunoregulators

Figure S8. Matrix-associated, MMP and immunomodulatory gene panels characterize mCAFs and iCAFs. Related to Figures 5 and 6.

(A) Collagens, Laminins, Lysyl oxidases and other ECM proteins as well as (B) matrix metalloproteinases, chemokines, cytokines and immunomodulatory molecules represented in heatmaps and corresponding Violin plots showing module scores (n=15 donors).

(C,D) Expression of cytokines, chemokines and immunoregulatory molecules in fibroblasts or tumor cells of melanoma (C; n=5 donors) or HNSCC (D; dataset of Puram et al., 2017[16]) represented in heatmaps and corresponding Violin plots showing module scores. UMAP in (C) shows identified CAF subsets in melanoma. Statistical analysis by Wilcoxon test, reference group mCAFs (A), iCAFs (B) or tumor cells (C,D), p-value ** < 0.01, * < 0.05.

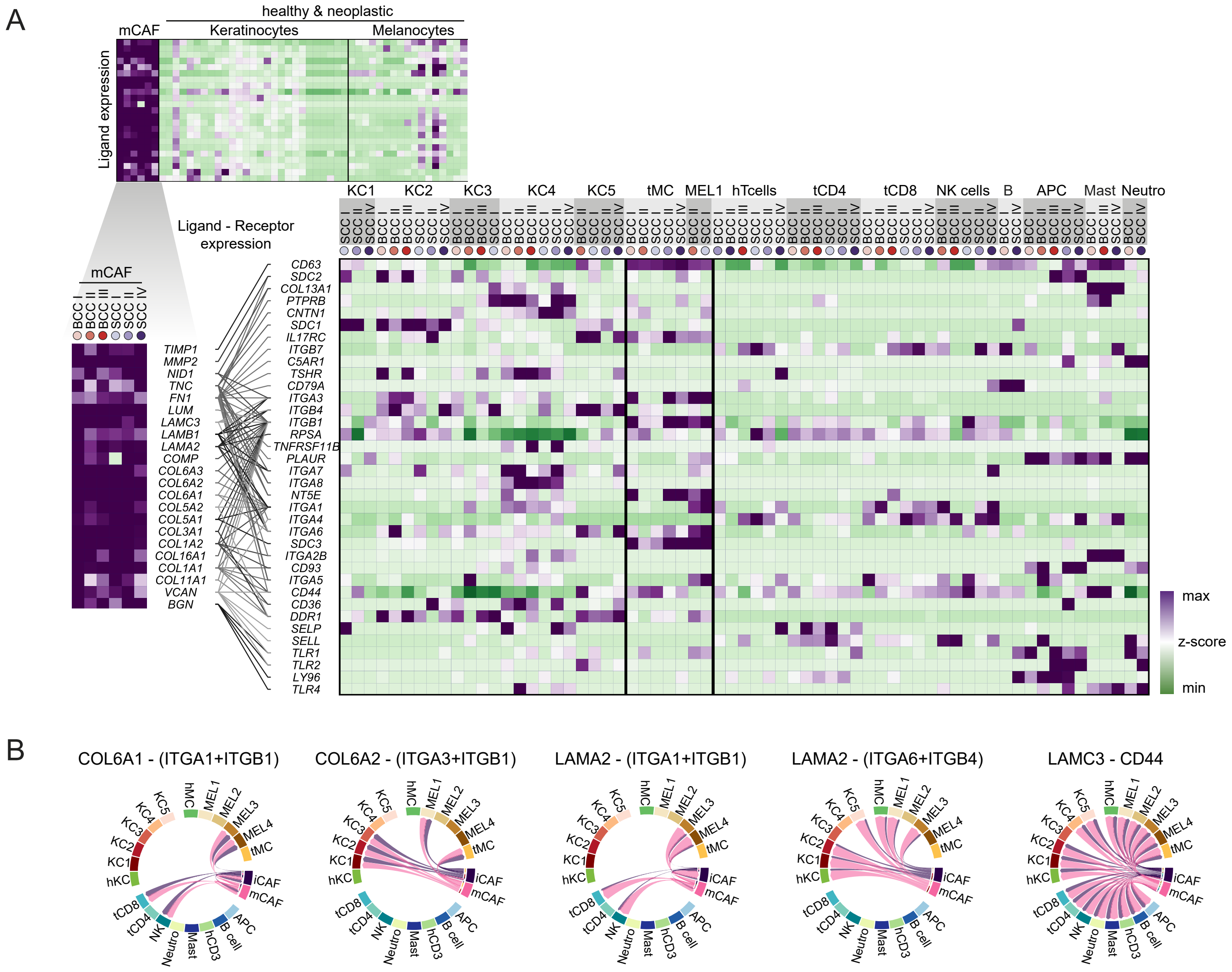
A
healthy & neoplastic
mCAF
Keratinocytes
Melanocytes
Ligand expression
Ligand - Receptor expression
mCAF
BCC I
BCC II
BCC III
SCC I
SCC II
SCC IV
TIMP1
MMP2
NID1
TNC
FN1
LUM
LAMC3
LAMB1
LAMA2
COMP
COL6A3
COL6A2
COL6A1
COL5A2
COL5A1
COL3A1
COL1A2
COL16A1
COL1A1
COL11A1
VCAN
BGN
KC1
KC2
KC3
KC4
KC5
tMC
MEL1
hTcells
tCD4
tCD8
NK cells
B
APC
Mast
Neutro
CD63
SDC2
COL13A1
PTPRB
CNTN1
SDC1
IL17RC
ITGB7
C5AR1
TSHR
CD79A
ITGA3
ITGB4
ITGB1
RPSA
TNFRSF11B
PLAUR
ITGA7
ITGA8
NT5E
ITGA1
ITGA4
ITGA6
SDC3
ITGA2B
CD93
ITGA5
CD44
CD36
DDR1
SELP
SELL
TLR1
TLR2
LY96
TLR4
max
z-score
min
B
COL6A1 - (ITGA1+ITGB1)
COL6A2 - (ITGA3+ITGB1)
LAMA2 - (ITGA1+ITGB1)
LAMA2 - (ITGA6+ITGB4)
LAMC3 - CD44
hMC
MEL1
MEL2
MEL3
MEL4
tMC
iCAF
mCAF
APC
B cell
hCD3
Mast
Neutro
NK
tCD4
tCD8
hKC
KC1
KC2
KC3
KC4
KC5

Figure S9. Receptor-Ligand analysis of mCAFs. Related to Figure 5.

(A) Expression of ECM genes in mCAFs compared to healthy and neoplastic keratinocytes and melanocytes (top heatmap) and interrogation for corresponding receptors in healthy and neoplastic keratinocytes and melanocytes as well as immune cells (n=15 donors).

(B) Circular plots of selected receptor-ligand pairs from CellChat analysis, showing mCAF/iCAF as source cells for ligand expression (n=15 donors).

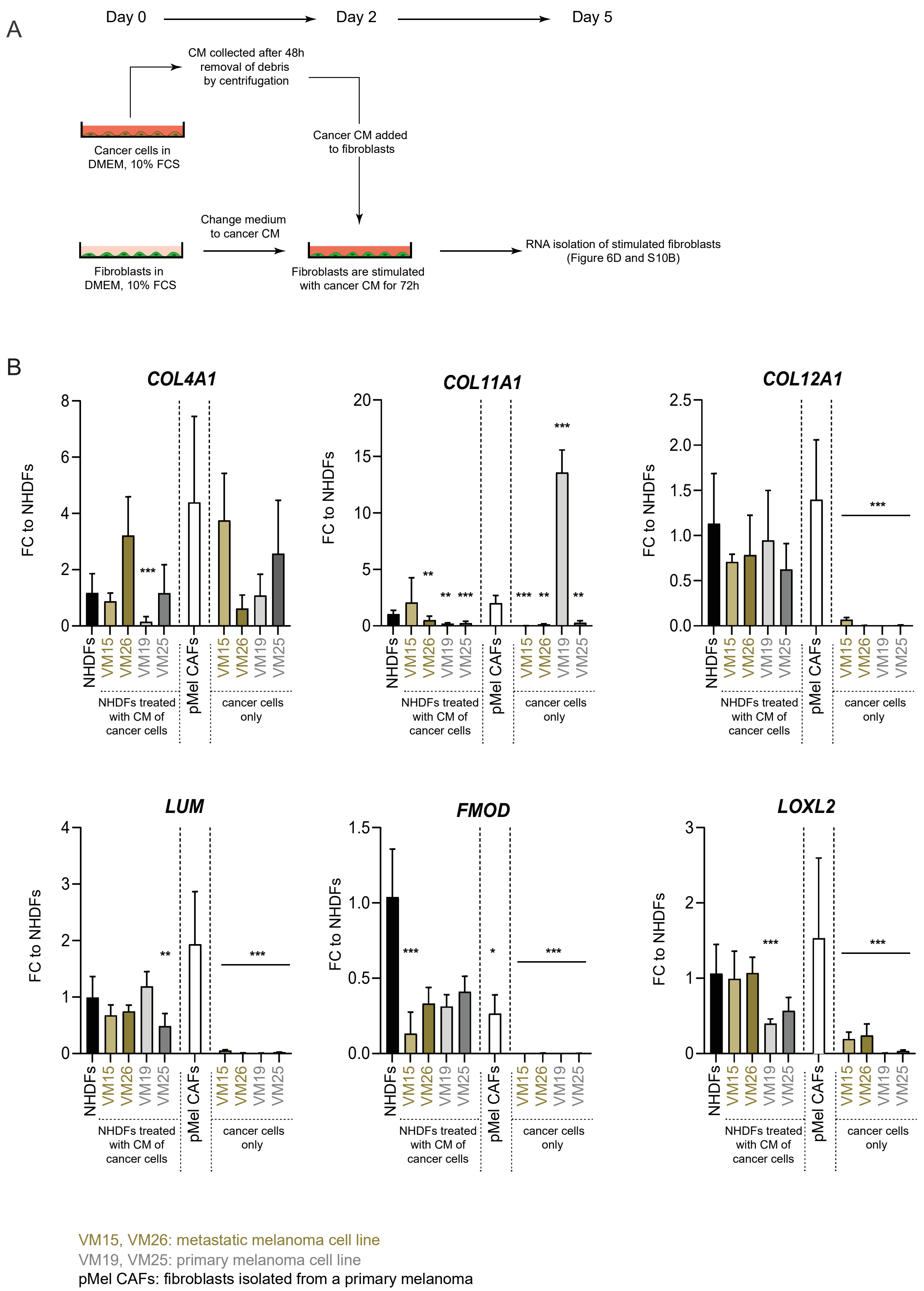

A
Day 0
Day 2
Day 5
CM collected after 48h
removal of debris
by centrifugation
Cancer cells in
DMEM, 10% FCS
Cancer CM added
to fibroblasts
Change medium
to cancer CM
Fibroblasts in
DMEM, 10% FCS
Fibroblasts are stimulated
with cancer CM for 72h
RNA isolation of stimulated fibroblasts
(Figure 6D and S10B)
B
COL4A1
COL11A1
COL12A1
LUM
FMOD
LOXL2
FC to NHDFs
NHDFs
VM15
VM26
VM19
VM25
pMel CAFs
NHDFs treated
with CM of
cancer cells
cancer cells
only
VM15, VM26: metastatic melanoma cell line
VM19, VM25: primary melanoma cell line
pMel CAFs: fibroblasts isolated from a primary melanoma

Figure S10. Expression of ECM genes is not upregulated in NHDFs upon treatment with cancer cell-derived CM. Related to Figure 6.

(A) Experimental setup for assessing transcriptional changes in NHDFs after stimulation with cancer-cell derived CM.

(B) *In vitro* ECM expression of NHDFs after exposure to conditioned medium from NHDFs, VM15, VM26, VM19 and VM25 for 72 hours in comparison to CAFs isolated from a primary melanoma (pMel CAFs) and to the cancer cell lines VM15, VM26, VM19 and VM25. Data was derived from four independent experiments and is presented as bar graphs showing mean values +/- SD. Statistical analysis by One-way-ANOVA and Tukey`s post hoc test; Significant comparisons to NHDFs are shown; Source data and exact p values are provided in the Source Data file. p<0.05, **p<0.01, ***p<0.001.

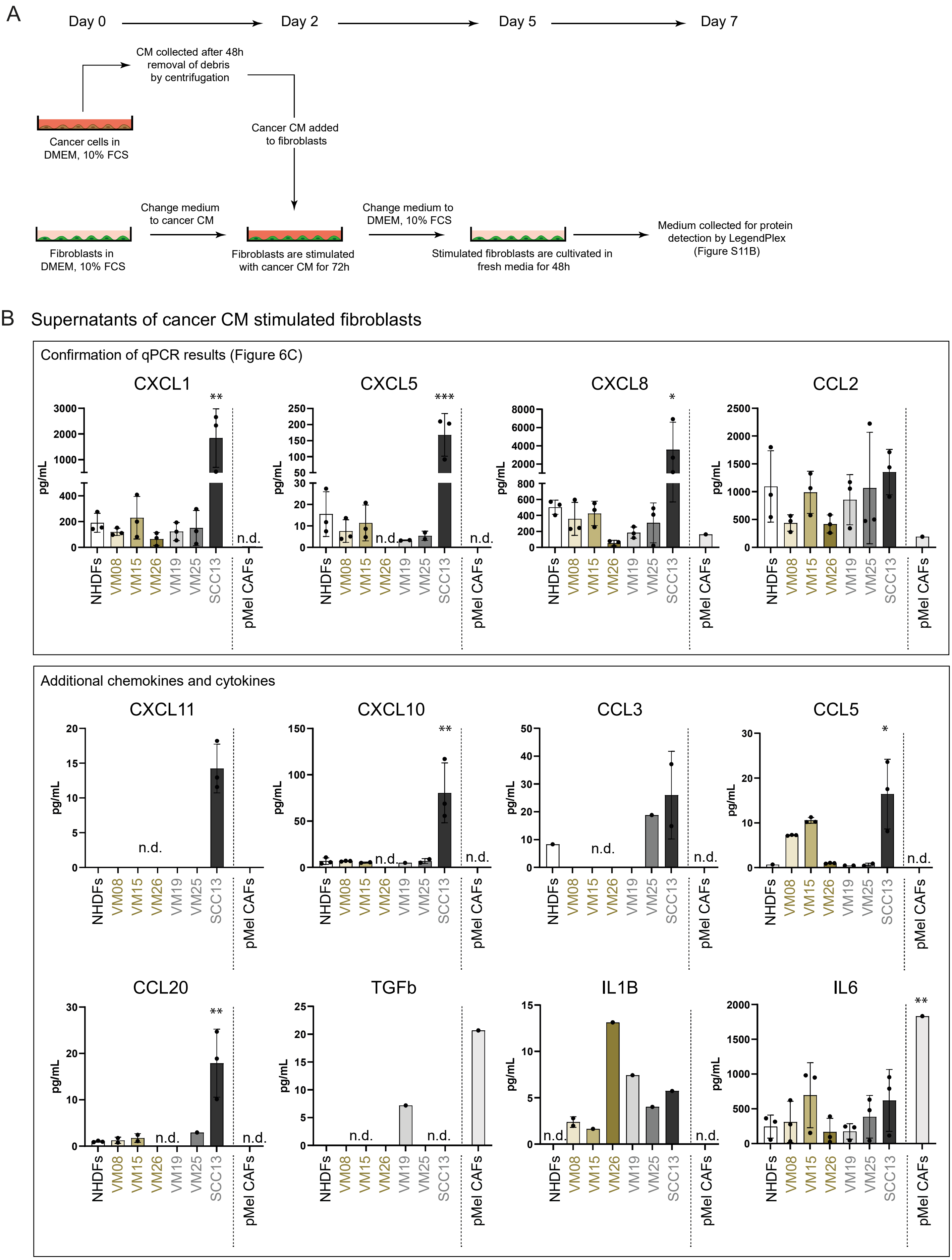

n.d. - not detected/below lowest concentration of standard

Figure S11. Protein expression in supernatants of fibroblasts upon stimulation with cancer cell-derived CM. Related to Figure 6.

(A) Experimental setup for generation of supernatants of NHDFs stimulated with cancer cell-derived CM.

(B) Protein levels (pg/mL) of several cytokines and chemokines assessed by LegendPlex. Protein levels were derived from three independent experiments (apart from pMel CAFs, n=1). Statistical analysis by One-way-ANOVA and Tukey`s post hoc test; Significant comparisons to NHDFs are shown; Source data and exact p values are provided in the Source Data file. *p<0.05, **p<0.01, ***p<0.001.

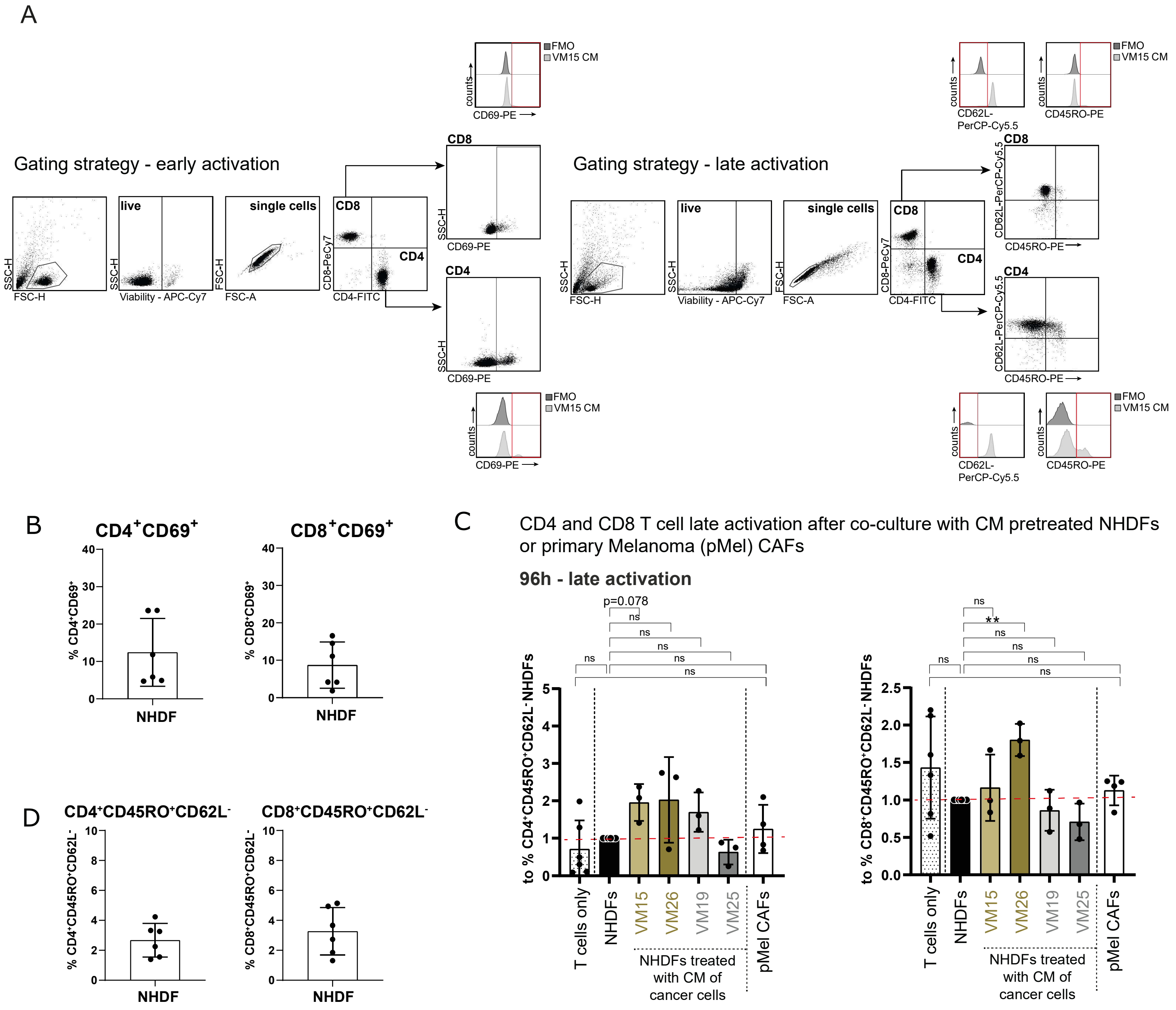

A
Gating strategy - early activation
Gating strategy - late activation
B
CD4+CD69+
CD8+CD69+
NHDF
C
CD4 and CD8 T cell late activation after co-culture with CM pretreated NHDFs or primary Melanoma (pMel) CAFs
96h - late activation
T cells only
NHDFs
VM15
VM26
VM19
VM25
pMel CAFs
NHDFs treated with CM of cancer cells
D
CD4+CD45RO+CD62L-
CD8+CD45RO+CD62L-
NHDF

Figure S12. Fibroblast-mediated late activation of CD4 and CD8 T cells. Related to Figure 7.

(A) Gating strategy for detection of early and late activation in CD4 and CD8 T cells including FMO controls for CD69, CD62L and CD45RO.

(B) Percentage of $CD4^{+}CD69^{+}$ and $CD8^{+}CD69^{+}$ T cells in co-culture with control NHDFs, n=6 biologically independent samples.

(C) Late activation (CD45RO+CD62L-) of CD4 or CD8 T cells after 96h of co-culture with CM pre-treated NHDFs or cancer cells. T cells from n=3 biologically independent samples in co-culture with VM15, VM26, VM19, VM25, n=6 biologically independent samples (T cells only, NHDFs) and n=4 biologically independent samples (pMel CAFs). Data are presented as mean values +/- SD. Statistical analysis in comparison to NHDFs or to T cells only by unpaired Student's t test; Source data and exact p values are provided in the Source Data file. *p<0.05, **p<0.01, ***p<0.001.

(D) Percentage of $CD4^{+}CD45RO^{+}CD62L^{-}$ and $CD8^{+}CD45RO^{+}CD62L^{-}$ in co-culture with control NHDFs, n=6 biologically independent samples.

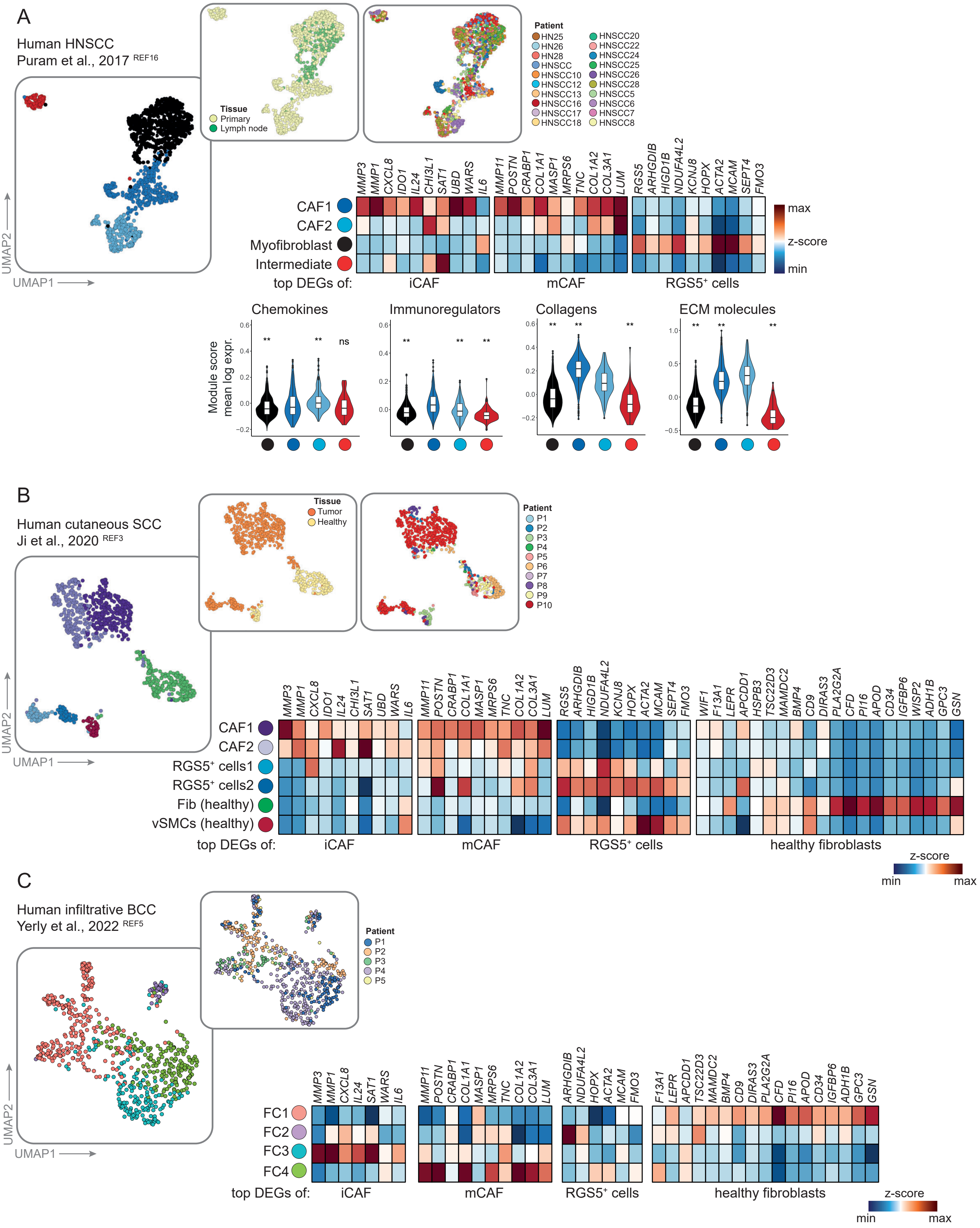

A
Human HNSCC
Puram et al., 2017 REF16
UMAP2
UMAP1
Tissue
Primary
Lymph node
Patient
HN25
HN26
HN28
HNSCC
HNSCC10
HNSCC12
HNSCC13
HNSCC16
HNSCC17
HNSCC18
HNSCC20
HNSCC22
HNSCC24
HNSCC25
HNSCC26
HNSCC28
HNSCC5
HNSCC6
HNSCC7
HNSCC8
MMP3 MMP1 CXCL8 IDO1 IL24 CHI3L1 SAT1 UBD WARS IL6
MMP11 POSTN CRABP1 COL1A1 MASP1 MRPS6 TNC COL1A2 COL3A1 LUM
RGS5 ARHGDIB HIGD1B NDUFA4L2 KCNJ8 HOPX ACTA2 MCAM SEPT4 FMO3
CAF1
CAF2
Myofibroblast
Intermediate
top DEGs of:
iCAF
mCAF
RGS5⁺ cells
max
z-score
min
Chemokines
Immunoregulators
Collagens
ECM molecules
Module score
mean log expr.
ns
B
Human cutaneous SCC
Ji et al., 2020 REF3
Tissue
Tumor
Healthy
Patient
P1
P2
P3
P4
P5
P6
P7
P8
P9
P10
MMP3 MMP1 CXCL8 IDO1 IL24 CHI3L1 SAT1 UBD WARS IL6
MMP11 POSTN CRABP1 COL1A1 MASP1 MRPS6 TNC COL1A2 COL3A1 LUM
RGS5 ARHGDIB HIGD1B NDUFA4L2 KCNJ8 HOPX ACTA2 MCAM SEPT4 FMO3
WIF1 F13A1 LEPR APCDD1 HSPB3 TSC22D3 MAMDC2 BMP4 CD9 DIRAS3 PLA2G2A CFD PI16 APOD CD34 IGFBP6 WISP2 ADH1B GPC3 GSN
CAF1
CAF2
RGS5⁺ cells1
RGS5⁺ cells2
Fib (healthy)
vSMCs (healthy)
top DEGs of:
iCAF
mCAF
RGS5⁺ cells
healthy fibroblasts
z-score
min
max
C
Human infiltrative BCC
Yerly et al., 2022 REF5
Patient
P1
P2
P3
P4
P5
MMP3 MMP1 CXCL8 IL24 SAT1 WARS IL6
MMP11 POSTN CRABP1 COL1A1 MASP1 MRPS6 TNC COL1A2 COL3A1 LUM
ARHGDIB NDUFA4L2 HOPX ACTA2 MCAM FMO3
F13A1 LEPR APCDD1 TSC22D3 MAMDC2 BMP4 CD9 DIRAS3 PLA2G2A CFD PI16 APOD CD34 IGFBP6 ADH1B GPC3 GSN
FC1
FC2
FC3
FC4
top DEGs of:
iCAF
mCAF
RGS5⁺ cells
healthy fibroblasts
z-score
min
max

Figure S13. Reanalysis of published datasets reveals CAF subsets with gene signatures similar to mCAFs, iCAFs and RGS5$^+$ cells in human oral and cutaneous SCC as well as invasive BCC.

(A) UMAPs, heatmaps of top DEG from mCAFs, iCAFs and RGS5$^+$ cells and module scores of chemokines, immunomodulators, collagens and other ECM molecules of CAFs in human oral SCC (Puram et al.,2017) [16].

(B,C) UMAPs and heatmaps of top DEG from mCAFs, iCAFs, RGS5$^+$ cells and healthy fibroblasts from human cutaneous SCC (Ji et al., 2022) [3] (B) or BCC (Yerly et al., 2022) [5] (C).

REFERENCES

1. Tirosh, I., Izar, B., Prakadan, S.M., Wadsworth, M.H., Treacy, D., Trombetta, J.J., Rotem, A., Rodman, C., Lian, C., Murphy, G., et al. (2016). Dissecting the multicellular ecosystem of metastatic melanoma by single-cell RNA-seq. Science *352*, 189–196. https://doi.org/10.1126/science.aad0501.

2. Jerby-Arnon, L., Shah, P., Cuoco, M.S., Rodman, C., Su, M.-J., Melms, J.C., Leeson, R., Kanodia, A., Mei, S., Lin, J.-R., et al. (2018). A Cancer Cell Program Promotes T Cell Exclusion and Resistance to Checkpoint Blockade. Cell *175*, 984-997.e24. https://doi.org/10.1016/j.cell.2018.09.006.

3. Ji, A.L., Rubin, A.J., Thrane, K., Jiang, S., Reynolds, D.L., Meyers, R.M., Guo, M.G., George, B.M., Mollbrink, A., Bergenstråhle, J., et al. (2020). Multimodal Analysis of Composition and Spatial Architecture in Human Squamous Cell Carcinoma. Cell *182*, 497-514.e22. https://doi.org/10.1016/j.cell.2020.05.039.

4. Guerrero-Juarez, C.F., Lee, G.H., Liu, Y., Wang, S., Karikomi, M., Sha, Y., Chow, R.Y., Nguyen, T.T.L., Iglesias, V.S., Aasi, S., et al. (2022). Single-cell analysis of human basal cell carcinoma reveals novel regulators of tumor growth and the tumor microenvironment. Sci. Adv. *8*, eabm7981. https://doi.org/10.1126/sciadv.abm7981.

5. Yerly, L., Pich-Bavastro, C., Di Domizio, J., Wyss, T., Tissot-Renaud, S., Cangkrama, M., Gilliet, M., Werner, S., and Kuonen, F. (2022). Integrated multi-omics reveals cellular and molecular interactions governing the invasive niche of basal cell carcinoma. Nat Commun *13*, 4897. https://doi.org/10.1038/s41467-022-32670-w.

6. Ganier, C., Mazin, P., Herrera-Oropeza, G., Du-Harpur, X., Blakeley, M., Gabriel, J., Predeus, A.V., Cakir, B., Prete, M., Harun, N., et al. (2024). Multiscale spatial mapping of cell populations across anatomical sites in healthy human skin and basal cell carcinoma. Proc. Natl. Acad. Sci. U.S.A. *121*, e2313326120. https://doi.org/10.1073/pnas.2313326120.

7. Schütz, S., Solé-Boldo, L., Lucena-Porcel, C., Hoffmann, J., Brobeil, A., Lonsdorf, A.S., Rodríguez-Paredes, M., and Lyko, F. (2023). Functionally distinct cancer-associated fibroblast subpopulations establish a tumor promoting environment in squamous cell carcinoma. Nat Commun *14*, 5413. https://doi.org/10.1038/s41467-023-41141-9.

8. Korosec, A., Frech, S., Gesslbauer, B., Vierhapper, M., Radtke, C., Petzelbauer, P., and Lichtenberger, B.M. (2019). Lineage Identity and Location within the Dermis Determine the Function of Papillary and Reticular Fibroblasts in Human Skin. Journal of Investigative Dermatology *139*, 342–351. https://doi.org/10.1016/j.jid.2018.07.033.

9. Philippeos, C., Telerman, S.B., Oulès, B., Pisco, A.O., Shaw, T.J., Elgueta, R., Lombardi, G., Driskell, R.R., Soldin, M., Lynch, M.D., et al. (2018). Spatial and Single-Cell Transcriptional Profiling Identifies Functionally Distinct Human Dermal Fibroblast Subpopulations. Journal of Investigative Dermatology *138*, 811–825. https://doi.org/10.1016/j.jid.2018.01.016.

10. Tabib, T., Morse, C., Wang, T., Chen, W., and Lafyatis, R. (2018). SFRP2/DPP4 and FMO1/LSP1 Define Major Fibroblast Populations in Human Skin. Journal of Investigative Dermatology *138*, 802–810. https://doi.org/10.1016/j.jid.2017.09.045.

11. Janson, D.G., Saintigny, G., van Adrichem, A., Mahé, C., and El Ghalbzouri, A. (2012). Different Gene Expression Patterns in Human Papillary and Reticular Fibroblasts. Journal of Investigative Dermatology *132*, 2565–2572. https://doi.org/10.1038/jid.2012.192.

12. Nurmik, M., Ullmann, P., Rodriguez, F., Haan, S., and Letellier, E. (2020). In search of definitions: Cancer-associated fibroblasts and their markers. Int. J. Cancer *146*, 895–905. https://doi.org/10.1002/ijc.32193.

13. Han, M., Liu, Z., Liu, L., Huang, X., Wang, H., Pu, W., Wang, E., Liu, X., Li, Y., He, L., et al. (2023). Dual genetic tracing reveals a unique fibroblast subpopulation modulating cardiac fibrosis. Nat Genet *55*, 665–678. https://doi.org/10.1038/s41588-023-01337-7.

14. Gascard, P., and Tlsty, T.D. (2016). Carcinoma-associated fibroblasts: orchestrating the composition of malignancy. Genes Dev. *30*, 1002–1019. https://doi.org/10.1101/gad.279737.116.

15. Bartoschek, M., Oskolkov, N., Bocci, M., Lövrot, J., Larsson, C., Sommarin, M., Madsen, C.D., Lindgren, D., Pekar, G., Karlsson, G., et al. (2018). Spatially and functionally distinct subclasses of breast cancer-associated fibroblasts revealed by single cell RNA sequencing. Nat Commun *9*, 5150. https://doi.org/10.1038/s41467-018-07582-3.

16. Puram, S.V., Tirosh, I., Parikh, A.S., Patel, A.P., Yizhak, K., Gillespie, S., Rodman, C., Luo, C.L., Mroz, E.A., Emerick, K.S., et al. (2017). Single-Cell Transcriptomic Analysis of Primary and Metastatic Tumor Ecosystems in Head and Neck Cancer. Cell *171*, 1611-1624.e24. https://doi.org/10.1016/j.cell.2017.10.044.
