# Supplementary material for: Cancer-associated fibroblast subtypes modulate the tumor-immune microenvironment and are associated with skin cancer malignancy
Source: Nat Commun. 2024 Nov 8;15:9678. doi: 10.1038/s41467-024-53908-9 (PMC11549091; doi:10.1038/s41467-024-53908-9)
Supplement: Supplementary file 3 — Source Data [file 41467_2024_53908_MOESM3_ESM.pdf]

nature portfolio

Peer Review File

Cancer associated fibroblast subtypes modulate the tumor-immune microenvironment and are associated with skin cancer malignancy

Corresponding Author: Professor Beate Lichtenberger

Parts of this Peer Review File have been redacted as indicated to remove third-party material.

This file contains all reviewer reports in order by version, followed by all author rebuttals in order by version.

A version of this paper was originally rejected for publication by Nature Communications, however that decision was reconsidered after appeal by the authors.

Version 0:

Reviewer comments:

Reviewer #1

(Remarks to the Author)
This manuscript by Forsthuber and colleagues examine CAF types in the three types of skin cancer. The investigators show that mCAFs arise in early stage tumors and produce extra-cellular matrix to limit immunosurveillance. The authors also show that iCAFs are present primarily in aggressive lesions and produce chemokines regulating immune cell recruitment and activation.

This manuscript addresses a knowledge deficit regarding the molecular phenotype of fibroblasts in present in skin cancers in the TME. The data provide insights regarding potential regulatory functions for fibroblasts in regulating skin cancers. Therefore, the data are interesting and of value to the broader research community. However, there are a number of issues listed below that should be addressed.

The following issues should be addressed:

1. Intro, first line, Understudied is probably a more accurate term than Underestimated.

2. page 3, line 61-3, This ref is from 2006, much improvement in melanoma therapeutic outcomes has happened in the interval primarily involving use of immunomodulatory therapies. Poorer outcomes for metastatic diseases were typical prior to 2006 but significantly better now. Perhaps this reality could be worked into the discussion.

3. The terminology used in Sup Table 1 needs clarification.

a. The BCCs analyzed should be classified regarding their histologic type, superficial multicentric, nodular, infiltrative, morpheaform, etc.

b) What is "Bowen carcinoma". Typically Bowen's disease in the skin indicates a form of squamous cell carcinoma in situ. Do you mean squamous cell carcinoma arising from a pre-existing Bowen's disease lesion? If it is CIS probably best to call it Bowen's Disease.

c) Instead of highly differentiated, better to say 'well differentiated'.

4. Can the authors address why they felt not matching the healthy skin samples with the tumor samples for site and sex is appropriate? There is a strong female bias in the control samples.

5. The overall cell number sequenced from the tumor and skin samples seems low. Was this the experimental strategy?

6. Fig 6D, the terminology used is confusing. What is a non-invasive BCC, differentiated SCC, SSM? By definition, these lesions invade the dermis.

7. line 318, none of the tumors analyzed are benign as listed in 6b, they are various types of carcinomas or melanoma with varying potentials to metastasize and/or spread locally. So it is confusing to describe a subset as benign. Better to say low-grade for lesions that have a low probability of metastasis and high-grade for lesions that with a higher probability of metastasis.

8. Were there any differences in the transcription factor expression profiles between the iCAFs and mCAFs?

Reviewer #2

(Remarks to the Author)
In this paper, Forsthuber et al., presented a single-cell RNA-seq study of sorted cell populations including keratinocytes, fibroblasts and immune cells across three skin cancer types, melanoma, BCC and SCC, using smart-seq2, with a focus on the intra-tumoral heterogeneity of CAFs. They identified three main CAF types, mCAF, iCAF and RGS5+ CAFs, and found mCAFs were more abundant in early-stage tumors, while iCAF were more enriched in aggressive tumors. These findings were further validated by RNAscope and IHC in 39 tumors. Overall, the manuscript is well written. It provides valuable resources to the skin cancer research community.

However, the whole paper felt a bit descriptive in some places (although this is often due to the nature of scRNA-seq studies), and it needs more statistical power and analysis to make the findings robust. My specific comments are as follows,

1. Quantification of RNAscope and IHC. Although the study performed RNAscope and IHC in situ validation of some top markers in 39 tumors, the results were just descriptive with representative images shown. Can the authors quantify all the staining data, and compare them between cancer types and stages? Then the proper statistics can be derived. This relates main figures 4-6.
2. Clinical inference and importance. Although authors included different cancer types and various stages, the numbers of samples were too low to derive robust clinical inference. Using various published and publicly available bulk RNA-seq data sets of melanoma, BCC and SCC, can authors perform bulk tissue deconvolution based on their identified signatures, and then test
- if the difference of various CAFs among different cancer types can be validated using bulk RNA-seq as well
- if the abundance and intensity of various CAFs are associated with important clinical outcome, e.g., survival/metastasis in melanoma, in situ vs. invasive SCC, metastasising vs non-metastasising tumours?
3. Cell-cell communication. In figure 5, the author performed the receptor ligand analysis using the scRNA-seq data. Can authors use some commonly used cell-cell communication tools, such as cellphoneDB, cellChat, Celltalker, to validate all the cell-cell communication results?
4. Trajectory. Can authors perform the trajectory analysis on fibroblasts to further study the developmental trajectory of fibroblasts. It may give some clue of the origin of CAFs.

Some minor points,

Line 288-290. “iCAFs….. are an exclusive source of many chemokines (Figure 5D).”. How was this analysis done? What is the evidence to support “being exclusive”?

Figure 6B. Could statistics be done on figure 6b to identify significance?

Line 311-312. “….resulted in a proliferative and activated iCAF-like phenotype in vitro (Figure 6C).” where is the data supporting “proliferative”? I cannot see it.

Reviewer #3

(Remarks to the Author)
The manuscript integrates scRNAseq and spatial RNA FISH staining from human BCC, SCC and melanoma to decipher the commonalities and specificities of CAF heterogeneity. The authors identifiy 3 cancer associated CAFs phenotypes, RGS5+, iCAF and mCAF. mCAF are defined by high expression of matrix-associated genes and mostly found in BCC and SCC, while iCAF are defined by the expression of immunomodulatory genes, and preferentially found in invasive melanoma and poorly-differentiated SCC. A panel of immunomodulatory cytokines is induced by treatment of human dermal fibroblasts with the conditioned medium of melanoma and SCC cell lines. The manuscript provides a deep molecular single-cell and spatial characterization of CAFs heterogeneity in skin cancer. However, in our opinion, lacks clear identification of tumor cells and requires stronger validation using independent scRNAseq cohorts and additional stainings.

Major comments:
The authors claim that the unsupervised clustering separated healthy and malignant keratinocytes (line 90 and line 96). However, the authors do not show how they discriminate normal neoplastic keratinocytes from normal keratinocytes, which are expected to be mixed in BCC and SCC samples. Similarly, the authors do not show how they discriminate neoplastic from normal melanocytes coming from melanoma samples. Indeed, the CNV analysis shown in Fig 2C and Supp 2A shows

high intra-sample heterogeneity, with high proportions of CNV negative cells. Samples like BCCI, BCCII, BCCIII, SCCI and SCCII have indeed very low number of CNV positive cells. How do the authors make sure they have indeed tumor cells in significant proportions in theses samples? This important concern largely precludes from further interpretation of the data. Do the authors identify a cycling cell cluster?
The authors claim that single-cell studies on human skin cancer included only a few or no fibroblasts (line 36). This is clearly untrue, as we found existing scRNAseq with significant fibroblast coverage for SCC (DOI: 10.1016/j.cell.2020.05.039) and BCC (DOI: 10.1126/sciadv.abm79 ; doi.org/10.1038/s41467-022-32670-w), with the last two aiming at deciphering fibroblast heterogeneity. The CAF cluster definition identified by the authors (RGS5+, mCAF and iCAF) should be analyzed in the light of and validated in the previously published independent scRNAseq cohorts.
The authors should perform co-staining of mCAF and iCAF markers on BCC, SCC and melanoma, to show that they highlight distinct spatial clusters. They should show PTGDS and MMP1 expression on scRNAseq Fibroblast clusters.
Line 268, the authors mention the infiltrative part of a Bowen, which are, by definition, non-invasive.
The authors claim that mCAF are associated with low cancer aggressiveness, while iCAF are associated with more aggressive tumors, based on scRNAseq and stainings. This assumption requires quantifications of mCAF and iCAF stainings, including additional samples with representative stages/morphological pattern of progression for each subtype. Related to this, iCAF in situ quantification method in Fig6B is unclear.
The authors claim that iCAF are the exclusive source of many chemokines (line 289). However their analysis does not include immune cells or endothelial cells, which are very probably involved in immunomodulation of the tumor stroma too (Fig. 5D).

In Fig. 6C, the authors test the effect of cancer cell lines conditioned medium on the expression of cytokines in NHDF. How are the cytokine measured, by qRT-PCR? By ELISA? How do the authors make sure that the observed inductions are not contamination by CM, as suggested by the similar expression patterns in stimulated CM and cancer cells (left and right parts of the graphs)?
The authors claim that only metastatic melanoma and aggressive SCC lines induced iCAF cytokine expression. However, they previously found iCAF surrounding primary melanoma. They should test additional cell lines with various grades of aggressiveness. The authors should include similar experiments regarding mCAF markers.

Minor points:
Line 68: The mCAFs cluster is not new (10.3389/fmolb.2022.864302)
Figure S1: Number of cells/sample should be added
Figure S2: In the text, Figure S2a appears after S2b and S2c
Fig 3a: Unclassifiable CAF could be doublets or low quality cells. Please check.
Line 239: S4C instead of S5C
In Fig 5 a/b, S5a/b/c/d, adding a marker of tumor cells would help the interpretation

Reviewer #4

(Remarks to the Author)
This study from Forsthuber et al. attempts to uncover the role of fibroblasts in skin cancer progression using scRNA-seq, RNAscope/FISH and IHC analysis of human tissue samples. In general, the manuscript is clearly written and accurately cites relevant literature to highlight key areas of unmet need in this area of research. The figures are also produced to a very high standard. However, these data are too preliminary/descriptive to substantiate the conclusions drawn and fail to provide novel insight into fibroblast heterogeneity beyond what has been described in previous studies.

General comments on the manuscript's limitations: the experiments are not appropriately designed to accurately address the questions under investigation. For example, the scRNA-seq cohort is very small and heterogeneous; the RNAscope/IHC analysis does not provide sufficient markers (within individual multiplexed panels) to enable accurate differentiation between the cell subpopulations identified by scRNA-seq; very few (or arguably no) findings presented in the manuscript are supported by quantitative, statistically significant differences observed across biological replicates.

Comments related to specific sections of the manuscript are provided below:

Abstract:
Many points made in the abstract are not adequately supported by the data presented
• "We show that two out of three CAF subtypes contribute to tumor immune surveillance with distinct mechanisms"
o From the data presented it is not shown whether CAF subtypes contribute to tumour immune surveillance, and it is certainly not shown whether this varies by subtype. Cytokine and ligand/receptor expression is shown at the mRNA level. How this impacts immune surveillance is suggested as a result of these data and previous studies. However, neither of these are sufficient to justify this claim and demonstrate distinct mechanisms of immune surveillance in skin cancer.
• "Matrix CAFs (mCAFs), a previously unknown subtype present in early-stage tumors, ensheath tumor nests and synthesize extracellular-matrix to prevent T cell invasion."
o This fibroblast subtype has been described in many studies of different cancer types. There is no statistically significant evidence provided to show that these cells are more abundant in early-stage tumours and this cannot be assessed in such a small and heterogeneous cohort. There is also no evidence shown that the ECM production regulated by these cells prevents T-cell infiltration, this is based on findings from other studies and not demonstrated here for skin cancer.
• "Immuno CAFs (iCAFs), which express proinflammatory and immunomodulatory factors, are only detected in high abundance in aggressive tumors."

o The term iCAF is typically used to describe inflammatory CAFs (not immuno CAFs). As above there is no statistically significant evidence provided to show these are only found in aggressive tumours.
• Mechanistically, we show that cancer cells transform adjacent healthy fibroblasts into cytokine-expressing iCAFs, which subsequently recruit immune cells and modulate the immune response.
o The molecular mechanism of fibroblast activation into cytokine-expressing iCAFs is not shown. Experimentally, upregulation of cytokines in response to cancer cell conditioned media is shown, but this analysis is limited to the mRNA level. The ability of these iCAFs to recruit and modulate the immune response is not shown at all in this manuscript.
Results:

Figures 1-2. These provide context to the scRNA-seq generated but do not provide insight into the main findings presented in the manuscript (relevant to fibroblast heterogeneity) and are therefore largely supplementary. However, these data do serve to illustrate how heterogeneous the cohort analysed by scRNA-seq is.

Figure 3:
Fibroblast clustering shows a high degree of sample specific grouping in the UMAP projection. How were batch effects between samples handled and corrected for? If not (as seems to be the description in the methods) does applying such a correction impact the clustering?
There are discrepancies between the expression of previously described papillary/reticular fibroblast markers and the clusters identified in this dataset. However, this is not adequately explained or addressed. It seems that very few reticular markers are strongly expressed by the "rFIB" cluster (Figure S3C). Notably, the markers for this population do clearly overlap with PI16+ "universal fibroblast" described by Buechler et al Nature 2022 and others, would this terminology be more accurate?
Line 163, describes healthy fibs found on "transition" (denoting field cancerisation) to tumour. This comment requires further justification. What is meant by on transition? RNA velocity or similar algorithms could/should be used to verify this claim.
Describing ACTA2 as a "key signature molecule" for myCAFs in transcriptomic data is not appropriate. De novo aSMA+ stress fibre formation in addition to elevated ECM deposition is the "key signature" for activated/myo-fibroblasts. The expression of ACTA2 at the transcriptome level has been mis appropriated by a number of studies to identify activated/myo-fibroblasts and (as described by the authors) in this context is a stronger marker of pericytes and smooth muscle cells (as demonstrated by the co-expression of MCAM and RGS5). However, the absence of elevated expression of ECM genes (e.g. collagen family members) precludes the conclusion that these are myoCAFs or comparable to those cells found in fibrosis.
Description of ACTA2+ and FAP+ combination for identifying all CAFs is not novel or particularly informative given (as the authors describe) this strategy cannot differentiate between healthy mural cells and CAFs. Furthermore, this approach is then not validated or demonstrated further in the RNAscope/IHC analysis.

Figure 4:
As described above it remains unclear and variable between studies how myCAFs and pericytes are described based on transcriptome level analysis of CAFs. In figure 4 the authors seek to address this issue, which is a valuable endeavour. However, this analysis is quite cursory, consisting of discrepancies with the scRNA-seq data that remain unexplained, and therefore fails to adequately justify the conclusion that the RGS5+ cluster identified by scRNA-seq are in fact a mix of pericytes and CAFs. For example, the authors state "However, [RGS5] staining intensity was stronger in perivascular cells compared to stromal RGS5+ in the tumor tissue, which was not reflected in the scRNA-seq data". It is evident from Figure 4A that RGS5 is expressed in a subset of mCAF and iCAFs in the scRNA-seq data. So it is equally possible that the reduced expression of RGS5 in tumour stroma compared to perivascular regions could indicate that the cells found in tumour stroma reflect the mCAF/iCAF populations (not the "RGS5+" cluster). To address this question more robustly a broader panel of markers is required to specifically detect each subpopulation identified in the scRNA-seq and examine their spatial distribution, ideally across the whole tissue (rather than selected ROIs).
Additionally, there is no explanation provided for the lack of PDGFRA expression detected in any region analysed. PDGFRA expression should have been detected in adjacent skin as it is highly expressed in healthy fibroblast populations. Were controls performed to confirm accurate detection of these markers by RNAscope?

Figure 5:
The analysis of potential ligand-receptor interactions is extremely speculative and should not be considered further than hypothesis generation. To justify the conclusions drawn regarding roles in immune modulation and tumour-stroma signalling mechanisms, requires both tissue analysis to demonstrate spatial autocorrelation of the ligand-receptor pairs at the protein level and functional analysis of intercellular interactions between the relevant cell populations.

Figure 6:
The results showing tumour cell conditioned media inducing iCAF marker expression has been described previously in other cancer types (e.g. Ohlund et al Cancer Discovery 2017) so this finding is not particularly novel. The conclusion that aggressive tumours are solely capable of stimulating iCAF marker expression is interesting. However, this requires further evidence to demonstrate a significant correlation between iCAFs and tumour grade in tissues; and consistent demonstration (across more than one cell line) that conditioned media from well differentiated/non-aggressive skin cancer and normal epithelium fails to induce iCAF marker expression in this in vitro system.

Version 1:

Reviewer comments:

Reviewer #2

(Remarks to the Author)
The authors have done tremendous amount of revision work that has further strengthen the paper. They have addressed all my previous comments.

Reviewer #3

(Remarks to the Author)
We thank the authors for the efforts provided. Although the authors answered most of my points, they still did not show that every individual tumor sample actually contains tumor cells.
Fig. 2A clearly shows that unsupervised clustering does not differentiate between normal and neoplastic keratinocytes, as illustrated by the mixed composition of KC2, KC3, KC4 and KC5. Moreover, CNV analysis as performed in Fig. 2C, does not identify neoplastic keratinocytes in various BCC and SCC samples. Finally, the authors do not show how far the expression of gene markers like PTCH1/2 are shared across individual tumor samples.
Our suggestion here would be to perform CNV analysis in individual tumor samples on keratinocytes only (and melanocytes only), using T cells (or stromal cells) as reference. Of note, proliferating cells which display a strongly modified transcriptome, should be excluded from the CNV analysis. Also, tumor markers expression like Ptch1/2, Gli1, MYCN, etc.. should be shown in individual samples.
Of note, KC4 and KC5 express very low levels of KRT14, which suggests they are not proper keratinocytes.

Reviewer #4

(Remarks to the Author)
I thank the authors for their efforts to address the concerns that I raised in the initial review of the manuscript. However, these attempts have not sufficiently resolved these concerns and similar issues remain with the revised version, which still presents largely descriptive findings and multiple conclusions that are insufficiently justified. Furthermore, the novelty here is limited as the phenotypes and functions described have been described previously. Perhaps there is a case to be made that these phenotypes have not previously been defined in skin cancer, but given their similarity to other tumour types it is debatable whether these findings are likely to receive significant interest from the wide readership of Nat Comms and may be better suited in a more disease specific journal.

Nearly all statements from the abstract regarding results presented in this manuscript are still not adequately justified:
• “Matrix CAFs (mCAFs) ensheath tumor nests and synthesize extracellular-matrix to prevent T cell invasion.”
o This is a function that has been attributed to mCAFs in multiple studies so I don’t have too much difficulty accepting this conclusion. However, based on the data presented in this manuscript alone this is not convincingly shown. Figure 5D shows a slight reduction in CD3s within tumour nests where high CAFs or mCAFs are found. Therefore, this doesn’t demonstrate a specific role for mCAFs in limiting CD3+ cell accumulation in tumour nests. Furthermore, this is purely a correlative association and therefore insufficient to justify the statement above.

• “Immunomodulatory CAFs (iCAFs), which express proinflammatory and immunomodulatory factors, are only detected in high abundance in aggressive tumors.”
o The data presented shows that this is not the case. In fact, the new data provided in Figure S6A shows that there is no significant difference in iCAF abundance between the “aggressive” tumour subtypes compared to the earlier stages.
o The statistically significant difference shown in Figure 6B, is based on Chi squared analysis of multiple iCAFs vs no/single iCAFs. However, this Chi squared test approach to determining statistical significance is not sufficient to assess how consistently these differences are observed across biological replicates and therefore inappropriate to support the conclusion drawn. Additionally, the precise definition for what the categorical variable (multiple iCAFs vs no/single iCAFs) represents and how it was assigned is not clearly described.

• “Strikingly, iCAFs but not tumor cells are (apart from immune cells) the exclusive cell type producing chemokines and, thus, play a key role in immune cell recruitment and activation.”
o The role of iCAFs in immune cell recruitment to tumours has not been demonstrated or even analysed.
o The production of chemokines and cytokines has only been shown at the transcript level and is not confirmed to result in protein secretion, which would be critical to a functional role in immune cell recruitment and/or activation.
o The analysis of iCAF’s role in immune cell activation (Figure 8) is very limited and (to support the importance of the point made above) the only conditioned media treatment shown to generate fibroblasts that significantly increased CD4 activation was from VM26, which is the condition where very few of the iCAF genes were upregulated in Figure 6. Therefore, calling into question whether there is any link between these co-culture assays and the transcriptomic profile of the fibroblasts used or analysed in human tissue samples.
o This analysis also many technical limitations, *Further comments on this are provided below.

• "Mechanistically, we show that cancer cells transform adjacent healthy fibroblasts into cytokine-expressing iCAFs, which subsequently recruit immune cells and modulate the immune response."
o In addition to the comments above, there are unclear discrepancies between the mechanism proposed from in vitro experiments using conditioned media and the trajectory analysis performed using scRNA-seq data. Trajectory analysis shows mCAFs to precede iCAFs in the differentiation trajectory. So how do the authors reconcile this with the findings that cancer cell conditioned media induce iCAF markers but not mCAF markers? Is it possible that in the tumour

microenvironment an entirely different mechanism is active, involving immune cell mediated iCAF activation? Furthermore, the data provided to show that metastatic cell lines induce iCAFs and primary lines do not are inconsistent, as described above with VM26 CM not capable of inducing upregulation of iCAF marker genes.

* Activation of T-cells shown in Figure 7 has multiple limitations.
• This analysis should be conducted with positive and negative (Isotype/FMO) controls.
o Without showing these controls it can't be determined what degree of staining reflects activation.
o It is likely that the MFI measurements used for quantification in Figure 7B are not appropriate to analyse differences in activation, due to distinct positive (or bright) populations shown in the histograms. This non-gaussian distribution renders the MFI an inaccurate value for summarising the data.
o It should be assessed whether the bright population represents an active population using a positive control (e.g. CD3 and CD28 stimulation) and then each condition should be quantified as the percentage of CD4/CD8 that have been activated.
• The results statements accompanying this figure are not also adequately justified or simply incorrect.
o "VM15, VM26, VM19, VM25 were more potent to induce proliferation in CD4 or CD8 T cells than the corresponding untreated NHDFs and cancer cells alone (Figure 7A)."
 This is clearly not true for VM19 and VM25 and presumably not significant for VM15 and VM26.
o "Early (CD69) and late (CD45RO) activation markers were upregulated on CD4 and CD8 T cells after 24h or 96h when co-cultured with pretreated NHDFs"
 This is true compared to T-cells alone, but this is not the relevant comparison.
 Very few changes are significant when considering the comparison to untreated NHDFs (the appropriate comparison for the conclusions drawn).
• The figures seem to be cropped and lack axes labels.
• The legend is not sufficiently detailed – what does each point represent in these graphs?

Version 2:

Reviewer comments:

Reviewer #3

(Remarks to the Author)
The authors satisfactorily addressed my points.
Minor point:
The authors claim that comparable scRNAseq addressing fibroblast heterogeneity in skin cancer are missing or included few or no fibroblasts. I do not agree with this assumption. First they are some addressing CAF heterogeneity, including some non-cited (doi.org/10.1038/s41467-023-41141-9). Second, the number of CAF included in each dataset does not necessarily correlate with the quality of the dataset. The authors should either rephrase or show that their dataset highlight previously unidentified heterogeneity in skin CAF.

Reviewer #4

(Remarks to the Author)
I would like to begin by expressing my appreciation to the authors for their diligent efforts in revising the manuscript in response to previous comments. Your commitment to enhancing the clarity and quality of the research is commendable and reflects a strong dedication to advancing our understanding of this topic.
The revised manuscript now robustly demonstrates most of the points raised in earlier feedback. However, I have identified a few remaining discrepancies between the data presented and their description in the text. I believe addressing or clarifying these points prior to publication will further strengthen the manuscript's overall impact and scientific rigor.
Below, I outline these areas of concern, along with suggestions for potential improvements:

RE: Linear regression analysis – p10 lines 341-343.
"Of note, while total CAF and mCAF numbers negatively correlate with CD3 cells/mm² in tumor nests, iCAF numbers did not (Linear regression: total CAFs: R^2=0,039; mCAFs: R^2=0,040; iCAFs: R^2=0,009)"
These R^2 values suggest a modest correlation, indicating that mCAF abundance accounts for approximately 4% of the variance in CD3 accumulation within tumor nests. While this could potentially have a biological effect, it would be beneficial to provide further details on the strength and statistical significance of these correlations to better understand the implications.

RE: LegendPLEX validation (Figure S11) – p11 lines 391 – 393
"Importantly, we confirmed the expression of several cytokines and chemokines by fibroblasts and induced iCAFs on protein level with LEGENDplex assays (Figure S11)."
The results in Figure S11 appear to show that the increased expression levels observed in NHDFs treated with VM08 and VM15 conditioned media at the transcript level are not consistently reflected at the protein level. It would be helpful to revise this section of the text and relevant sections of the discussion to more accurately represent the LegendPLEX data, which clearly has implications for understanding the mechanism involved in these cell's role in the T-cell activation assays or alternate functions.

RE: T-cell activation by iCAFs (Figure 7) – p11 line 403-404
The statement on p11 lines 403-404 “This potential to activate T cells was enhanced when fibroblasts were exposed to the secretome of cancer cells”, could be refined to better reflect the heterogeneity observed across different cancer cell lines. While the subsequent sentences do elaborate on this variability, it may be more accurate to revise the initial statement to avoid potential misinterpretation.
Additionally, the interpretation of these data might be enhanced by more clearly connecting the qPCR results with the T-cell activation experiments. For instance, it could be noted that VM15 conditioned media, which consistently enhanced cytokine transcript levels in the qPCR experiments, also consistently increased T-cell proliferation and activation. This connection could help strengthen the overall narrative.
Lastly, it would be prudent to double-check the statistical analyses presented in Figure 7. In particular, the significance reported for Fig 7B (proliferating CD4s) between NHDFs and VM19 CM treated NHDFs may warrant review. It would also be helpful to clarify whether Welch's correction was applied in the unpaired Student's t-tests to account for unequal variances between groups. If this correction was not applied, the analyses should be updated accordingly.
I would also recommend that the authors clarify the expected role of T-cell activation in tumor progression, perhaps in the discussion. Given that iCAFs were associated with aggressive tumors, it is somewhat unexpected that their primary function would be to activate T-cells, which generally have a tumor-suppressive role.

Open Access This Peer Review File is licensed under a Creative Commons Attribution 4.0 International License, which permits use, sharing, adaptation, distribution and reproduction in any medium or format, as long as you give appropriate credit to the original author(s) and the source, provide a link to the Creative Commons license, and indicate if changes were made.
In cases where reviewers are anonymous, credit should be given to 'Anonymous Referee' and the source.
The images or other third party material in this Peer Review File are included in the article’s Creative Commons license, unless indicated otherwise in a credit line to the material. If material is not included in the article’s Creative Commons license and your intended use is not permitted by statutory regulation or exceeds the permitted use, you will need to obtain permission directly from the copyright holder.
To view a copy of this license, visit https://creativecommons.org/licenses/by/4.0/

Point-by-point response

We would like to thank the reviewers for their close engagement with our findings and their insightful comments on our manuscript. As seen below in our point-by-point response, the input from the reviewers has been very important. The questions and concerns have inspired us to go back and revisit key aspects (summarized below), which have led to important clarifications and overall a very satisfying outcome of this extensive revision.

Summary of additional data and analysis added to the revised manuscript

- We performed quantitative image analysis of CAF subpopulations in nodular/infiltrative BCC, well/poorly differentiated SCC and high/low grade melanoma (n = 36). This analysis confirms the correlation of iCAFs with increased tumor progression. Additionally, we added representative RNA-Scope co-stainings to show that iCAFs, mCAFs and RGS5$^+$ cells are independent populations (Figure S6A and S6B).
- We verified our cell-cell communication results with an independent analysis using *CellChat* (Figure 5F and S8B).
- We extended the representative images panels showing the distribution pattern of iCAFs and mCAFs *in situ* (Figure S5).
- We added a heatmap of transcription factors (TFs) that are differentially expressed in mCAFs and iCAFs. iCAFs are enriched in TF expression that are related to immune responses, and mCAFs are enriched in TFs related to the WNT pathway, mesenchymal cell lineages or anti-inflammatory signaling (Figure 3C).
- We verified our CAF subsets in publicly available datasets of CAFs/fibroblasts from HNSCC (Puram et al., 2020), cutaneous SCC (Ji et al., 2020) and BCC (Yerly et al., 2022), (Figure S10).
- We performed additional scoring of abnormal keratin expression in CNV negative cells of healthy *vs* neoplastic keratinocytes at second level clustering (Figure 2C and S2A).
- We repeated the *in vitro* conditioned media (CM) assays – tumor cell to fibroblast effect – with additional cell lines. These experiments confirm the upregulation of iCAF but not mCAF marker genes in normal healthy fibroblasts that were stimulated with CM from tumor cells (Figure 6C and S9). Notably, CM derived from metastatic melanoma cell lines or the SCC13 cell line stimulated upregulation of iCAF genes in fibroblasts stronger than CM derived from primary melanoma cell lines.
- We performed additional *in vitro* assays to investigate T cell activation induced by fibroblasts, which show that fibroblasts that were pretreated with CM from tumor cells activated T cells (Figure 7). We found increased proliferation, short (CD69$^+$) and long (CD45RO$^+$) term activation in CD4+ T cells as well as increased proliferation and short term (CD69$^+$) activation in CD8+ T cells that were co-cultured with pre-treated fibroblasts (Figure 7).

Summary of additional data and analysis for reviewers only

- Deconvolution of TCGA skin cutaneous melanoma bulk RNAseq data using CIBERSORTx shows that the abundance of iCAFs, but not mCAFs, correlate with advanced tumor staging (Figure REV1).
- Cell cycle scoring of healthy and neoplastic keratinocyte second level clustering (Figure REV2) shows an increased number of proliferating cells in keratinocyte cluster KC4.
- Local Inverse Simpson's Index (LISI) of batch-corrected and non-corrected clustering (Figure REV3).
- Co-stainings of three mCAF marker genes (*COL11A1*, *MMP11*, *PTGDS*) shows their co-expression in the same cells and verifies the presence of mCAFs *in situ* (Figure REV5).

Reviewer #1

This manuscript by Forsthuber and colleagues examine CAF types in the three types of skin cancer. The investigators show that mCAFs arise in early stage tumors and produce extra-cellular matrix to limit immunosurveillance. The authors also show that iCAFs are present primarily in aggressive lesions and produce chemokines regulating immune cell recruitment and activation.

This manuscript addresses a knowledge deficit regarding the molecular phenotype of fibroblasts in present in skin cancers in the TME. The data provide insights regarding potential regulatory functions for fibroblasts in regulating skin cancers. Therefore, the data are interesting and of value to the broader research community. However, there are a number of issues listed below that should be addressed.

We thank the reviewer for the supporting acknowledgement on our study on skin cancer fibroblasts being "of value for a broader research community" and for the insightful comments and suggestions how to further improve our work. We have addressed all questions as explained in detail in the point-by-point response below.

1. Intro, first line, Understudied is probably a more accurate term than Underestimated.
RE: Thank you for the note. We have now rephrased, better leading into the topic.

2. page 3, line 61-3, This ref is from 2006, much improvement in melanoma therapeutic outcomes has happened in the interval primarily involving use of immunomodulatory therapies. Poorer outcomes for metastatic diseases were typical prior to 2006 but significantly better now. Perhaps this reality could be worked into the discussion.

RE: We have added more recent references, and added a sentence about improved clinical outcomes for melanoma patients with immunotherapies.

Curti, Brendan D., and Mark B. Faries. "Recent Advances in the Treatment of Melanoma." Edited by Dan L. Longo. New England Journal of Medicine 384, no. 23 (June 10, 2021): 2229–40. https://doi.org/10.1056/NEJMra2034861.
Davis, Lauren E., Sara C. Shalin, and Alan J. Tackett. "Current State of Melanoma Diagnosis and Treatment." Cancer Biology & Therapy 20, no. 11 (November 2, 2019): 1366–79. https://doi.org/10.1080/15384047.2019.1640032.

3. The terminology used in Sup Table 1 needs clarification.
a) The BCCs analyzed should be classified regarding their histologic type, superficial multicentric, nodular, infiltrative, morpheaform, etc.
RE: All BCC samples are nodular BCCs, which has been updated in Supp. Table 1, following the terminology of Fernández-Figueras et al., 2022 (BCC of nodular, infiltrative, superficial, basosquamous, infundibulocystic and fibroepithelial subtype, and BCC with sarcomatous differentiation).

Fernández-Figueras, M.T., Malvehi, J., Tschandl, P., Rutten, A., Rongioletti, F., Requena, L., Kittler, H., Kerl, K., Kazakov, D., Cribier, B., Calonje, E., André, J., Kempf, W. and (2022), Position paper on a simplified histopathological classification of basal cell carcinoma: results of the European Consensus Project. J Eur Acad Dermatol Venereol, 36: 351-359. https://doi.org/10.1111/jdv.17849

b) What is "Bowen carcinoma". Typically Bowen's disease in the skin indicates a form of

squamous cell carcinoma in situ. Do you mean squamous cell carcinoma arising from a pre-existing Bowen's disease lesion? If it is CIS probably best to call it Bowen's Disease.
RE: Thank you for the comment. Indeed, SCC IV is an SCC arisen from Bowens Disease, which we have updated in Supp. Table1.
c) Instead of highly differentiated, better to say 'well differentiated'.
RE: We have changed the terminology in Supp. Table 1 to "well differentiated" and "poorly differentiated", following the terminology of the European consensus-based interdisciplinary guideline (well differentiated, moderately differentiated, poorly differentiated, undifferentiated) (Stratigos et al., 2015)

Stratigos A, Garbe C, Lebbe C, Malvehy J, del Marmol V, Pehamberger H, Peris K, Becker JC, Zalaudek I, Saiag P, Middleton MR, Bastholt L, Testori A, Grob JJ; European Dermatology Forum (EDF); European Association of Dermato-Oncology (EADO); European Organization for Research and Treatment of Cancer (EORTC). Diagnosis and treatment of invasive squamous cell carcinoma of the skin: European consensus-based interdisciplinary guideline. Eur J Cancer. 2015 Sep;51(14):1989-2007. doi: 10.1016/j.ejca.2015.06.110. Epub 2015 Jul 25. PMID: 26219687.

4. Can the authors address why they felt not matching the healthy skin samples with the tumor samples for site and sex is appropriate? There is a strong female bias in the control samples.
RE: We agree with the reviewer that there is a strong female bias in the healthy skin samples. This is in part because plastic surgery corrections of not sun-exposed skin are mostly performed in females, and in part because these surgeries were rare during the COVID pandemic. Although we collected more female tumor samples initially, some of them did not pass the quality control before sequencing, and had to be excluded. However, from the majority of donors, non-affected (healthy) skin adjacent to the respective tumors was included in the sequencing dataset, resulting in sex- and site-matched skin samples.

5. The overall cell number sequenced from the tumor and skin samples seems low. Was this the experimental strategy?
RE: Instead of random droplet-based sampling of tumor-associated cells, we chose a FACS-sorting approach to (i) enrich for fibroblasts and (ii) to gain highly sensitive scRNA-seq data using Smart-seq2 technology. From previously published scRNA-seq skin data generated by 10x Genomics, we knew that the well-known fibroblast marker *PDGFRA* is expressed below detections limit (eg. Tabib et al. 2018). Using Smart-seq2 we were able to overcome this issue. With our enrichment strategy we were able to gain 2239 fibroblasts (including 1216 CAFs) after QC filtering, and robustly identified CAF heterogeneity from n=10 donors. Previous skin cancer studies included: Tirosh et al., Science 2016: 19 melanomas, 4,645 cells, 61 CAFs; Jerby-Arnon et al., Cell 2018: 33 melanomas, 7,186 cells, 106 CAFs; Ji et al. Cell 2020: 10 SCC, 48,000 cells, 882 CAFs (>50% of CAFs from a single patient); Guerrero-Juarez et al., Science Advances 2022: 4 BCC, 36,392 cells, 5,775 fibroblasts (CAF number was not specified) corresponding to only 2 donors (**Supplementary Table 1**). The low number of fibroblasts in these previous studies as well as our pilot experiments led to the decision that enrichment for fibroblasts is essential to receive high quality CAF data.

Tabib, Tracy, Christina Morse, Ting Wang, Wei Chen, and Robert Lafyatis. "SFRP2/DPP4 and FMO1/LSP1 Define Major Fibroblast Populations in Human Skin." Journal of Investigative Dermatology 138, no. 4 (April 2018): 802–10. https://doi.org/10.1016/j.jid.2017.09.045.
Tirosh, Itay, Benjamin Izar, Sanjay M. Prakadan, Marc H. Wadsworth, Daniel Treacy, John J. Trombetta, Asaf Rotem, et al. "Dissecting the Multicellular Ecosystem of Metastatic Melanoma by Single-Cell RNA-Seq." Science 352, no. 6282 (April 8, 2016): 189–96. https://doi.org/10.1126/science.aad0501.

Jerby-Arnon, Livnat, Parin Shah, Michael S. Cuoco, Christopher Rodman, Mei-Ju Su, Johannes C. Melms, Rachel Leeson, et al. "A Cancer Cell Program Promotes T Cell Exclusion and Resistance to Checkpoint Blockade." Cell 175, no. 4 (November 2018): 984-997.e24. https://doi.org/10.1016/j.cell.2018.09.006.
Ji, Andrew L., Adam J. Rubin, Kim Thrane, Sizun Jiang, David L. Reynolds, Robin M. Meyers, Margaret G. Guo, et al. "Multimodal Analysis of Composition and Spatial Architecture in Human Squamous Cell Carcinoma." Cell 182, no. 2 (July 2020): 497-514.e22. https://doi.org/10.1016/j.cell.2020.05.039
Guerrero-Juarez, Christian F., Gun Ho Lee, Yingzi Liu, Shuxiong Wang, Matthew Karikomi, Yutong Sha, Rachel Y. Chow, et al. "Single-Cell Analysis of Human Basal Cell Carcinoma Reveals Novel Regulators of Tumor Growth and the Tumor Microenvironment." Science Advances 8, no. 23 (June 10, 2022): eabm7981. https://doi.org/10.1126/sciadv.abm7981.
Yerly, L.; Pich-Bavastro, C.; Di Domizio, J.; Wyss, T.; Tissot-Renaud, S.; Cangkrama, M.; Gilliet, M.; Werner, S.; Kuonen, F. Integrated Multi-Omics Reveals Cellular and Molecular Interactions Governing the Invasive Niche of Basal Cell Carcinoma. Nat Commun 2022, 13 (1), 4897. https://doi.org/10.1038/s41467-022-32670-w.

6. Fig 6D, the terminology used is confusing. What is a non-invasive BCC, differentiated SCC, SSM? By definition, these lesions invade the dermis.
RE: The terminology in **Figure 6D** has been changed to poorly and well differentiated SCC, nodular and infiltrative BCC, as well as low and high grade melanoma, following consensus-based terminology (Stratigos et al., 2015; Fernández-Figueras et al., 2022, Keung et al., 2018)

Keung, Emily Z., and Jeffrey E. Gershenwald. "The Eighth Edition American Joint Committee on Cancer (AJCC) Melanoma Staging System: Implications for Melanoma Treatment and Care." Expert Review of Anticancer Therapy 18, no. 8 (August 3, 2018): 775–84. https://doi.org/10.1080/14737140.2018.1489246.

7. line 318, none of the tumors analyzed are benign as listed in 6b, they are various types of carcinomas or melanoma with varying potentials to metastasize and/or spread locally. So it is confusing to describe a subset as benign. Better to say low-grade for lesions that have a low probability of metastasis and high-grade for lesions that with a higher probability of metastasis.
RE: We thank the reviewer for pointing this out. We have rephrased the paragraph.

8. Were there any differences in the transcription factor expression profiles between the iCAFs and mCAFs?
RE: We thank the reviewer for this suggestion, which we followed up with additional analysis. Yes, we see differences in the expression of transcription factors (TFs) between iCAFs and mCAFs, which we now included as **Figure 3C** in the manuscript. mCAFs are enriched for example in TF expression of the WNT pathway (*CXXC5*, *TCF4*), TFs known for mesenchymal cell lineages (*TWIST1*, *TWIST2*), and anti-inflammatory signaling (*KCNIP3*). iCAFs, are enriched in TF expression that are related to immune responses (e.g.: *STAT1*, *IRF1*, *IRF9* or *ARID5A)*. Interestingly, there is also a difference in TF expression between BCC-, SCC- or melanoma-derived iCAFs.

Additional text in methods: Differentially expressed genes between iCAFs and mCAFs were calculated using default parameters for FindMarkers(). To query for differentially expressed transcription factors among the significantly differentially expressed genes (adjusted p value < 0.05), we used the human transcription factor list from Lambert et al. 2018.

Lambert, Samuel A., Arttu Jolma, Laura F. Campitelli, Pratyush K. Das, Yimeng Yin, Mihai Albu, Xiaoting Chen, Jussi Taipale, Timothy R. Hughes, and Matthew T. Weirauch. "The Human Transcription Factors." Cell 172, no. 4 (February 2018): 650–65. https://doi.org/10.1016/j.cell.2018.01.029.

Reviewer #2

In this paper, Forsthuber et al., presented a single-cell RNA-seq study of sorted cell populations including keratinocytes, fibroblasts and immune cells across three skin cancer types, melanoma, BCC and SCC, using smart-seq2, with a focus on the intra-tumoral heterogeneity of CAFs. They identified three main CAF types, mCAF, iCAF and RGS5+ CAFs, and found mCAFs were more abundant in early-stage tumors, while iCAF were more enriched in aggressive tumors. These findings were further validated by RNAscope and IHC in 39 tumors. Overall, the manuscript is well written. It provides valuable resources to the skin cancer research community.

However, the whole paper felt a bit descriptive in some places (although this is often due to the nature of scRNA-seq studies), and it needs more statistical power and analysis to make the findings robust. My specific comments are as follows,

We appreciate that this reviewer thinks that our manuscript "provides valuable resources to the skin cancer research community", and thank the reviewer for the valuable feedback. We have addressed all questions and suggestions, which further improved our manuscript and strengthened our findings.

1. Quantification of RNAscope and IHC. Although the study performed RNAscope and IHC in situ validation of some top markers in 39 tumors, the results were just descriptive with representative images shown. Can the authors quantify all the staining data, and compare them between cancer types and stages? Then the proper statistics can be derived. This relates main figures 4-6.
RE: We have performed computer-assisted quantification of *RGS5*$^+$ CAFs, mCAFs and iCAFs in 36 tumors with HALO (digital pathology) software followed by statistical analysis. We have stratified the samples according to different malignancy stages into nodular versus infiltrative BCC, poorly and well differentiated SCC, and early- and late-stage melanoma. The quantification confirmed the correlation of iCAFs with increased tumor progression (**Figure S6A**).

2. Clinical inference and importance. Although authors included different cancer types and various stages, the numbers of samples were too low to derive robust clinical inference. Using various published and publicly available bulk RNA-seq data sets of melanoma, BCC and SCC, can authors perform bulk tissue deconvolution based on their identified signatures, and then test
- if the difference of various CAFs among different cancer types can be validated using bulk RNA-seq as well
- if the abundance and intensity of various CAFs are associated with important clinical outcome, e.g., survival/metastasis in melanoma, in situ vs. invasive SCC, metastasizing vs non-metastasizing tumours?

RE: To our knowledge, skin tumor RNA-seq datasets that also include clinical data are available for melanoma only (TCGA skin cutaneous melanoma dataset). There are datasets for BCC and SCC on TCGA, however, they only contain genomic, but no transcriptomic data.
Using CIBERSORTx on the TCGA skin cutaneous melanoma dataset we found that the abundance of iCAFs, but not mCAFs, correlated with advanced tumor staging (tumor staging according to AJCC pathologic tumor staging) (**Figure REV1**), which confirms our findings shown in **Figure 6B** and **S6A**. Moreover, the CIBERSORT analysis also shows that samples with a higher fraction of iCAFs contain a lower fraction of mCAFs (**Figure REV1**).

Additional text in methods: We applied the deconvolution tool CIBERSORTx from the Alizadeh Lab and Newman Lab (https://cibersortx.stanford.edu, Newman et al.2019) on the TCGA skin cutaneous melanoma dataset. As the input data we used our scRNAseq expression matrix containing pFIB, rFIB, iCAFs, mCAFs, *RGS5*$^{+}$ and vSMC cells and their TOP50 DEGs from which CIBERSORTx calculated gene expression signatures. These signatures were further used to query for cell fractions in the bulk RNA-seq TCGA dataset. Results were linked to clinical data of tumor stages as well as presence and stage of lymph node and distant metastasis. For correlations of T (tumor), N (lymph node metastasis) and M (distant metastasis) stages to the abundance of iCAFs or mCAFs we distinguished between tumors with no iCAFs and tumors harboring iCAFs among all tumor-associated cells, and for mCAFs the cutoff was set to the median, respectively.

Newman, Aaron M., Chloé B. Steen, Chih Long Liu, Andrew J. Gentles, Aadel A. Chaudhuri, Florian Scherer, Michael S. Khodadoust, et al. "Determining Cell Type Abundance and Expression from Bulk Tissues with Digital Cytometry." Nature Biotechnology 37, no. 7 (July 2019): 773–82. https://doi.org/10.1038/s41587-019-0114-2.

3. Cell-cell communication. In figure 5, the author performed the receptor ligand analysis using the scRNA-seq data. Can authors use some commonly used cell-cell communication tools, such as cellphoneDB, cellChat, Celltalker, to validate all the cell-cell communication results?
RE: We used CellChat to validate our results and included representative communication plots in **Figure 5F** and **Figure S8B**. The CellChat analysis also highlights the unique immune-signaling properties of iCAFs compared to mCAFs (**Figure 5F**).

Additional text in methods:
Additionally, we verified the receptor-ligand interactions with CellChat (Jin et al., 2021). The communication probability was calculated according to default parameters. We present selected receptor-ligand pairs as circular plots using the function netVisual individual(source.use = c("mCAF", "iCAF"), layout = chord).

Jin, Suoqin, Christian F. Guerrero-Juarez, Lihua Zhang, Ivan Chang, Raul Ramos, Chen-Hsiang Kuan, Peggy Myung, Maksim V. Plikus, and Qing Nie. "Inference and Analysis of Cell-Cell Communication Using CellChat." Nature Communications 12, no. 1 (February 17, 2021): 1088. https://doi.org/10.1038/s41467-021-21246-9.

4. Trajectory. Can authors perform the trajectory analysis on fibroblasts to further study the developmental trajectory of fibroblasts. It may give some clue of the origin of CAFs.
RE: We thank the reviewer for this suggestion. We used Monocle 2 (Qiu et al., 2017) and Monocle 3 (Cao et al., 2019) to analyze the developmental trajectory of fibroblasts and included the results as new **Figure 3D** and **3E**. The trajectory analyses revealed two differentiation paths from healthy fibroblasts: either towards mCAF/iCAF or toward *RGS5*$^{+}$cells. The analysis also shows that iCAFs are a differentiation endpoint, with mCAFs being an intermediate state, and thus it may be possible that iCAFs develop from mCAFs. At current, this remains an open possibility which needs to be confirmed (or refuted) with future in-depth experiments. Interestingly, adjacent skin cells are preferentially found in the healthy fibroblast branch or the RGS5^{+} cell branch, and a small fraction in the mCAF/iCAF branch, which indicates that they are in a transitory position between healthy fibroblasts and CAFs.

Additional text in methods:
We used Monocle2 (v2.28, R4.0.0) (Qiu et al., 2017) and Monocle3 (v0.2.1, R3.6.2) (Cao et al., 2019) to perform trajectory analysis. For both methods, we extracted RPKM data, phenotype data, and feature data from the Seurat object (second-level clustering of fibroblasts without vSMC) from which we created a newCellDataSet(lowerDetectionLimit = 0.1, expressionFamily = tobit()) or a new_cell_data_set() object using default parameters.
For Monocle2, we converted our RPKM data into mRNA counts using relative2abs() and generated the NewCellDataSet(lowerDetectionLimit = 0.5, expressionFamily = negbinomial.size()) object again. As quality filtering and clustering were already performed in Seurat, we directly constructed single cell trajectories using all significantly (adjusted p-value < 0.01) regulated DEGs (FindMarkers()) as input parameters for ordering cells. For calculating pseudotime, we used healthy skin cells from controls as our starting point. Cells were plotted using plot_cell_trajectory() colored by “clusters”, “category” and “pseudotime”.
For Monocle 3, we manually added clusters, UMAP and PCA parameters to the new_cell_data_set() object and calculated the trajectory graph with learn_graph(object, use_partition = F). For calculating pseudotime we used healthy skin clusters (pFIB and rFIB) as root_cells and used plot_cells(color_cells_by = "pseudotime") to present the data.

Qiu, Xiaojie, Qi Mao, Ying Tang, Li Wang, Raghav Chawla, Hannah A Pliner, and Cole Trapnell. “Reversed Graph Embedding Resolves Complex Single-Cell Trajectories.” Nature Methods 14, no. 10 (October 2017): 979–82. https://doi.org/10.1038/nmeth.4402.
Cao, Junyue, Malte Spielmann, Xiaojie Qiu, Xingfan Huang, Daniel M. Ibrahim, Andrew J. Hill, Fan Zhang, et al. “The Single-Cell Transcriptional Landscape of Mammalian Organogenesis.” Nature 566, no. 7745 (February 28, 2019): 496–502. https://doi.org/10.1038/s41586-019-0969-x.

Some minor points

5.) Line 288-290. “iCAFs….. are an exclusive source of many chemokines (Figure 5D).”. How was this analysis done? What is the evidence to support “being exclusive”?
RE: Thank you for pointing this out. Indeed, apart from CAFs also immune cells express cytokines and chemokines which is expected and thus we rephrased the paragraph accordingly. Importantly, we overall want to highlight that CAFs but not the tumor cells *per se* are the major source of immunomodulatory factors, which underlines their role in immune cell recruitment and immune surveillance.

6.) Figure 6B. Could statistics be done on figure 6b to identify significance?
RE: We calculated the statistics with a Chi-square test between the corresponding groups and added it to **Figure 6B**.

7.) Line 311-312. “….resulted in a proliferative and activated iCAF-like phenotype in vitro (Figure 6C).” where is the data supporting “proliferative”? I cannot see it.
RE: Thank you. Figure 6C indeed did not show proliferation and we updated this sentence.

Reviewer #3

The manuscript integrates scRNAseq and spatial RNA FISH staining from human BCC, SCC and melanoma to decipher the commonalities and specificities of CAF heterogeneity. The authors identifiy 3 cancer associated CAFs phenotypes, RGS5+, iCAF and mCAF. mCAF are defined by high expression of matrix-associated genes and mostly found in BCC and SCC, while iCAF are defined by the expression of immunomodulatory genes, and preferentially found in invasive melanoma and poorly-differentiated SCC. A panel of immunomodulatory cytokines is induced by treatment of human dermal fibroblasts with the conditioned medium of melanoma and SCC cell lines. The manuscript provides a deep molecular single-cell and spatial characterization of CAFs heterogeneity in skin cancer. However, in our opinion, lacks clear identification of tumor cells and requires stronger validation using independent scRNAseq cohorts and additional stainings.

We thank the Reviewer for the valuable feedback and suggestions. We have addressed their points of criticism in detail as outlined in the point-by-point response below.

Major comments:

1.) The authors claim that the unsupervised clustering separated healthy and malignant keratinocytes (line 90 and line 96). However, the authors do not show how they discriminate normal neoplastic keratinocytes from normal keratinocytes, which are expected to be mixed in BCC and SCC samples. Similarly, the authors do not show how they discriminate neoplastic from normal melanocytes coming from melanoma samples. Indeed, the CNV analysis shown in Fig 2C and Supp 2A shows high intra-sample heterogeneity, with high proportions of CNV negative cells. Samples like BCCI, BCCII, BCCIII, SCCI and SCCII have indeed very low number of CNV positive cells. How do the authors make sure they have indeed tumor cells in significant proportions in theses samples? This important concern largely precludes from further interpretation of the data.

RE: We disagree with the Reviewer that the computational analysis "lacks clear identification of tumor cells". As explained in the main text and the methods section in detail, we have used CNV analysis to identify malignant keratinocytes and neoplastic melanocytes in the tumor samples, similar to Puram et al. 2018 and many other publications thereafter. This well-known computational method (inferCNV) detects longer genomic stands of CNVs based on RNA data, but is not powerful enough to detect point mutations or smaller genomic rearrangements of earlier cancer stages. Of note, in **Figure 2C** all cells of each tumor sample have been plotted including endothelial cells, immune cells and fibroblasts, which usually lack larger chromosome arrangements. As stated by this Reviewer, the keratinocytes in some BCC and SCC samples did not contain obvious chromosome rearrangements compared to the neoplastic melanocytes in the melanoma samples. Therefore, we used enhanced expression of *PTCH1* and *PTCH2* to identify malignant keratinocytes within the BCC samples (**Figure 2A-C and S2A-C**). This method was also used by Yerly et al. 2022 in their BCC dataset published in Nature Communications in 2022. For the SCC samples, we could confirm the expression of atypical keratins including *KRT6A*, *KRT7*, *KRT8*, *KRT15*, *KRT16*, *KRT18* and *KRT19* in malignant keratinocytes (**Rebuttal Figure R1**), as outlined in the main manuscript text in more detail. Healthy keratinocytes expressed the well-known basal and suprabasal keratins *KRT5*, *KRT14*, and *KRT1* and *KRT10*, respectively. We calculated a score from the expression of atypical keratins that discriminates healthy from neoplastic epithelial cells. We have included additional Violin plots (**Figure S2A**) to show that our analysis and identification of tumor cells is indeed robust.

Yerly, Laura, Christine Pich-Bavastro, Jeremy Di Domizio, Tania Wyss, Stéphanie Tissot-Renaud, Michael Cangkrama, Michel Gilliet, Sabine Werner, and François Kuonen. "Integrated Multi-Omics Reveals Cellular and Molecular Interactions Governing the Invasive Niche of Basal Cell Carcinoma." Nature Communications 13, no. 1 (August 20, 2022): 4897. https://doi.org/10.1038/s41467-022-32670-w.

2.) Do the authors identify a cycling cell cluster?
RE: Cell cycle scoring showed that KC1, a cluster in which SCC cells with CNVs are clustering (Figure 2C and S2B), is a proliferating cell cluster (Response **Figure REV2a,b**). KC4, in which cells from BCC, SCC, melanoma as well as healthy skin clustered together, contains a larger fraction of proliferating cells. Additionally KC4 shows elevated expression of *KI67* (Response **Figure REV2a,c**).

3.) The authors claim that single-cell studies on human skin cancer included only a few or no fibroblasts (line 36). This is clearly untrue, as we found existing scRNAseq with significant fibroblast coverage for SCC (DOI: 10.1016/j.cell.2020.05.039) and BCC (DOI: 10.1126/sciadv.abm79; doi.org/10.1038/s41467-022-32670-w), with the last two aiming at deciphering fibroblast heterogeneity.
RE: First, we would like to note that we have sequenced 10 tumor samples in our study, which is more than most other published studies in skin cancer or other cancer types comprise. As outlined in the response to Reviewer 1(#5), we used the more sensitive Smart-seq2 method to enrich for fibroblasts which gave us higher resolution than any study so far published. To bring out this aspect clearer, we have now added a table **(Supplementary Table 1)**, which summarizes the number of fibroblasts in published scRNA-seq experiments in skin cancer. This table shows that our dataset of 15 samples (healthy & tumor tissue) with 2239 fibroblasts /1216 CAFs is the dataset with the highest number of fibroblasts from different donors. The table also highlights that even if a larger number of samples has been sequenced, not all patient samples contributed to the fibroblast clusters. For example, looking at the Ji et al. dataset, 10 SCC samples have been sequenced but less than 2% of the sequenced cells are fibroblasts, and out of those few cells more than 80% have been contributed by a single patient. In our dataset, every donor sample (n=15) contributed fibroblasts. We added a bar plot of cell numbers per donor contributing to the fibroblast second-level clustering to Figure S3 **(Figure S3B)**. In support of our CAF results, we reanalyzed the fibroblast subsets of the SCC and BCC datasets from Ji et al., 2020 and Yerly et al. 2022 **(Figure S10)**, which confirmed the separation of CAFs into clusters expressing iCAF, mCAF and RGS5$^+$ cell marker genes.

4.) The CAF cluster definition identified by the authors (RGS5+, mCAF and iCAF) should be analyzed in the light of and validated in the previously published independent scRNA-seq cohorts.
RE: This was a great suggestion. As mentioned above, we have re-analyzed the CAF populations of previously published scRNA-seq datasets (Puram et al., 2018 (HNSCC; oral SCC); Ji et al., 2020 (cSCC); Yerly et al., 2022 (BCC)) in respect to our iCAF, mCAF and RGS5$^+$ CAF marker gene expression **(Figure S10A-C)** showing agreement in the most relevant cSCC and BCC data.

Puram, Sidharth V., Itay Tirosh, Anuraag S. Parikh, Anoop P. Patel, Keren Yizhak, Shawn Gillespie, Christopher Rodman, et al. "Single-Cell Transcriptomic Analysis of Primary and Metastatic Tumor Ecosystems in Head and Neck Cancer." Cell 171, no. 7 (December 2017): 1611-1624.e24. https://doi.org/10.1016/j.cell.2017.10.044.
Ji, Andrew L., Adam J. Rubin, Kim Thrane, Sizun Jiang, David L. Reynolds, Robin M. Meyers, Margaret G. Guo, et al. "Multimodal Analysis of Composition and Spatial Architecture in Human Squamous Cell Carcinoma." Cell 182, no. 2 (July 2020): 497-514.e22. https://doi.org/10.1016/j.cell.2020.05.039.

5.) The authors should perform co-staining of mCAF and iCAF markers on BCC, SCC and melanoma, to show that they highlight distinct spatial clusters. They should show PTGDS and MMP1 expression on scRNAseq Fibroblast clusters.
RE: We have now included representative images of co-stainings of mCAF, iCAF and RGS5^{+} cells to our manuscript (**Figure S6B**), which show exclusive and specific staining of the respective CAF-subtype marker genes. Notably, the CAF subsets can either spatially inter-mingle (salt and pepper pattern) or cluster in more confined regions. As suggested by the reviewer, we show the respective expression of genes in the UMAPs for direct comparison above each tissue staining a smaller UMAP (**Figure S6B,** and **Figures 4–6**) including the expression of *PTGDS* and *MMP1* in **Figure 5A and 5B**.

6.) Line 268, the authors mention the infiltrative part of a Bowen, which are, by definition, non-invasive.
RE: Thank you for this note. Indeed, after consulting with our dermatopathologist, we have clarified that SCCIV is an SCC arisen from a Bowen Carcinoma. We note this now in Supplementary Table 2 and apologize for having been imprecise here.

The authors claim that mCAF are associated with low cancer aggressiveness, while iCAF are associated with more aggressive tumors, based on scRNAseq and stainings. This assumption requires quantifications of mCAF and iCAF stainings, including additional samples with representative stages/morphological pattern of progression for each subtype. Related to this, iCAF in situ quantification method in Fig6B is unclear.
RE: In response to this comment, we have performed computer-assisted quantification of mCAF, iCAF and *RGS5*$^{+}$ cells populations in 36 tumor samples of BCC, SCC and melanoma (representative stainings shown in **Figure S6B**) using the digital pathology HALO software **(Figure S6A)**. We have stratified the samples according to different malignancy stages into (i) nodular versus infiltrative BCC, (ii) poorly and well differentiated SCC, and (iii) low (Tis) and high-grade (> T3) melanoma. Although the differences are not significant due to the variability within and among samples, there is a clear trend of increased iCAF numbers in more aggressive tumors **(Figure S6A)**. In response to Reviewer 2's suggestion (#2), we also performed deconvolution on the TCGA skin cutaneous melanoma bulk RNA-seq data to query for our CAF subsets and correlated their presence to clinical outcome. Using CIBERSORTx as a deconvolution tool (Newman et al. 2019) we found a correlation of tumor stage and the percentage of iCAFs in the tumor **(Figure REV1)**. Additionally, the percentage of iCAFs negatively correlated with the percentage of mCAFs (**Figure REV1**).
Related to the last comment, we have included additional details to the methodology section how **Figure 6B** was quantified.

Newman, Aaron M., Chloé B. Steen, Chih Long Liu, Andrew J. Gentles, Aadel A. Chaudhuri, Florian Scherer, Michael S. Khodadoust, et al. "Determining Cell Type Abundance and Expression from Bulk Tissues with Digital Cytometry." Nature Biotechnology 37, no. 7 (July 2019): 773–82. https://doi.org/10.1038/s41587-019-0114-2.

7.) The authors claim that iCAF are the exclusive source of many chemokines (line 289). However their analysis does not include immune cells or endothelial cells, which are very probably involved in immunomodulation of the tumor stroma too (Fig. 5D).
RE: Thank you for pointing this out. Indeed, apart from CAFs also immune cells express cytokines and chemokines (endothelial cells were to sparce for robust analysis) and we rephrased the paragraph accordingly. Importantly, we overall want to highlight in this section

that CAFs **but not the tumor cells *per se*** are a major source of immunomodulatory factors, which underlines their role in immune cell recruitment and immune surveillance.

8.) In Fig. 6C, the authors test the effect of cancer cell lines conditioned medium on the expression of cytokines in NHDF. How are the cytokine measured, by qRT-PCR? By ELISA? How do the authors make sure that the observed inductions are not contamination by CM, as suggested by the similar expression patterns in stimulated CM and cancer cells (left and right parts of the graphs)?
RE: The cytokine expression was measured by qRT-PCR, thus a contamination from the conditioned medium (CM) is unlikely. Results were normalized to CM from control normal healthy dermal fibroblasts (NHDF). In the left part of the graph we show the expression of cytokines in NHDF that were treated with CM, and in the right part we show the expression in cancer cells only. Several chemokines that are not expressed in cancer cells themselves are induced in NHDFs upon treatment with CM.

9.) The authors claim that only metastatic melanoma and aggressive SCC lines induced iCAF cytokine expression. However, they previously found iCAF surrounding primary melanoma. They should test additional cell lines with various grades of aggressiveness. The authors should include similar experiments regarding mCAF markers.
RE: We included additional experiments, one with a metastatic and one with a primary melanoma cell line (now counting in total 3 metastatic and 2 primary melanoma cell lines, and one cutaneous SCC cell line). Conditioned media of two metastatic cell lines (VM08 and VM15) and the SCC cell line induced cytokine expression of all tested cytokines in NHDFs. Conditioned medium of VM26, a metastatic melanoma cell line, did not induce cytokine expression in NHDFs **(Figure 6C)**. However, like NHDFs treated with conditioned medium from all other melanoma and cSCC cell lines, also NHDFs that were pretreated with conditioned medium from VM26 were capable of activating T cells **(Figure 7)**. We also assessed if the conditioned media from different cell lines induced genes expressed by mCAF, which was not the case **(Figure S9A).** Thus, we conclude that these cell lines can induce an iCAF phenotype but not a mCAF phenotype *in vitro*.

Minor points:
Line 68: The mCAFs cluster is not new (10.3389/fmolb.2022.864302)
While it has been described that CAFs are major producers of matrix-associated genes in skin cancer (and other cancer types), it has not been shown that there is a specific subset of CAFs being the major source for produced matrix-associated genes. Please note, our mCAF (matrix CAFs) are not be confused with the previously described myoCAFs, which our study has resolved as a subset of the RGS5^{+} cluster.

Figure S1: Number of cells/sample should be added
RE: We added a bar graph showing number of cells/sample in **Figure S1D**.

Figure S2: In the text, Figure S2a appears after S2b and S2c
RE: This has been corrected.

Fig 3a: Unclassifiable CAF could be doublets or low quality cells. Please check.
RE: This is what we also thought at first. However, as we excluded doublets and low-quality cells in our filtering process and these cells remained, we kept them in the UMAPs but do not consider them further in detail which we note in the methods.

Line 239: S4C instead of S5C

RE: This has been corrected.

In Fig 5 a/b, S5a/b/c/d, adding a marker of tumor cells would help the interpretation
RE: Unfortunately, it was not possible to add another fluorophore to additionally stain for an epithelial or melanoma maker. We have demarcated the border of tumor nests and stroma in BCC and SCC, which is clearly visible and has been confirmed with the respective H&E stainings by a dermatopathologist.

Reviewer #4

This study from Forsthuber et al. attempts to uncover the role of fibroblasts in skin cancer progression using scRNA-seq, RNAscope/FISH and IHC analysis of human tissue samples. In general, the manuscript is clearly written and accurately cites relevant literature to highlight key areas of unmet need in this area of research. The figures are also produced to a very high standard. However, these data are too preliminary/descriptive to substantiate the conclusions drawn and fail to provide novel insight into fibroblast heterogeneity beyond what has been described in previous studies.

General comments on the manuscript's limitations: the experiments are not appropriately designed to accurately address the questions under investigation. For example, the scRNA-seq cohort is very small and heterogeneous; the RNAscope/IHC analysis does not provide sufficient markers (within individual multiplexed panels) to enable accurate differentiation between the cell subpopulations identified by scRNA-seq; very few (or arguably no) findings presented in the manuscript are supported by quantitative, statistically significant differences observed across biological replicates.

We thank this reviewer for pointing out that our manuscript is clearly written, and the figures are produced to a very high standard. We, however, respectfully disagree with the reviewer's notion that our findings fail to provide novel insight into fibroblast heterogeneity. In order to further underpin our findings, we have performed a number of additional experiments, which are summarized in detail at the beginning of the point-by-point response letter, here stated in brief (1-6), and below answered in depth.

Comments related to specific sections of the manuscript are provided below:

Abstract:
Many points made in the abstract are not adequately supported by the data presented
• "We show that two out of three CAF subtypes contribute to tumor immune surveillance with distinct mechanisms"
o From the data presented it is not shown whether CAF subtypes contribute to tumour immune surveillance, and it is certainly not shown whether this varies by subtype. Cytokine and ligand/receptor expression is shown at the mRNA level. How this impacts immune surveillance is suggested as a result of these data and previous studies. However, neither of these are sufficient to justify this claim and demonstrate distinct mechanisms of immune surveillance in skin cancer.
• "Matrix CAFs (mCAFs), a previously unknown subtype present in early-stage tumors, ensheath tumor nests and synthesize extracellular-matrix to prevent T cell invasion."
o This fibroblast subtype has been described in many studies of different cancer types. There is no statistically significant evidence provided to show that these cells are more abundant in early-stage tumours and this cannot be assessed in such a small and heterogeneous cohort. There is also no evidence shown that the ECM production regulated by these cells prevents T-cell infiltration, this is based on findings from other studies and not demonstrated here for skin cancer.
• "Immuno CAFs (iCAFs), which express proinflammatory and immunomodulatory factors, are only detected in high abundance in aggressive tumors."
o The term iCAF is typically used to describe inflammatory CAFs (not immuno CAFs). As above there is no statistically significant evidence provided to show these are only found in aggressive tumours.

• Mechanistically, we show that cancer cells transform adjacent healthy fibroblasts into cytokine-expressing iCAFs, which subsequently recruit immune cells and modulate the immune response.
o The molecular mechanism of fibroblast activation into cytokine-expressing iCAFs is not shown. Experimentally, upregulation of cytokines in response to cancer cell conditioned media is shown, but this analysis is limited to the mRNA level. The ability of these iCAFs to recruit and modulate the immune response is not shown at all in this manuscript.
RE: We thank the reviewer for the in-depth review and effort to point out the places that would benefit from additional experiments to strengthen the claims. Thus, we have addressed all concerns raised by the reviewer, and provide additional data and quantifications, that confirm all statements in the now slightly modified abstract. We also chose the more neutral term “immunomodulatory CAFs” instead of “inflammatory CAFs” for the iCAFs, as it describes the role of these CAFs in the TME.

Results:
Figures 1-2. These provide context to the scRNA-seq generated but do not provide insight into the main findings presented in the manuscript (relevant to fibroblast heterogeneity) and are therefore largely supplementary. However, these data do serve to illustrate how heterogeneous the cohort analysed by scRNA-seq is.
RE: Thank you for pointing this out. We agree that the main focus of our paper is on fibroblasts. Nevertheless, we believe it is essential to have the information of Figures 1 and 2 in the main figures rather than the supplemental information.
As far as the statement that the “scRNA-seq cohort is very small and heterogeneous” is concerned, we would like to point out that we have sequenced 10 tumor samples in our study, which is more than most other published studies in skin cancer, and also other cancer types, comprise (see in the newly added **Supplemental Table 1)**. This table also shows what readers and reviewers might overlook, namely, that even if a larger number of samples has been sequenced, not all patient samples contribute to the fibroblast clusters. For example, looking at the Ji et al. dataset, 10 SCC samples have been sequenced but less than 2% of the sequenced cells are fibroblasts, and out of those few cells more than 80% have been contributed by a single patient. Because we FACS-enriched for fibroblasts in our dataset, every patient sample (n=10) contributed a significant fraction of fibroblasts and the cell numbers are now added to Figure S3 (**Figure S3B**). Furthermore, we also used Smart-seq2 sequencing that provides a higher sequencing depth than 10x Genomics at the cost of a reduced cell number per specimen. Nevertheless, our average cell number per specimen is significantly higher than comparable Smart-seq2 studies from Tirosh et al. or Jerby-Arnon et al. (**Supplementary Table 1**).

**Manuscript**	**cancer type**	**specimen**	**total cells**	**CAFs**	**additional information**
Tirosh et al., Science 2016	melanoma	19	4,645	61	
Jerby-Arnon et al., Cell 2018	melanoma	33	7,186	106	
Ji et al., Cell 2020	SCC	10	48,000	882	>80% of CAFs from 1 donor
Guerrero-Juarez et al., Science Advances 2022	BCC	4	36,392	5,775	CAFs only from 2 donors
Yerly et al., Nature Communications 2022	BCC	5	28,819	809	

As far as the heterogeneity is concerned, it is not surprising that different mutations will lead to heterogeneity within the tumor cells. Looking at the stroma cells, however, we found that both the immune cells and CAFs from all patient samples intermingled, apart from the fact, that iCAFs are more abundant in late-stage tumors. To cement this finding, we performed *in situ* stainings in 36 independent tumor samples, confirming the scRNA-seq data analysis. With these

staining we not only validated the three CAF subtypes showing their presence and abundance (**Figure S6**), but also show their respective tumor tissue location in 36 independent tumor samples **(Figure S5)**.

Tirosh, Itay, Benjamin Izar, Sanjay M. Prakadan, Marc H. Wadsworth, Daniel Treacy, John J. Trombetta, Asaf Rotem, et al. "Dissecting the Multicellular Ecosystem of Metastatic Melanoma by Single-Cell RNA-Seq." Science 352, no. 6282 (April 8, 2016): 189–96. https://doi.org/10.1126/science.aad0501.

Jerby-Arnon, Livnat, Parin Shah, Michael S. Cuoco, Christopher Rodman, Mei-Ju Su, Johannes C. Melms, Rachel Leeson, et al. "A Cancer Cell Program Promotes T Cell Exclusion and Resistance to Checkpoint Blockade." Cell 175, no. 4 (November 2018): 984-997.e24. https://doi.org/10.1016/j.cell.2018.09.006.

Ji, Andrew L., Adam J. Rubin, Kim Thrane, Sizun Jiang, David L. Reynolds, Robin M. Meyers, Margaret G. Guo, et al. "Multimodal Analysis of Composition and Spatial Architecture in Human Squamous Cell Carcinoma." Cell 182, no. 2 (July 2020): 497-514.e22. https://doi.org/10.1016/j.cell.2020.05.039.

Guerrero-Juarez, Christian F., Gun Ho Lee, Yingzi Liu, Shuxiong Wang, Matthew Karikomi, Yutong Sha, Rachel Y. Chow, et al. "Single-Cell Analysis of Human Basal Cell Carcinoma Reveals Novel Regulators of Tumor Growth and the Tumor Microenvironment." Science Advances 8, no. 23 (June 10, 2022): eabm7981. https://doi.org/10.1126/sciadv.abm7981.

Yerly, L.; Pich-Bavastro, C.; Di Domizio, J.; Wyss, T.; Tissot-Renaud, S.; Cangkrama, M.; Gilliet, M.; Werner, S.; Kuonen, F. Integrated Multi-Omics Reveals Cellular and Molecular Interactions Governing the Invasive Niche of Basal Cell Carcinoma. Nat Commun 2022, 13 (1), 4897. https://doi.org/10.1038/s41467-022-32670-w.

Figure 3:
Fibroblast clustering shows a high degree of sample specific grouping in the UMAP projection. How were batch effects between samples handled and corrected for? If not (as seems to be the description in the methods) does applying such a correction impact the clustering?
RE: To assess the severity of the batch effect, we performed batch correction with Harmony version 0.1.0 and DonorID as the batch key. Subsequentl we calculated the LISI index (Korsunsky et al., 2019) to quantify the level of mixing of cells from different batches in each cluster and to compare the corrected versus non-corrected analysis (**Figure REV3**). The correction affected primarily the clustering of healthy and malignant melanocytes, and resulted in less clear separations of cell types such as keratinocytes and fibroblasts (**Figure REV3**). As all cells were processed the same (single-cell collection, SMART-seq2 library prep), we decided not to regress for batch effects to better preserve biologically meaningful information. While patient-specific effects are present among tumors cells, this is not the case for endothelial or immune cells. The patient-specific clustering of cancer cells is expected due to different mutations and CNVs in the tumors themselves, and should, thus, not be calculated away. As an example: In healthy and malignant melanocyte second level clustering (**Figure 2B and S2B**) we see patient-specific clustering of the malignant cells, which can be explained by strong genomic alterations found in our CNV analysis (**Figure 2C**). We see a separate cluster of healthy melanocytes <u>of all healthy samples</u>, and we see the non-neoplastic melanocytes of <u>all tumor samples</u> clustering together. This alone shows that batch correction is not necessary and might even mask true differences when applied. Thus, we did not apply batch correction to any clustering.

There are discrepancies between the expression of previously described papillary/reticular fibroblast markers and the clusters identified in this dataset. However, this is not adequately explained or addressed. It seems that very few reticular markers are strongly expressed by the "rFIB" cluster (Figure S3C).

RE: The majority of published markers for reticular and papillary fibroblasts have been determined from *in vitro* studies where fibroblasts have been extracted from upper or lower dermis, and only a few markers come from *in situ* stainings. In a comprehensive study from our lab, in which we have – in contrast to all previous studies – FACS-sorted distinct fibroblast populations and proven different functions (Korosec et al. 2019), we show that previously published markers for the respective subsets do not always match. This was also seen in recent scRNA-seq studies (e.g. Tabib et al. 2018, Philippeos et al. 2018), markers found in Tabib et al. have not been detected in the Philippeos et al. dataset, and vice versa. In summary, we show the expression of robust markers in the 2 subsets, such as the reticular fibroblast marker THY1/CD90, which is a discriminating marker between fibroblasts that can undergo adipogenesis,a hallmark of reticular fibroblasts. We have rephrased the text in the revised manuscript to explain this part better.

Tabib, Tracy, Christina Morse, Ting Wang, Wei Chen, and Robert Lafyatis. "SFRP2/DPP4 and FMO1/LSP1 Define Major Fibroblast Populations in Human Skin." Journal of Investigative Dermatology 138, no. 4 (April 2018): 802–10. https://doi.org/10.1016/j.jid.2017.09.045.

Philippeos, Christina, Stephanie B. Telerman, Bénédicte Oulès, Angela O. Pisco, Tanya J. Shaw, Raul Elgueta, Giovanna Lombardi, et al. "Spatial and Single-Cell Transcriptional Profiling Identifies Functionally Distinct Human Dermal Fibroblast Subpopulations." Journal of Investigative Dermatology 138, no. 4 (April 2018): 811–25. https://doi.org/10.1016/j.jid.2018.01.016.

Korosec, Ana, Sophie Frech, Bernhard Gesslbauer, Martin Vierhapper, Christine Radtke, Peter Petzelbauer, and Beate M. Lichtenberger. "Lineage Identity and Location within the Dermis Determine the Function of Papillary and Reticular Fibroblasts in Human Skin." Journal of Investigative Dermatology 139, no. 2 (February 2019): 342–51. https://doi.org/10.1016/j.jid.2018.07.033.

Notably, the markers for this population do clearly overlap with PI16+ "universal fibroblast" described by Buechler et al Nature 2022 and others, would this terminology be more accurate?
The paper from Buechler et al. 2021 describes two "universal fibroblast" subsets expressing either *PI16* or *Col15a1*. This study was performed in mouse tissue. However, in a study in human palatine tonsils (De Martin et al., Nature Immunology, 2023), the PI16+ cells were called "reticular fibroblasts". We find expression of *PI16* primarily in the rFIB cluster (**Figure REV4**). Thus, we suggest that the nomenclature "reticular" is more accurate than "universal".

Buechler, Matthew B., Rachana N. Pradhan, Akshay T. Krishnamurty, Christian Cox, Aslihan Karabacak Calviello, Amber W. Wang, Yeqing Angela Yang, et al. "Cross-Tissue Organization of the Fibroblast Lineage." Nature 593, no. 7860 (May 27, 2021): 575–79. https://doi.org/10.1038/s41586-021-03549-5.

De Martin, Angelina, Yves Stanossek, Mechthild Lütge, Nadine Cadosch, Lucas Onder, Hung-Wei Cheng, Joshua D. Brandstadter, et al. "PI16+ Reticular Cells in Human Palatine Tonsils Govern T Cell Activity in Distinct Subepithelial Niches." Nature Immunology 24, no. 7 (July 2023): 1138–48. https://doi.org/10.1038/s41590-023-01502-4.

Line 163, describes healthy fibs found on "transition" (denoting field cancerisation) to tumour. This comment requires further justification. What is meant by on transition?
RE: Thanks for this comment. In response, we used Monocle 2 (Qiu et al., 2017) and Monocle 3 (Cao et al., 2019) to analyze the developmental trajectory of fibroblasts and included the results as new **Figure 3D** and **3E**. The trajectory analyses revealed two differentiation paths from healthy fibroblasts: either towards mCAF/iCAF or toward *RGS5*$^{+}$cells. The analysis also shows that iCAFs are a differentiation endpoint, with mCAFs being an intermediate state, and thus it may be possible that iCAFs develop from mCAFs. At current, this remains an open possibility which needs to be confirmed (or refuted) with future in-depth experiments. Interestingly, adjacent skin cells are preferentially found in the healthy fibroblast branch or the

RGS5^{+} cell branch, and a small fraction in the mCAF/iCAF branch, which indicates that they are in a transitory position between healthy fibroblasts and CAFs.

Describing ACTA2 as a "key signature molecule" for myCAFs in transcriptomic data is not appropriate. De novo aSMA+ stress fibre formation in addition to elevated ECM deposition is the "key signature" for activated/myo-fibroblasts. The expression of ACTA2 at the transcriptome level has been mis appropriated by a number of studies to identify activated/myo-fibroblasts and (as described by the authors) in this context is a stronger marker of pericytes and smooth muscle cells (as demonstrated by the co-expression of MCAM and RGS5). However, the absence of elevated expression of ECM genes (e.g. collagen family members) precludes the conclusion that these are myoCAFs or comparable to those cells found in fibrosis.
RE: We agree with the reviewer that *ACTA2* at transcriptomic level has been misappropriated. However, we related our findings to previous literature. Of note, the *RGS5*$^{+}$ cells express several collagens, albeit at a lower level compared to matrix CAFs.

Description of ACTA2+ and FAP+ combination for identifying all CAFs is not novel or particularly informative given (as the authors describe) this strategy cannot differentiate between healthy mural cells and CAFs. Furthermore, this approach is then not validated or demonstrated further in the RNAscope/IHC analysis.
RE: Yes, we agree with the reviewer that ACTA and FAP have been used as CAF markers previously and that they are not uniquely expressed by fibroblasts, and therefore noted in the manuscript that these markers also label other cells. We explicitly included the markers here because when presenting our data at international conferences we have frequently been asked which markers could be used to detect all CAFs, which is why we included this finding from our scRNA-seq screen. I.e. various studies use single markers to detect or isolate fibroblasts or CAFs from tissues, and thus risk to lose a significant number and variety of CAFs in their analysis.

Figure 4:
As described above it remains unclear and variable between studies how myCAFs and pericytes are described based on transcriptome level analysis of CAFs. In figure 4 the authors seek to address this issue, which is a valuable endeavour. However, this analysis is quite cursory, consisting of discrepancies with the scRNA-seq data that remain unexplained, and therefore fails to adequately justify the conclusion that the RGS5+ cluster identified by scRNA-seq are in fact a mix of pericytes and CAFs. For example, the authors state "However, [RGS5] staining intensity was stronger in perivascular cells compared to stromal RGS5+ in the tumor tissue, which was not reflected in the scRNA-seq data". It is evident from Figure 4A that RGS5 is expressed in a subset of mCAF and iCAFs in the scRNA-seq data. So it is equally possible that the reduced expression of RGS5 in tumour stroma compared to perivascular regions could indicate that the cells found in tumour stroma reflect the mCAF/iCAF populations (not the "RGS5+" cluster). To address this question more robustly a broader panel of markers is required to specifically detect each subpopulation identified in the scRNA-seq and examine their spatial distribution, ideally across the whole tissue (rather than selected ROIs).
RE: Thanks for the thoughtful reflection. We agree, the fact that the *RGS5* RNA staining seen in perivascular cells is visually stronger may not be a distinctive characteristics but may rather reflect the fact that there the cell density is much higher compared to the dispersed CAFs in the stroma. However, the large number of *RGS5*+ in the stroma (not vessel associated) and our extensive new co-stainings with markers of all three subsets, show that mCAFs, iCAFs and RGS5^{+} CAFs indeed represent distinct populations **(Figure S6B)**. We also performed stainings with additional markers for all three populations (mCAFs: *COL11A1*, *PTGDS* and *MMP11*,

Figure 5A and REV5; RGS5$^+$ CAFs: TAGLN, **Figure 4A;** iCAFs: *MMP1* and several cytokines, **Figure 6A**.

Additionally, there is no explanation provided for the lack of PDGFRA expression detected in any region analysed. PDGFRA expression should have been detected in adjacent skin as it is highly expressed in healthy fibroblast populations. Were controls performed to confirm accurate detection of these markers by RNAscope?
RE: We do detect *PDGFRA* in all fibroblasts (except for *RGS5*$^+$ CAFs) in the scRNAseq data, and in all fibroblasts (including *RGS5*$^+$ CAFs) *in situ* with RNAscope, which was indeed less obvious in the original blue color choice; we now made it more bright/light blue (**Figure 4B, S4C**). Of note, while *PDGFRA* is strongly expressed in neonatal skin, the expression is low in adult skin, which is why FACS-sorting fibroblasts with anti-PDGFRα antibodies works well from neonatal mouse skin but not from adult tissue. We see a similar expression pattern in human tissue, and this is likely also a reason why *PDGFRA* is frequently not detected at all in 10X genomics scRNA-seq datasets (e.g. Tabib et al. 2018). Reassuringly, reanalysis of the Puram dataset also shows *PDGFRA* expression in the "CAF" subsets but not the "myofibroblast" and "intermediate fibroblast" population **(Figure 4C and REV6)**

Figure 5:
The analysis of potential ligand-receptor interactions is extremely speculative and should not be considered further than hypothesis generation. To justify the conclusions drawn regarding roles in immune modulation and tumour-stroma signalling mechanisms, requires both tissue analysis to demonstrate spatial autocorrelation of the ligand-receptor pairs at the protein level and functional analysis of intercellular interactions between the relevant cell populations.
RE: We agree, the receptor-ligand analysis alone allows only speculations about possible functions. The data we confirmed from this analysis is the expression of cytokines by iCAFs *in situ*, and by *in vitro* assays that CAFs synthesize cytokines and chemokines. We have included additional data showing that fibroblasts treated with conditioned medium from tumor cells are capable of activating T cells **(Figure 7),** thus confirming receptor-ligand interactions.

Figure 6:
The results showing tumour cell conditioned media inducing iCAF marker expression has been described previously in other cancer types (e.g. Ohlund et al Cancer Discovery 2017) so this finding is not particularly novel. The conclusion that aggressive tumours are solely capable of stimulating iCAF marker expression is interesting. However, this requires further evidence to demonstrate a significant correlation between iCAFs and tumour grade in tissues; and consistent demonstration (across more than one cell line) that conditioned media from well differentiated/non-aggressive skin cancer and normal epithelium fails to induce iCAF marker expression in this in vitro system.
RE: We expanded our experiments with an additional metastatic and a primary melanoma cell line (now in total 3 metastatic melanoma, 2 primary melanoma, and one cutaneous SCC cell line). Conditioned media (CM) of two metastatic cell lines (VM08 and VM15) and the SCC cell line induced cytokine expression of all tested cytokines in NHDFs. Conditioned medium of one metastatic melanoma cell line (VM26), did not induce cytokine expression in NHDFs **(Figure 6C)**, but NHDFs that were pretreated with their CM (as well as the other melanoma and cSCC cell lines) were still capable of activating T cells (newly added **Figure 7**). Of note, we also assessed if the CM from different cell lines induced mCAF marker gene expression, which was not the case **(Figure S9A).** Thus, we conclude that [at least] these cell lines cannot induce an mCAF phenotype *in vitro*, which we added as an interesting point for future investigations in the discussion.

Figures for Reviewers

REV1

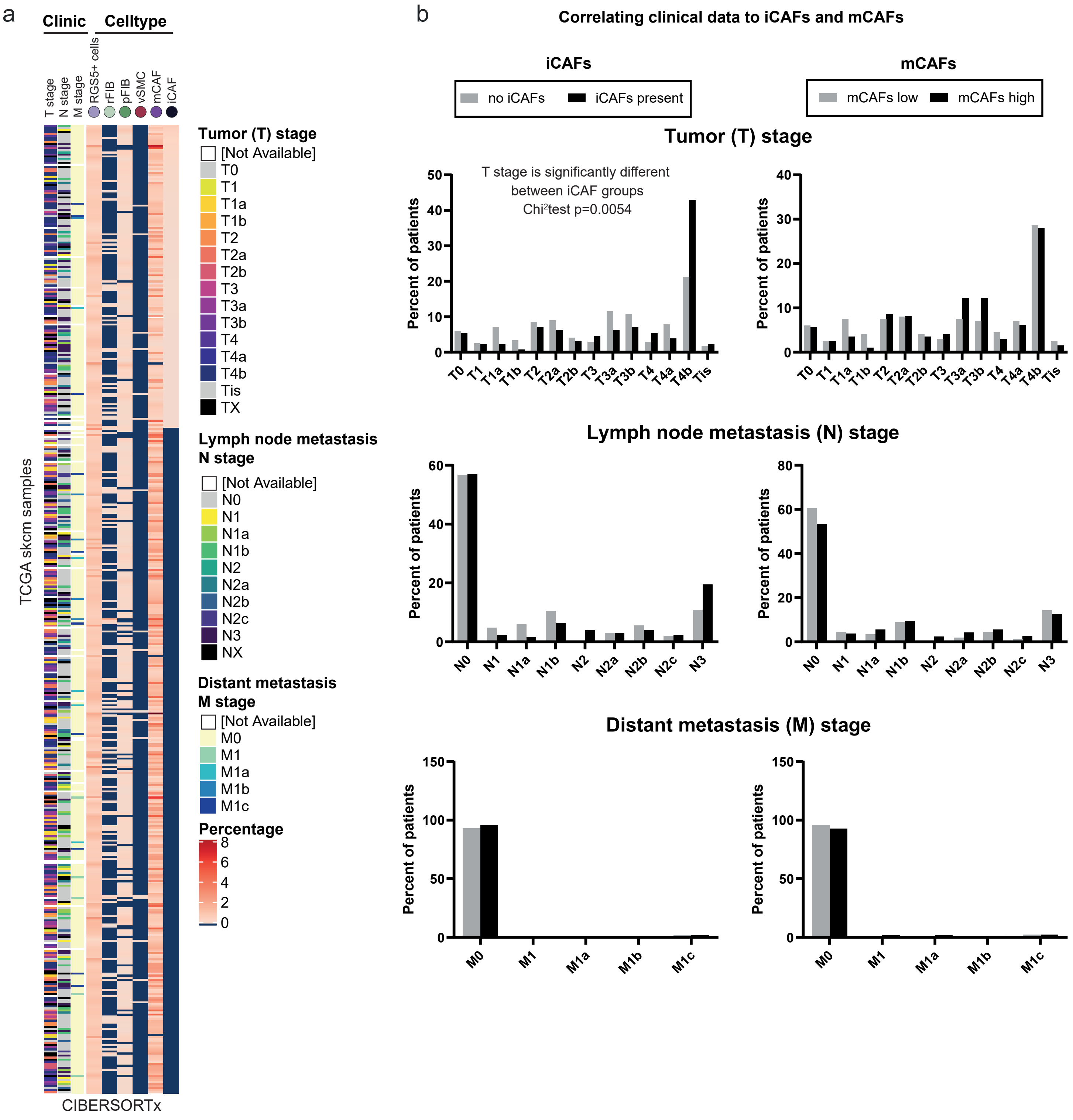

Figure REV1. Deconvoluting the TCGA skin cutaneous melanoma (skcm) dataset with CIBERSORTx (**a**) Heatmap showing percentage iCAFs, mCAFs, RGS5+ cells, pFIB, rFIB and vSMCs as well as clinical information of tumor (T), lymph node metastasis (N) and distant metastasis (M) in TCGA skcm patient samples. (**b**) TCGA skcm patient samples were separated into groups according to the abundance of iCAFs (no iCAFs (zero percent), iCAFs present (more than zero percent)) or mCAFs (mCAFs low, mCAFs high; cutoff median percentage of mCAFs) and correlated to the clinical information of the patients, shown as percent patients. Statistical analysis done by Chi-squared test.

REV2

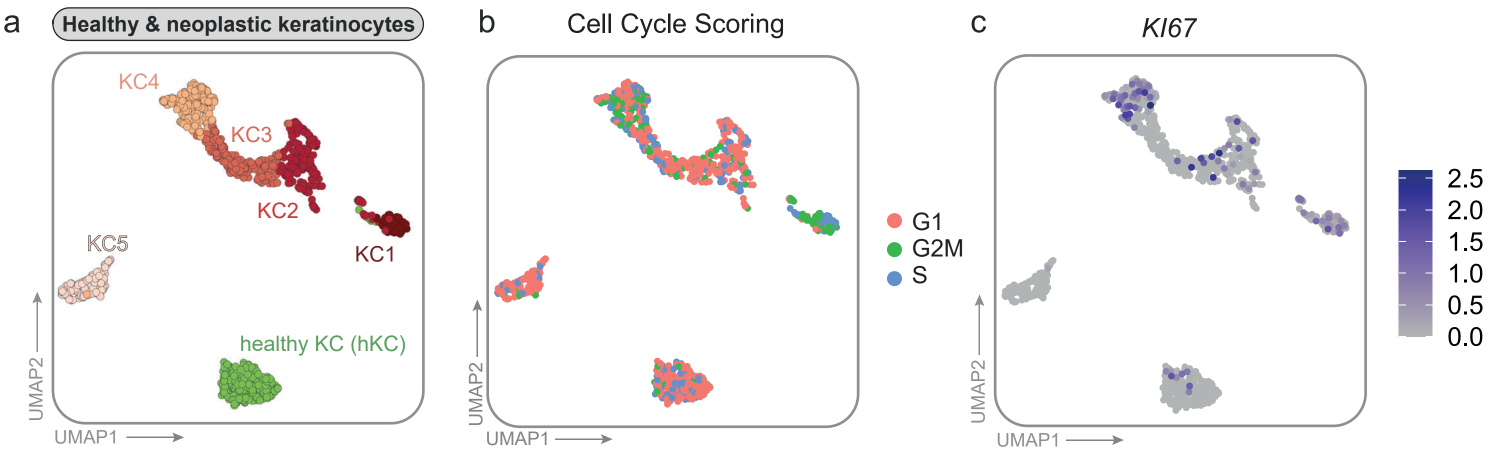

Figure REV2. Cell cycle scoring and expression of *KI67* in second-level clustering of healthy and neoplastic keratinocyes.
UMAPs showing (a) the different clusters of second-level clustering of healthy and neoplastic k e r a tinocytes, (b) cell-cycle scoring into G1, G2M and S Phase. (c) UMAP and violin plot showing the expression of *KI67*.

REV3

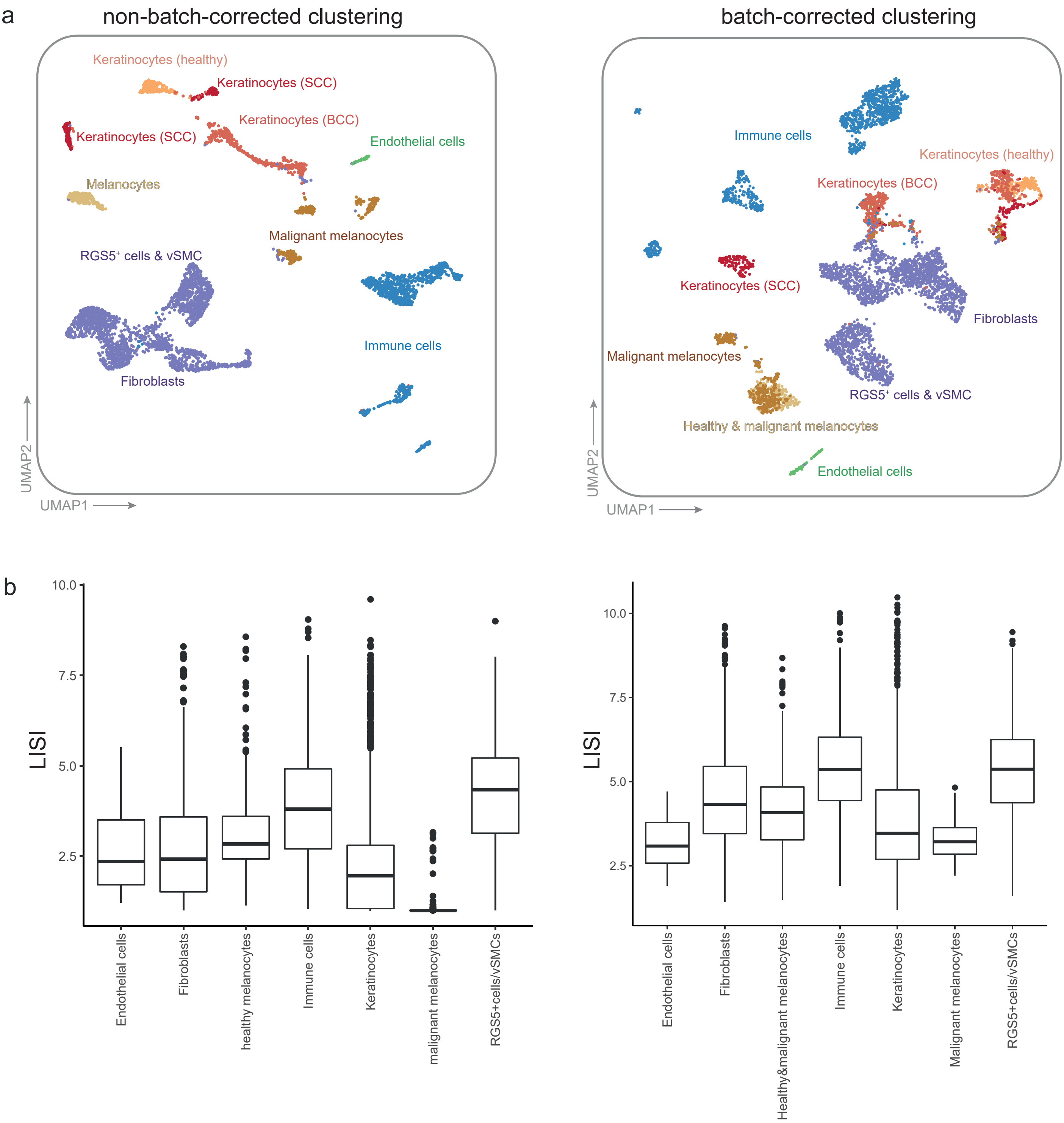

Figure REV3. UMAPs and Local Inverse Simpson's Index (LISI) for batch corrected and non-batch corrected clustering.
(a) UMAPs showing batch corrected and non-batch corrected clustering. (b) Boxplots showing the LISI values for batch-corrected and non-batch-corrected clusters.

REV4

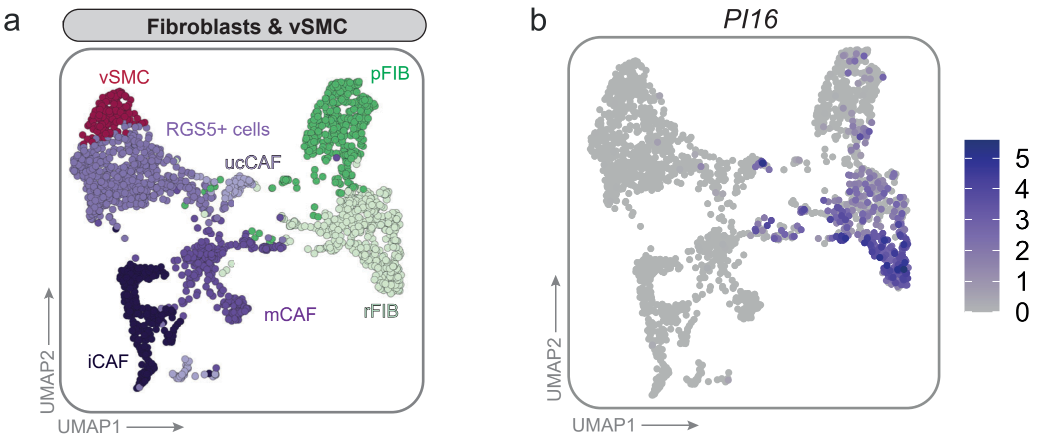

Figure REV4. Expression of *PI16* in second-level clustering of Fibrobasts&vSMCs. UMAPs showing (a) second-level clustering of Fibrobasts&vSMCs and
(b) expression pf *PI16* in reticular Fibroblasts (rFIB).

REV5

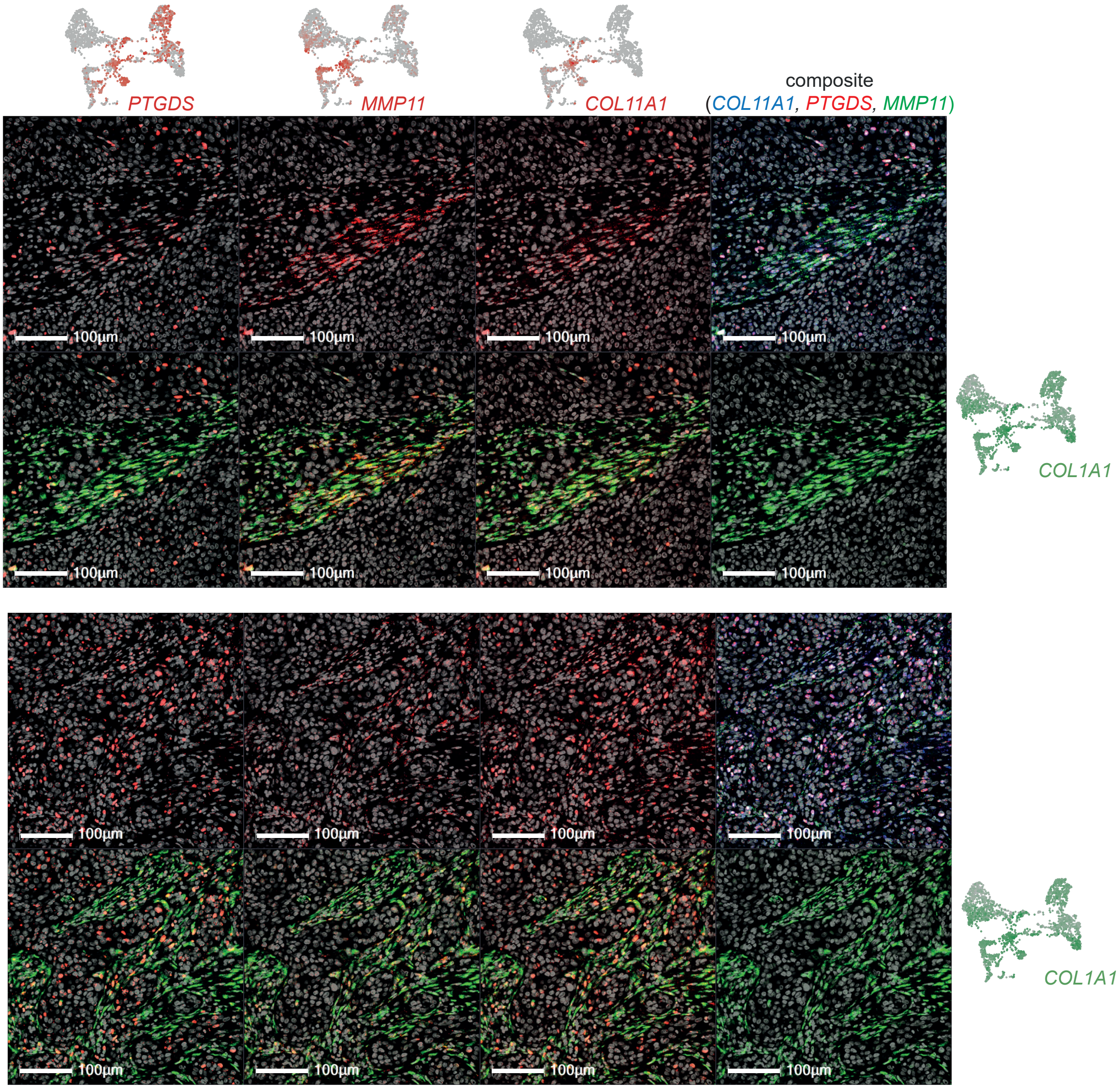

Figure REV5. *In situ* stainings of mCAFs with three different marker genes (*PTGDS, MMP11, COL11A1*).
Two representative images of RNAScope stainings for three different mCAF markers genes: *PTGDS, MMP11, COL11A1 shown as single stainings in red or in combination with COL1A1 (green). PTGDS (red), MMP11 (green)* or *COL11A1 (blue),* in composite staining.

REV6

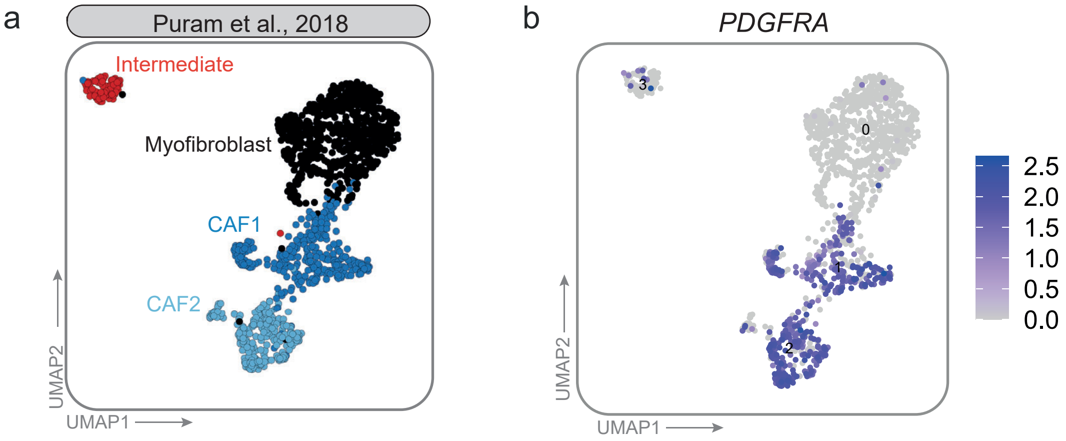

Figure REV6. *PDGFRA* expression is absent in myofibroblasts in an HNSCC data set (Puram et al.2018). UMAPs of CAFs from an HNSCC data set (Puram et al.2018) showing (a) the different CAF clusters of second-level clustering and (b) the expression of *PDGFRA* in CAF1 and CAF2

Point-by-point response

We would like to thank the reviewers for their close engagement with our findings and their insightful comments on our manuscript, and the editor for the opportunity to revise and resubmit our manuscript. As seen below in our point-by-point response, we have used the important input from the reviewers to address the remaining concerns. The questions have inspired us to go back and revisit key aspects, which have led to important clarifications and overall, a very satisfying outcome of this second revision of our manuscript.

Reviewer #2:

The authors have done tremendous amount of revision work that has further strengthen the paper. They have addressed all my previous comments.

RE: Thank you for the appreciation. We are pleased that we could build in the suggestions and address all concerns.

Reviewer #3:

We thank the authors for the efforts provided. Although the authors answered most of my points, they still did not show that every individual tumor sample actually contains tumor cells. Fig. 2A clearly shows that unsupervised clustering does not differentiate between normal and neoplastic keratinocytes, as illustrated by the mixed composition of KC2, KC3, KC4 and KC5. Moreover, CNV analysis as performed in Fig. 2C, does not identify neoplastic keratinocytes in various BCC and SCC samples. Finally, the authors do not show how far the expression of gene markers like PTCH1/2 are shared across individual tumor samples.
Our suggestion here would be to perform CNV analysis in individual tumor samples on keratinocytes only (and melanocytes only), using T cells (or stromal cells) as reference. Of note, proliferating cells which display a strongly modified transcriptome, should be excluded from the CNV analysis. Also, tumor markers expression like Ptch1/2, Gli1, MYCN, etc.. should be shown in individual samples. Of note, KC4 and KC5 express very low levels of KRT14, which suggests they are not proper keratinocytes.

RE: We are delighted that we have addressed most of the points raised by this reviewer and thank the reviewer for further suggestions of how to address the remaining concern.

We have updated the CNV analysis (Figure 2C) following the reviewers recommendation. To this end, we performed the CNV analysis for healthy and malignant keratinocytes, as well as for melanocytes and melanoma cells, using stromal cells (fibroblasts, vascular smooth muscle cells, pericytes) or T cells as a reference. Indeed, the CNV analysis reveals within all samples cells with and without chromosomal rearrangements/CNVs, indicating that all samples contain both healthy and neoplastic cells. In the manuscript, we present the calculation against stromal cells, and the CNV analysis against T cells is added as a figure for the Reviewer (**REV-A and B**). Using stromal cells or T cells as reference increased the number of CNV+ cells per sample in comparison to our previous calculation using all healthy cells as reference, as suggested by the reviewer. The estimation of CNVs in Figure S3 was calculated as previously described by Tirosh et al. 2016. In Figures S3A and S3B, the upper right quadrant shows the CNV+ cells for each individual tumor sample. Please note that the samples BCCII and SCCII comprise single or no CNV+ cells because they contain very few keratinocyte scRNA-seq transcriptomes that passed QC. In Figure S3A we also highlighted the cells with increased *PTCH1*/*PTCH2* expression (Figure S2A) that do not show CNVs. In the CNV estimation plots for melanocytes and melanoma cells (Figure

S3B) we highlighted melanocytes derived from cluster hMC (melanocytes derived from healthy skin) and tMC (melanocytes derived from SCC, BCC and from unaffected skin adjacent to melanoma), where most of the cells are found in the lower quadrants as expected (CNV- and undefined). We also added the plots showing *PTCH1, PTCH2, GLI1, GLI2, MYCN* expression in all clusters of healthy and malignant keratinocytes as well as for each individual patient sample to Figures S2A and S2B.

Further, we agree with the reviewer that the expression of *KRT14* in KC4 and KC5 is low, however, panKeratin expression clearly identifies these cells as keratinocytes in comparison to all other cell types in this study (**REV-C**). Thus, we performed further analysis for these two clusters to characterize them in more detail.

KC4 cells express in a scattered pattern a variety of keratins that are known hair follicle-associated keratins (**REV-D**). This suggests that KC4 contains keratinocytes from the different anatomical structures of the hair follicle (**REV-D**), which explains the co-clustering of healthy and tumor adjacent keratinocytes (**REF-E**) as well as KC4's cluster position next to the BCC keratinocytes (KC3) in line with the presence of PTCH1/2 and GLI1/2 expressing cells (Figure 2C and Figure S2A,B).

KC5 expresses high levels of keratins typically found in SCCs like *KRT7*, *KRT8*, *KRT18* and *KRT19* (Kurokawa et al., 2011). Interestingly, a recent scRNAseq study used *KRT7*, *KRT19*, *SFRP1* and *DCD* to identify luminal cells (Ganier et al. 2024) (**REV-F**). Since KC5 contains keratinocytes from tumor, healthy and unaffected adjacent skin samples, including cells with CNVs, we conclude that KC5 comprises both luminal (sweat- or sebaceous gland-associated cells) from healthy/unaffected skin and neoplastic keratinocytes.

We have updated the manuscript with these new results accordingly and thank the reviewer for guiding us to improve our CNV analysis and cluster annotations. Regarding keratinocytes, we would like to point out that our main aim in this project was the analysis of cancer-associated fibroblasts in skin tumors and thus, we optimized the enzymatic digestion protocol for fibroblast isolation. For healthy skin samples, we were able to use a different protocol to enrich for keratinocytes as we received surplus tissue. However, the tumor samples were limited to one 4mm punch biopsy per patient, which did not allow cell isolation with two protocols.

Reviewer #4 (Remarks to the Author):

I thank the authors for their efforts to address the concerns that I raised in the initial review of the manuscript. However, these attempts have not sufficiently resolved these concerns and similar issues remain with the revised version, which still presents largely descriptive findings and multiple conclusions that are insufficiently justified. Furthermore, the novelty here is limited as the phenotypes and functions described have been described previously. Perhaps there is a case to be made that these phenotypes have not previously been defined in skin cancer, but given their similarity to other tumour types it is debatable whether these findings are likely to receive significant interest from the wide readership of Nat Comms and may be better suited in a more disease specific journal.

RE: We are pleased that the reviewer appreciated our efforts to address the reviewer's concerns, most of which we had addressed in the last round of revision to their full satisfaction (31 out of 36 concerns raised by all 4 reviewers were satisfyingly resolved). We, however, respectfully disagree with the reviewer's notion that our findings fail to provide novel insight into fibroblast heterogeneity; our manuscript extends far beyond a common scRNA-seq data analyses describing cell types; we additionally provide extensive in-depth *in situ* characterization and confirmation, compare our analysis to a range of previous CAF studies and even follow up mechanistic aspects which is rather uncommon

for a “scRNA-seq focused” study. Based on the interest, feedback, and discussions with scientist around the world, we are convinced that our findings are not only interesting to a narrow skin field but to a wide audience of basic and clinical science of tissue and cancer research communities.

To address the reviewer’s final concerns, we have added the following new data/analyses to the paper:

1. We confirmed that normal fibroblasts and those stimulated with conditioned medium from cancer cells not only upregulate the expression of cytokines at the mRNA but also at the protein level, measured by LegendPlex analysis.

2. We included additional tumor samples (4x BCC, 5x SCC, 7x Melanoma) to former Figure S6A (now Figure 5D), which we stained with MMP1, COL11A1 and RGS5 (and COL1A1 RNA as a pan-fibroblast marker) RNA *in situ* to characterize their CAF content. Furthermore, additional tumor sections (5x BCC, 3x SCC, 2x Melanoma) were stained with CXCL2, CXCL8 and IL24 (and COL1A1 RNA as a pan-fibroblast marker) to detect cytokine-expressing CAFs (for former Figure 6B).

3. We assessed and re-assessed the regions of interest (ROIs) of the newly added and previous tumors (n=52) through additional pathology consultancy. This analysis agrees with the trend shown before and confirms statistically significant differences in iCAF content between nodular and infiltrative BCC and between low-grade and high-grade melanoma.

4. We added large-field *in situ* images using multiplex RNA-FISH showing the distribution of cytokine-expressing CAFs (new Figure S7B) and of mCAFs, iCAFs and total CAFs in the whole tumor tissue of representative samples (Figure 5C) of each skin cancer subtype.

5. The presentation of the newly added Figure 7 in revision 1 comprising the T cell proliferation/activation assays was updated for a better visualization of the data. In addition, we included CAFs that were isolated directly from a melanoma biopsy and have not been in contact with conditioned medium from cancer cell lines (pMel CAFs) into the assays, and these CAFs were also capable of activating T cells. A scheme for the experimental setup as well as FACS gating strategies and FMOs were added.

Nearly all statements from the abstract regarding results presented in this manuscript are still not adequately justified:
RE: We have re-assessed every term in the abstract and adjusted our word choice to avoid any potential overstatement. Additionally, we have consulted a statistics expert (Dr. Robin Ristl, Institute for Medical Statistics, Medical University of Vienna) to evaluate and define the most appropriate statistical tests for former figures 6B, 7B,C, S7A and S13C (now presented in Figure 5C, 5F, 7B, 7C and S12C) which were recalculated if necessary.

• “Matrix CAFs (mCAFs) ensheath tumor nests and synthesize extracellular-matrix to prevent T cell invasion.”
o This is a function that has been attributed to mCAFs in multiple studies so I don’t have too much difficulty accepting this conclusion. However, based on the data presented in this manuscript alone this is not convincingly shown. Figure 5D shows a slight reduction in CD3s within tumour nests where high CAFs or mCAFs are found. Therefore, this doesn’t demonstrate a specific role for mCAFs in limiting CD3+ cell accumulation in tumour nests. Furthermore, this is purely a correlative association and therefore insufficient to justify the statement above.

RE: Indeed, the analysis shows that high numbers of mCAFs correlate with low numbers of CD3+ cells within tumor nests, which has always been statistically significant and is underpinned by high quality

in situ stainings. Of note, linear regressions of the data reveal that there is no correlation with iCAFs. In response to the reviewer, we have increased the sample size, updated the data presentation in former Figure 5D (now Figure 5F), and toned down the wording in the abstract.

• "Immunomodulatory CAFs (iCAFs), which express proinflammatory and immunomodulatory factors, are only detected in high abundance in aggressive tumors."

o The data presented shows that this is not the case. In fact, the new data provided in Figure S6A shows that there is no significant difference in iCAF abundance between the "aggressive" tumour subtypes compared to the earlier stages.

o The statistically significant difference shown in Figure 6B, is based on Chi squared analysis of multiple iCAFs vs no/single iCAFs. However, this Chi squared test approach to determining statistical significance is not sufficient to assess how consistently these differences are observed across biological replicates and therefore inappropriate to support the conclusion drawn. Additionally, the precise definition for what the categorical variable (multiple iCAFs vs no/single iCAFs) represents and how it was assigned is not clearly described.

RE: We agree that the data showing CAF-abundance differences between tumor types in former Figure 6A lacked clarity and the CAF difference in former Figure S6A was not statistically significant even though there was a clear visible trend of increased numbers of iCAFs in late-stage tumors compared to early stage tumors. To resolve this concern, we have consulted dermatopathologist Prof. Dr. Peter Petzelbauer to re-evaluate each end every tumor sample (n=68), and to determine/verify their classification once more (nodular BCC/infiltrative BCC, well-differentiated SCC/poorly-differentiated SCC, low-grade melanoma/high-grade melanoma). Moreover, we have stained 16 additional samples and included them into our analysis.

First, it is important to point out that this *in situ* quantification is particularly challenging because skin cancers have a generally high variability/heterogeneity in their tumor tissue morphology, and superficial regions are prone to ulceration. Skin tumors display heterogeneous morphology both within a single tumor (i.e. superficial tumor areas versus invasive front) and among distinct cancer subtypes. Thus, the biological difference may not be well/best captured in numbers. For this reason, we added exemplary large-field spatial visualizations of the CAF subsets for each of the 6 tumor categories (nodular and infiltrative BCC, well- and poorly-differentiated SCC, low- and high-grade melanoma), which show a marked difference in the CAF patterns from lower to higher malignancy, including a higher iCAF density in the more aggressive variants of the respective skin cancer subtypes (Figure 5C and 5D). *In situ* localization further shows that mCAFs are present in all tumors but are detected in high density at the tumor-stroma border especially in nodular BCC and well-differentiated SCC. For quantification, the dermatopathologist suggested to exclude the ulcerated areas, and scarring areas from preceding biopsies. Therefore, the dermatopathologist selected several ROIs per tumor for CAF quantification (former Figure S6A, now Figure 5D) within the tumor and at the invasive front to capture <u>representative fields of the whole tumor</u>. These ROIs were analyzed in an unbiased manner using HALO software. The CAF numbers from all ROIs per tumor were summed-up and each tumor is presented as one datapoint (CAFs/mm^2). In consultancy with statistician Dr. Robin Ristl (Institute for Medical Statistics, Medical University of Vienna), statistical analysis was performed using the Mann-Whitney test between 2 groups. The new data, which now include 52 tumor samples in total (additional samples: 4x BCC, 5x SCC, 7x melanoma) show statistically significant increases in iCAFs in infiltrative BCC compared to nodular BCC and in high-grade melanoma compared to low-grade melanoma (now Figure 5D). In SCCs the trend is the same but the difference in iCAF numbers between well- and poorly differentiated SCCs is not statistically significant.

In summary, our meticulous new quantification of iCAFs in the whole tumor tissue together with the large-field multiplex RNA-FISH *in situ* images is at current a best possible representation of CAF heterogeneity in skin tumor samples (new Figure 5C and 5D), making the original less-detailed CAF quantification Figure 6B redundant and, thus, this Figure has been removed. The manuscript text and the abstract were updated accordingly, and the challenge of this *in situ* quantitative analysis was exploited in the discussion.

• "Strikingly, iCAFs but not tumor cells are (apart from immune cells) the exclusive cell type producing chemokines and, thus, play a key role in immune cell recruitment and activation."
o The role of iCAFs in immune cell recruitment to tumours has not been demonstrated or even analysed.

RE: Many papers have shown which cytokines are necessary to recruit immune cells to a tumor. Thus, we believe that this is an established fact and does not need to be addressed again. Besides, our strongest argument is that in contrast to the notion suggested by previous bulk RNA-seq studies stating that the tumor cells play a major role in immune cell recruitment, our dataset reveals that it is the fibroblasts but not the cancer cells that produce the largest variety and generally highest amount of chemokines and cytokines. We have rephrased the abstract to: "iCAFs express unexpectedly high mRNA and protein levels of cytokines and chemokines, pointing to their integral role in immune cell recruitment and activation."

o The production of chemokines and cytokines has only been shown at the transcript level and is not confirmed to result in protein secretion, which would be critical to a functional role in immune cell recruitment and/or activation.
RE: We thank the Reviewer for the suggestion to confirm the expression of the chemokines/cytokines on protein level. To this point, we collected the supernatants from NHDFs that were stimulated with conditioned medium (CM) from cancer cell lines, which we then analyzed with LegendPlex, a FACS-based approach for the detection of protein levels. We have used two kits with pre-defined panels of secreted proteins (Human Essential Immune Response #740930 and Human Proinflammatory Chemokine Panel #740985, BioLegend) and quantified the secreted protein of 26 chemokines/cytokines. We confirmed that CM-treated fibroblasts express CXCL1, CXCL5, CXCL8 (IL8) and CCL2 not only on RNA but also on protein level (Fig. S11B), and detected protein expression of several additional chemokines and cytokines. Please note that only part of the qPCR measured chemokines/cytokines were covered by the two pre-defined LegendPlex kit panels. Further, the fibroblasts were stimulated with cancer cell-derived CM for 72h, then washed carefully and fresh medium was added and collected 48h later (Figure S11A). Thus, the cells were without stimulus for 2 days before their supernatant was analyzed, which explains why the protein-level differences are less pronounced than at the mRNA level (former Figure 6C, now Figure 6D). We have also included a figure for the Reviewer showing the chemokine/cytokine protein levels in the cancer cell-derived CM (Figure REV G,H). Here, the levels of secreted proteins (Figure REV G,H) are comparable to the expression levels on transcriptomic level (former Figure 6C, now Figure 6D) as RNA and protein expression were analyzed at the same time-point.

o The analysis of iCAF's role in immune cell activation (Figure 8) is very limited and (to support the importance of the point made above) the only conditioned media treatment shown to generate fibroblasts that significantly increased CD4 activation was from VM26, which is the condition where very few of the iCAF genes were upregulated in Figure 6. Therefore, calling into question whether there is any link between these co-culture assays and the transcriptomic profile of the fibroblasts used or analysed in human tissue samples.

RE: We appreciate this Reviewer's critical reflection of our findings. We would like to point out that the cytokines shown in former Figure 6C (now Figure 6D) are CXCR1/2 ligands that are known to recruit innate immune cells but not T cells. Thus, we do not expect a link between those cytokines shown in Figure 6D and the assays in Figure 8 (i.e. Figure 7 was meant), which address the potential of fibroblasts and CAFs to activate T cells.
To the best of our knowledge, while different publications, especially single-cell transcriptomic studies, may predict a role for fibroblasts in modulating immune responses, functional assays showing their capability to activate T cells have not been provided. In our study we demonstrate that primary fibroblasts isolated from healthy skin are capable of activating T cells (Figure 7B,C and S12C), and that this potential is enhanced when fibroblasts are exposed to the secretome of cancer cells. This enhancement is NOT limited to VM26 but is also detected with VM15 and VM19 (significant differences depending on T cell subset and time-point or assay). Please, see detailed explanations in the next paragraphs below.

Importantly, CAFs that were isolated directly from a melanoma biopsy and have not been in contact with conditioned medium from cancer cell lines (pMel CAFs), were also capable of activating T cells. We have included these additional data in Figure 7B,C and S12B. Preliminary analysis from our lab further shows that the three other melanoma cell lines express high levels of *IL1B*, while VM26 does NOT express *IL1B* but instead high levels of *IFNy*, indicating a distinct mechanism underlying this melanoma cell line which probably has a different mutation. We have rephrased the statement about the difference between VM26 and the other cell lines in the manuscript text to make this clear for the readers.

o This analysis also many technical limitations, *Further comments on this are provided below.
RE: We have addressed the technical limitations raised by the reviewer, as explained below.

• "Mechanistically, we show that cancer cells transform adjacent healthy fibroblasts into cytokine-expressing iCAFs, which subsequently recruit immune cells and modulate the immune response."

o In addition to the comments above, there are unclear discrepancies between the mechanism proposed from in vitro experiments using conditioned media and the trajectory analysis performed using scRNA-seq data. Trajectory analysis shows mCAFs to precede iCAFs in the differentiation trajectory. So how do the authors reconcile this with the findings that cancer cell conditioned media induce iCAF markers but not mCAF markers? Is it possible that in the tumour microenvironment an entirely different mechanism is active, involving immune cell mediated iCAF activation? Furthermore, the data provided to show that metastatic cell lines induce iCAFs and primary lines do not are inconsistent, as described above with VM26 CM not capable of inducing upregulation of iCAF marker genes.
RE: Trajectory analysis solely predicts a differentiation trajectory but without in vivo lineage tracing that proves this differentiation pathway/ phenotype switch, it remains what it is - a prediction. Second, to us it is not necessarily surprising that the CM of melanoma cell lines induced an iCAF but not an mCAF phenotype; which we rather would expect from CM of BCC cells (BCC is more likely to induce an mCAF phenotype than an iCAF phenotype). However, so far we did not get hold of BCC cell lines to prove this hypothesis.

Obviously, the *in vitro* setting does not entirely recapitulate the complexity of the native TME, and we cannot exclude that direct interaction with immune cells modulates the CAF phenotype. Nevertheless, the immune cells need to be recruited to the tumor in the first place. So far, cytokine/chemokine secretion and thus immune cell recruitment has been attributed to cancer cells and immune cells (due to data from bulk sequencing studies); what we emphasize is that the capability of CAFs to secrete

cytokines/chemokines also play an important role in shaping the immune composition in the TME. Importantly, CAFs that were isolated directly from a melanoma biopsy and have not been in contact with conditioned medium from cancer cell lines, were also capable of activating T cells. We have included these additional data in Figure 7B,C and S12B.

* Activation of T-cells shown in Figure 7 has multiple limitations.
• This analysis should be conducted with positive and negative (Isotype/FMO) controls.
o Without showing these controls it can't be determined what degree of staining reflects activation.
o It is likely that the MFI measurements used for quantification in Figure 7B are not appropriate to analyse differences in activation, due to distinct positive (or bright) populations shown in the histograms. This non-gaussian distribution renders the MFI an inaccurate value for summarising the data.
o It should be assessed whether the bright population represents an active population using a positive control (e.g. CD3 and CD28 stimulation) and then each condition should be quantified as the percentage of CD4/CD8 that have been activated.
RE: We thank the reviewer for carefully reviewing the new figure, and apologize that its data presentation has not been clearer in the last submission. Isotype and FMO controls were of course included in all experiments and are now provided in Figure S12A. As indicated in the methods section, a CD3/CD28 T cell activator was added to the cultures to provide a baseline activation. We have changed the presented data from the MFI measurements to percentages of proliferating/activated cells, shown in fold change normalized to NHDFs from each individual experiment. We also changed the layout of the histograms that shows respective FMO controls (Figure S12A). Furthermore, as recommended by the statistics expert, we performed Student's T tests comparing NHDFs and pMel CAFs to T cells alone, and confirmed statistically significant increases in T cell proliferation and early T cell activation (Figure 7B,C). Comparing cancer CM-treated NHDFs to control CM-treated NHDFs shows a further increase in CD4+ T cell proliferation (statistically significant with CM derived from VM15 and VM26), in CD8+ cell proliferation (statistically significant with CM derived from VM15, VM26 and VM19; Figure 7B), as well as in early CD4 and CD8 T cell activation, which is statistically significant with VM15- and VM19-derived CM for CD4 T cells, and with VM15-derived CM for CD8+ T cells (Figure 7C). Furthermore, late activation of CD4+ T cells measured at 96h was significantly enhanced by CM derived from VM15, VM26 and VM19 (Figure S12B).

• The results statements accompanying this figure are not also adequately justified or simply incorrect.
o "VM15, VM26, VM19, VM25 were more potent to induce proliferation in CD4 or CD8 T cells than the corresponding untreated NHDFs and cancer cells alone (Figure 7A)."
♣ This is clearly not true for VM19 and VM25 and presumably not significant for VM15 and VM26.
RE: We apologize, this statement was indeed wrong, which we have now corrected.

o "Early (CD69) and late (CD45RO) activation markers were upregulated on CD4 and CD8 T cells after 24h or 96h when co-cultured with pretreated NHDFs"
♣ This is true compared to T-cells alone, but this is not the relevant comparison.
♣ Very few changes are significant when considering the comparison to untreated NHDFs (the appropriate comparison for the conclusions drawn).
RE: We apologize for this generalized statement and have now revised the statements describing the results for each time point and T cell subset separately. As explained above, NHDFs without stimulation with the secretome of melanoma cells are capable of activating T cells (Figure 7B,C), highlighting the role of fibroblasts per se in immunomodulation. Depending on the T cell subset and the cancer cell line from which the CM was derived, this potential was enhanced by induced iCAFs, as described above in

detail.

• The figures seem to be cropped and lack axes labels.
RE: We apologize that some axes labels were missing, now updated. The figures look indeed cropped but they were not. The new layout of the most recent FlowJo software directly provides this as an output. However, we display the graph now using the classical layout to help the readers grasp the results better.

• The legend is not sufficiently detailed – what does each point represent in these graphs?
RE: We thank the reviewer for pointing out that the figure legend lacks detail, which we have provided in the revised manuscript. In addition, we have added schemes of the experimental setup.

Figures for Reviewers

A

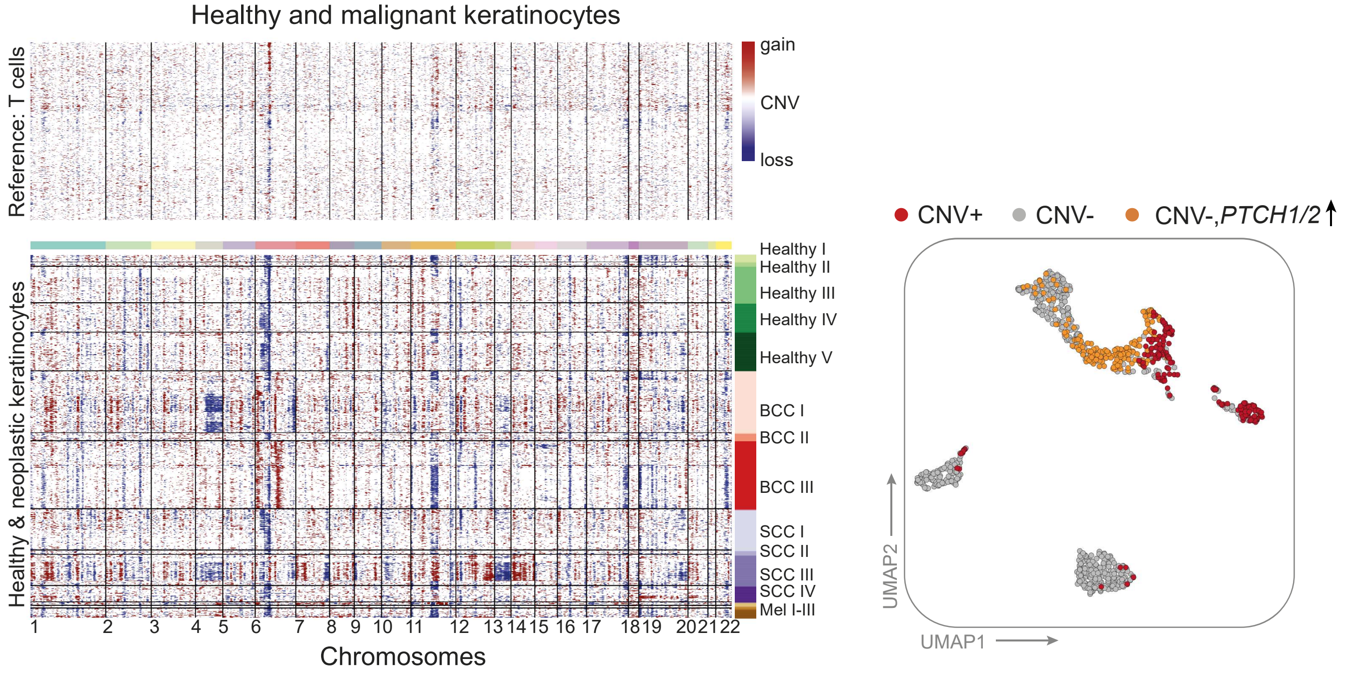

B

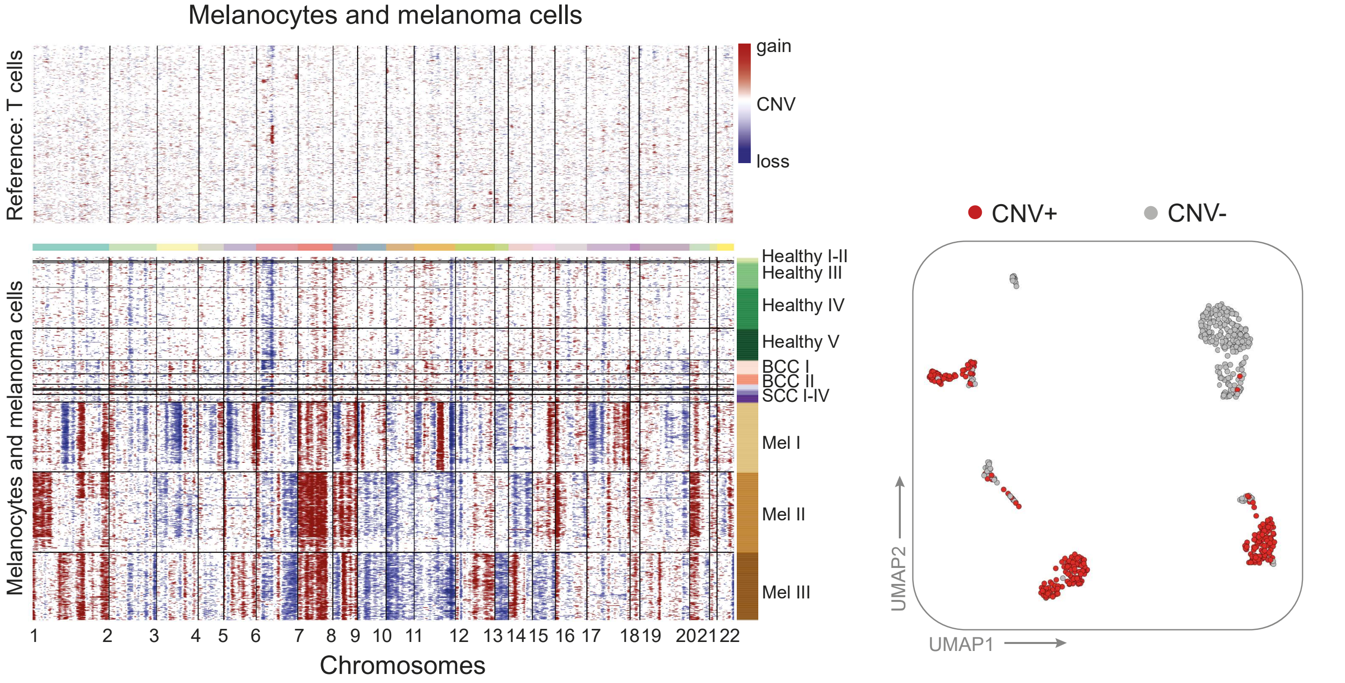

C Expression of panKeratin in all subpopulations of the dataset

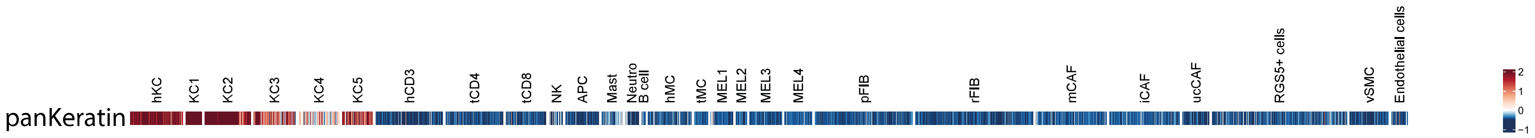

D

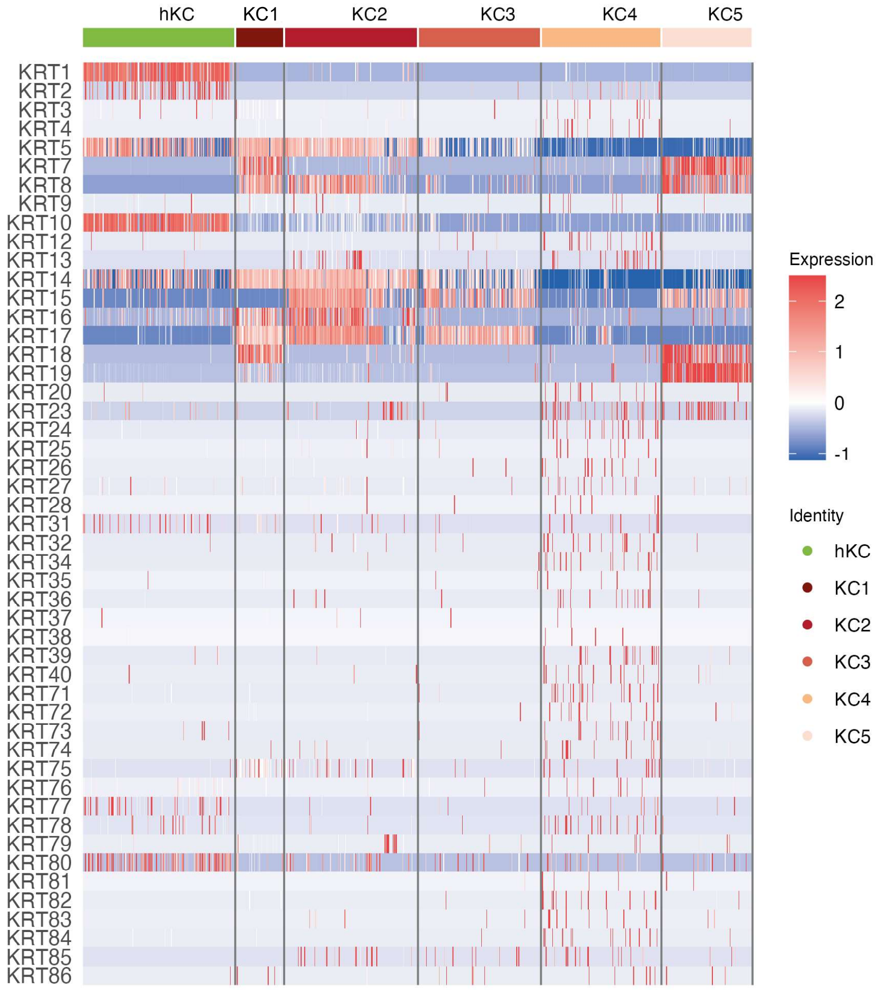

[figure redacted]

Jürgen Schweizer, et al. 2007, Experimental Cell Research
Hair follicle-specific keratins and their diseases

E Cells of KC4 and KC5 grouped by sample category and donor

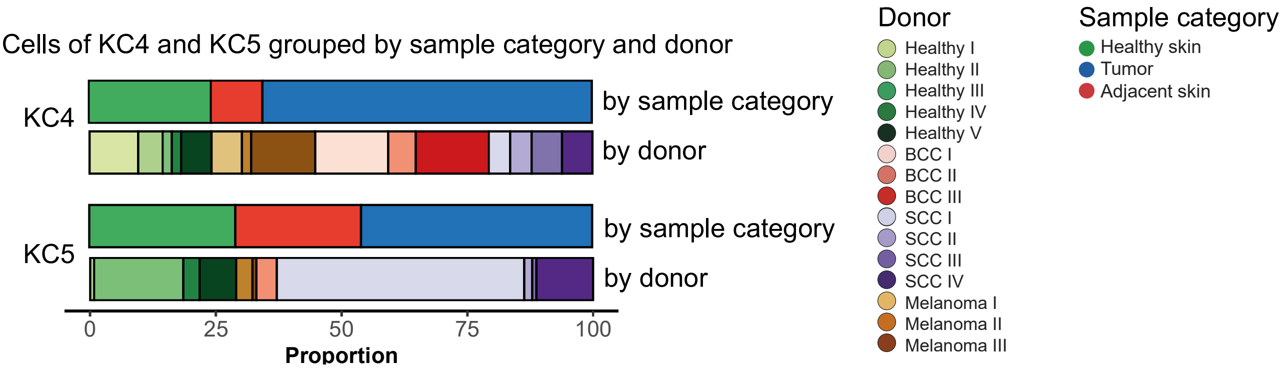

Markers for secretory luminal cells (Ganier er al. 2024)

[figure redacted]

G Experimental setup

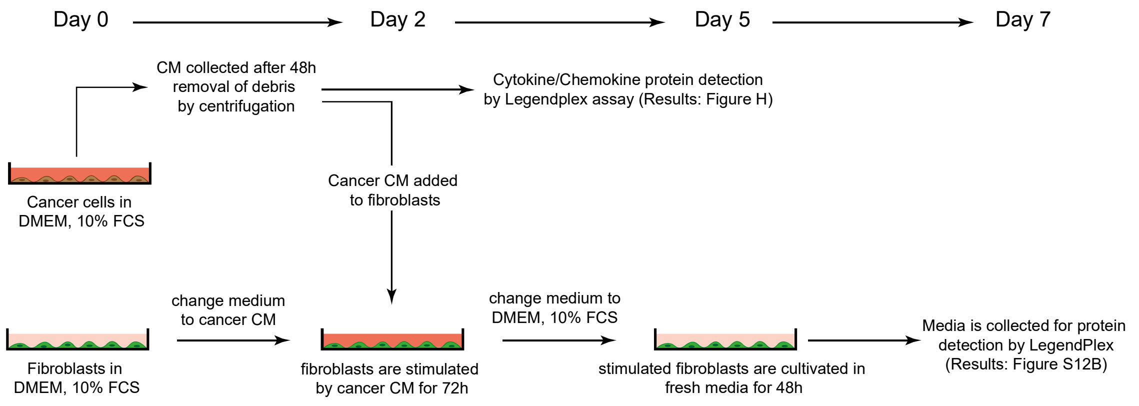

H Protein levels of chemokines and cytokines in cancer cell-derived conditioned medium (CM)

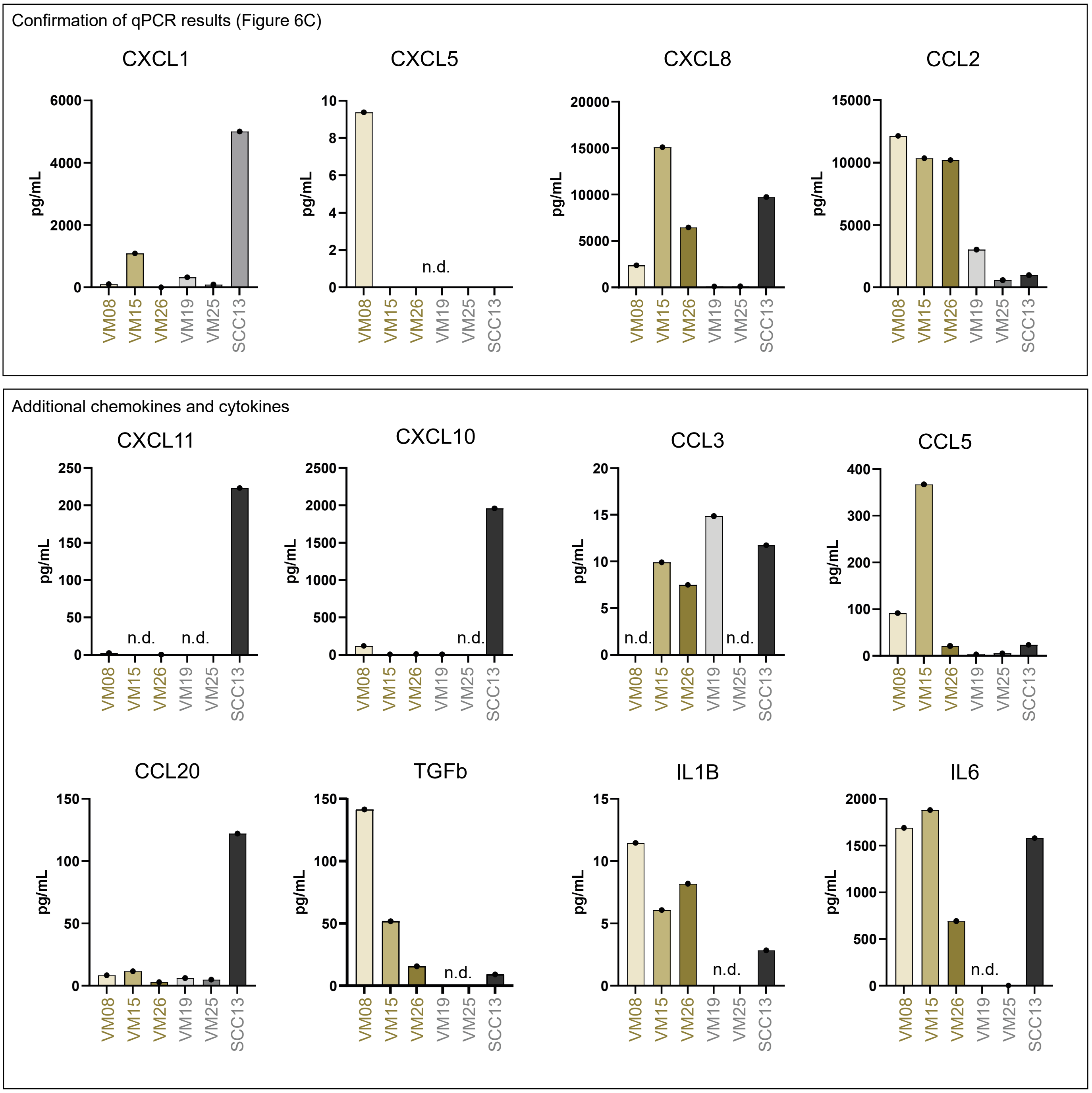

n.d. - not detected/below lowest concentration of standard

Point-by-point response

Once more, we would like to thank the reviewers for their close engagement with our findings and their insightful comments on our manuscript, which helped to strengthen the manuscript's overall impact and scientific rigor. We have incorporated your final suggestions as outlined in detail below.

Reviewer #3:
The authors satisfactorily addressed my points.
Minor point:
The authors claim that comparable scRNAseq addressing fibroblast heterogeneity in skin cancer are missing or included few or no fibroblasts. I do not agree with this assumption. First they are some addressing CAF heterogeneity, including some non-cited (doi.org/10.1038/s41467-023-41141-9). Second, the number of CAF included in each dataset does not necessarily correlate with the quality of the dataset. The authors should either rephrase or show that their dataset highlight previously unidentified heterogeneity in skin CAF.

RE: Thank you for the continued in-depth feedback! We are happy that we have satisfactorily addressed this Reviewer's comments. Addressing the last minor point, we have now rephrased our statement about previous scRNAseq studies in skin cancer and included the missing reference.

Reviewer #4:
I would like to begin by expressing my appreciation to the authors for their diligent efforts in revising the manuscript in response to previous comments. Your commitment to enhancing the clarity and quality of the research is commendable and reflects a strong dedication to advancing our understanding of this topic.
The revised manuscript now robustly demonstrates most of the points raised in earlier feedback. However, I have identified a few remaining discrepancies between the data presented and their description in the text. I believe addressing or clarifying these points prior to publication will further strengthen the manuscript's overall impact and scientific rigor.
Below, I outline these areas of concern, along with suggestions for potential improvements:

RE: We would like to thank this Reviewer for the continued engagement and very thoughtful feedback, which certainly has strengthened this manuscript! All final concerns were addressed following the suggestions as outlined below.

1) Linear regression analysis – p10 lines 341-343. "Of note, while total CAF and mCAF numbers negatively correlate with CD3 cells/mm² in tumor nests, iCAF numbers did not (Linear regression: total CAFs: R^2=0,039; mCAFs: R^2=0,040; iCAFs: R^2=0,009)"
These R^2 values suggest a modest correlation, indicating that mCAF abundance accounts for approximately 4% of the variance in CD3 accumulation within tumor nests. While this could potentially have a biological effect, it would be beneficial to provide further details on the strength and statistical significance of these correlations to better understand the implications.

RE: We have added the respective p values to the figure to provide further details on the strength and statistical significance of these correlations.

2) LegendPLEX validation (Figure S11) – p11 lines 391 – 393
"Importantly, we confirmed the expression of several cytokines and chemokines by fibroblasts and induced iCAFs on protein level with LEGENDplex assays (Figure S11)."
The results in Figure S11 appear to show that the increased expression levels observed in NHDFs treated with VM08 and VM15 conditioned media at the transcript level are not consistently reflected at the protein level. It would be helpful to revise this section of the text and relevant sections of the discussion to more accurately represent the LegendPLEX data, which clearly has implications for understanding the mechanism involved in these cell's role in the T-cell activation assays or alternate functions.

RE: We thank the Reviewer for the suggestion to clarify differences between the chemokine expression shown on the transcriptomic and protein levels. We have included an explanation in the text.

3) T-cell activation by iCAFs (Figure 7) – p11 line 403-404
The statement on p11 lines 403-404 "This potential to activate T cells was enhanced when fibroblasts were exposed to the secretome of cancer cells", could be refined to better reflect the heterogeneity observed across different cancer cell lines. While the subsequent sentences do elaborate on this variability, it may be more accurate to revise the initial statement to avoid potential misinterpretation. Additionally, the interpretation of these data might be enhanced by more clearly connecting the qPCR results with the T-cell activation experiments. For instance, it could be noted that VM15 conditioned media, which consistently enhanced cytokine transcript levels in the qPCR experiments, also consistently increased T-cell proliferation and activation. This connection could help strengthen the overall narrative.

RE: Thank you for the thoughtful comment. We rephrased the section in the results according to this Reviewer's suggestions and also added a sentence about the link between T cell activation and cytokine transcript levels into the discussion.

4) Lastly, it would be prudent to double-check the statistical analyses presented in Figure 7. In particular, the significance reported for Fig 7B (proliferating CD4s) between NHDFs and VM19 CM treated NHDFs may warrant review. It would also be helpful to clarify whether Welch's correction was applied in the unpaired Student's t-tests to account for unequal variances between groups. If this correction was not applied, the analyses should be updated accordingly.
I would also recommend that the authors clarify the expected role of T-cell activation in tumor progression, perhaps in the discussion. Given that iCAFs were associated with aggressive tumors, it is somewhat unexpected that their primary function would be to activate T-cells, which generally have a tumor-suppressive role.

RE: There was indeed an obvious mistake in labelling the data points with significance asterisks in Figure 7B. We apologize for the mislabeling! We have updated the statistical analysis with the Welch's correction, and carefully checked all p values and labels again. The individual p values for the Welch's correction are listed in the source data file.

Furthermore, we totally agree with the note about the expected role of T-cell activation and the role of iCAFs in modulating T cell function in tumor progression. We have now included a new paragraph on this part in the discussion.
